# Genomic amplification of the caprine *EDNRA* locus might lead to a dose dependent loss of pigmentation

**DOI:** 10.1038/srep28438

**Published:** 2016-06-22

**Authors:** Fiona Menzi, Irene Keller, Irene Reber, Julia Beck, Bertram Brenig, Ekkehard Schütz, Tosso Leeb, Cord Drögemüller

**Affiliations:** 1 Institute of Genetics, Vetsuisse Faculty, University of Bern, Bremgartenstrasse 109a, 3001 Bern, Switzerland; 2 Department of Clinical Research and Swiss Institute of Bioinformatics, University of Bern, Murtenstrasse 35, 3008 Bern, Switzerland; 3 Chronix Biomedical, Goetheallee 8, 37073 Göttingen, Germany; 4 Institute of Veterinary Medicine, Georg-August-University Göttingen, Burckhardtweg 2, 37077 Göttingen, Germany; 5 Dermfocus, University of Bern, Bremgartenstrasse 109a, 3001 Bern, Switzerland

## Abstract

The South African Boer goat displays a characteristic white spotting phenotype, in which the pigment is limited to the head. Exploiting the existing phenotype variation within the breed, we mapped the locus causing this white spotting phenotype to chromosome 17 by genome wide association. Subsequent whole genome sequencing identified a 1 Mb copy number variant (CNV) harboring 5 genes including *EDNRA*. The analysis of 358 Boer goats revealed 3 alleles with one, two, and three copies of this CNV. The copy number is correlated with the degree of white spotting in goats. We propose a hypothesis that ectopic overexpression of a mutant EDNRA scavenges EDN3 required for EDNRB signaling and normal melanocyte development and thus likely lead to an absence of melanocytes in the non-pigmented body areas of Boer goats. Our findings demonstrate the value of domestic animals as reservoir of unique mutants and for identifying a precisely defined functional CNV.

Melanocytes are derived from precursor cells termed melanoblasts, which originate from the neural crest. During development melanoblasts migrate over the entire surface of the developing fetus^1^. They have to cross the basal membrane to finally settle in the epidermis as either hair-follicle associated or interfollicular melanocytes. This process of melanocyte migration and proliferation shares similarities with tumor metastasis and is delicately regulated to avoid harmful consequences for the organism^1^. Specifically, melanocytes need a number of stimulating signals from their environment for survival. Melanocyte survival promoting factors include KIT ligand (stem cell factor) and the endothelins (EDN1 – EDN3)^1^,^2^,^3^. EDNs exert a variety of effects and activate two different G-protein coupled receptors, EDNRA and EDNRB^4^,^5^. EDNRB signaling is required for melanocyte development^3^,^6^. Loss of function variants in EDNRB lead to white spotting phenotypes in humans (OMIM 131244)^7^ and animals (OMIA 000375-93934; OMIA 001904-9031)^8^,^9^. In humans, different *EDNRB* variants cause Waardenburg syndrome 4A (OMIM 277580) and Hirschsprung disease (aganglionic megacolon; OMIM 600155)^10^. In horses the EDNRB:p.Ile118Lys variant is responsible for the *frame overo* white spotting pattern (OMIA 000629-9796)^11^,^12^,^13^. *Frame overo* horses are heterozygous carriers of the *EDNRB*^*118Lys*^ allele. Homozygous mutant foals are born without pigmentation and die shortly after birth due to colic caused by defects of the intestinal innervation^11^,^12^,^13^. This so called “lethal white foal syndrome” thus closely resembles Hirschsprung disease in humans with EDNRB defects. A hypopigmentation syndrome with megacolon in sheep is associated with homozygous deletion of the entire *EDNRB* gene (OMIA 001765-9940)^14^.

Coat color is a major phenotypic characteristic of domestic animals. During thousands of years of animal domestication, spontaneous mutants with new coat colors have been highly valued by their breeders and positively selected^15^. In many cases coat color was the primary characteristic of new breeds, which are often maintained as closed populations. Thus modern domestic animals provide a huge reservoir of coat color mutants and represent a unique resource for genetic research^16^. While there are some white-spotting phenotypes in domestic animals that are caused by genetic variants with at least potentially pathogenic potential (e.g. non-synonymous variants in *EDNRB*, *KIT*, *MITF*, *PAX3*)^17^,^18^,^19^,^20^ we also know other examples where non-coding causative variants have subtle regulatory effects on gene expression^21^,^22^,^23^. Mutant animals of the latter category to the best of our knowledge only have an altered coat color, but none of the negative health consequences associated with the coding variants mentioned earlier. To name just one relevant example, the majority of the ~1 billion domestic pigs is lacking epidermal melanocytes due to a complex genomic rearrangement involving the duplication of the *KIT* gene^22^.

The Boer goat breed was created from various South African indigenous goats with the ambition to produce a robust and highly fertile goat for meat production^24^,^25^. The name originates from the Dutch word “boer” which means farmer. The traditional colored Boer goats have a non-pigmented white body and a pigmented red head and neck, short hair, long ears and strong horns^24^. There is still some phenotypic variation in the breed, and occasionally solid-colored offspring is born out of two white-spotted parents^25^,^26^.

The present study aimed to investigate the genetics of the white-spotted phenotype in the Boer goat. Exploiting the remaining intrabreed phenotypic variance, we used a genome-wide association study (GWAS) and whole genome sequencing to identify the causative genetic variant.

## Results

### Depigmentation phenotype classification and pedigree analyses

Since the 1990es Boer goats were imported to Switzerland primarily from Germany and initially also directly from South Africa (www.swiss-boer.ch). Breeders noticed that occasionally solid red colored offspring (=”solid-colored”) or lambs with pigmented spots in the white area of the body (=”spotted”) were born from two Boer goats with pigmented head and completely white body and legs (=“traditional”). We re-investigated phenotype and pedigree data of 236 sampled goats and noticed substantial phenotypic variance (Suppl. Fig. 1). We tentatively grouped the animals into 3 different phenotype classes: Solid-colored, spotted, and traditional Boer goats. The pedigree data were compatible with a dominant inheritance of both white-spotted phenotypes over the solid-colored phenotype.

### Boer coat color is associated to the region of *EDNRA* on chromosome 17

**Figure 1:**
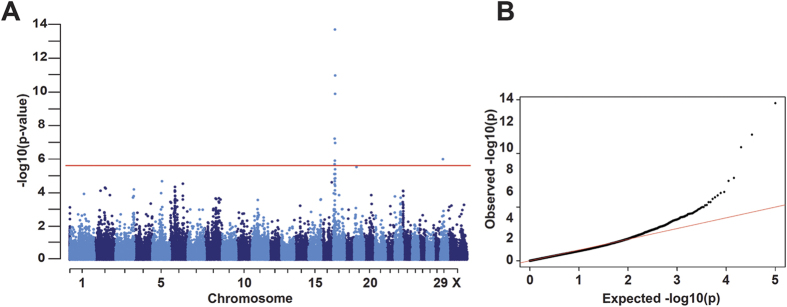
Genome-wide association study for coat color in Boer goats. Comparison of 15 solid-colored versus 172 Boer goats (traditional and spotted). The red line in the Manhattan plot indicates the Bonferroni significance threshold for association (−log10 p = 6.00) showing seven genome-wide significantly associated SNPs on chromosome 17 and one on chromosome 29. (**B**) The quantile-quantile (QQ) plot shows the observed versus the expected log p-values. The straight red line in the QQ plot indicates the distribution of SNP markers under the null hypothesis, and the skew at the right edge indicates that these markers are stronger associated with the trait than it would be expected by chance.

We obtained 50k SNP data from 187 Boer goats consisting of 15 solid-colored, 16 spotted, and 156 traditional animals. We initially performed GWAS comparing the 15 solid-colored versus the 156 traditional Boer goats and obtained a significant association signal on chromosome 17. The association signal further increased when we combined the 16 spotted and 156 traditional Boer goats, indicating that the same locus is involved in the determination of the traditional and spotted phenotypes (Fig. 1). The best associated marker was SNP55406-SCAFFOLD858-1197053 at 10’577’664 bp with a corrected p-value of 2.08x10^−15^ (CHIR_1.0, Suppl. Table 1).

### A 1 Mb CNV is associated with Boer goat coat color

To identify the causative variant we performed whole genome sequencing at 10x coverage of two unrelated Boer goats, one solid-colored and one traditional. We compared the data with genotype data of nine genomes of various Swiss goat breeds that had been sequenced in our laboratory during the course of other ongoing studies. The analysis of SNPs and short indels identified 1029 sequence variants in the 3.5 Mb associated region private to the traditional Boer goat, of which 989 were heterozygous and 40 homozygous calls (Suppl. Table 2). Four of the heterozygous variant positions were located in coding regions of annotated genes: two synonymous SNPs in the nuclear receptor subfamily 3 group C member 2 gene (*NR3C2*) gene, a missense mutation in the transmembrane protein 184C gene (*TMEM184C*; p.Pro77Arg), and a missense mutation in the gene encoding endothelin receptor type A (*EDNRA*; p.Tyr129His). The *EDNRA* variant is predicted to affect a highly conserved transmembrane domain of the receptor (Suppl. Fig. 2).

**Figure 2:**
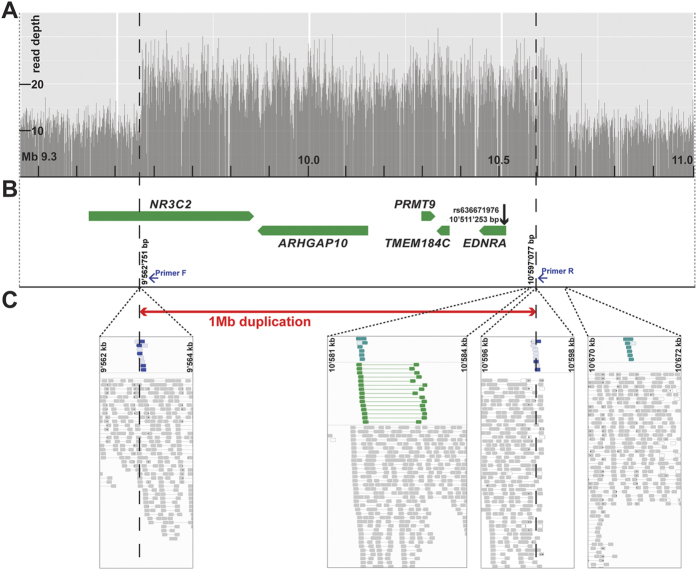
A 1 Mb duplication on chromosome 17 containing EDNRA in Boer goats. (**A**) Sequence coverage plot for a re-sequenced traditional colored Boer goat. Note the twofold increased read depth from 9.5 to 10.6 Mb on chromosome 17. (**B**) Gene content of the duplicated region. The missense SNP in EDNRA is shown. Duplication breakpoints (dashed lines) and inversion were determined by PCR (blue arrows indicate primers). (**C**) Read alignments from whole-genome sequencing of a re-sequenced traditional colored Boer goat. Screenshots from IGV indicate an inverted 1 Mb duplication in blue and turquoise. Reads indicating a tandem-duplication are shown in green. The red arrow shows the PCR confirmed sequence breakpoints of the 1’034’326 bp sized duplication.

**Figure 3:**
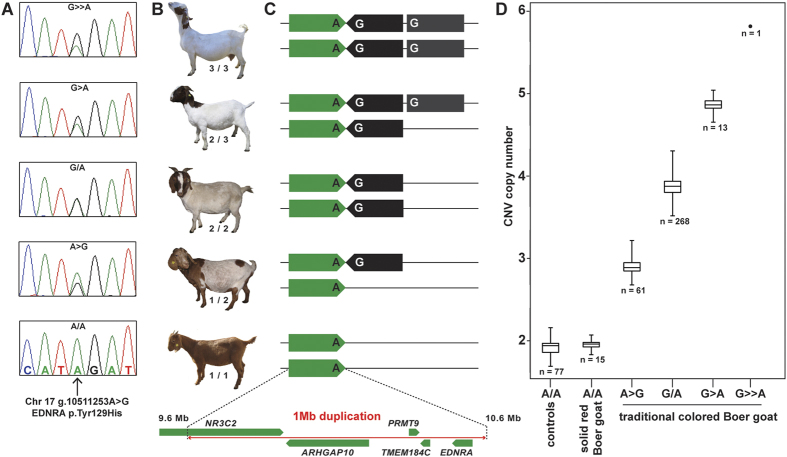
*EDNRA* associated copy number variation in Boer goats with coat color differences. (**A**) Electropherograms of the *EDNRA* c.385A > G missense mutation (rs636671976). The solid-colored Boer goats shown at the bottom are homozygous A/A (wildtype). Note the different relative peak size of two SNP alleles in the four other goats. (**B**) Representative pictures of Boer goats with variable copy numbers of the CNV. (**C**) The green arrow indicates the ancestral genomic segment from position 9’562’751 to 10’597’077 on chromosome 17 containing the wildtype *EDNRA* A-allele (^129^Tyr). The black arrow indicates the duplicated and inverted mutant copy of this segment containing the *EDNRA* G-allele (^129^His). The grey box indicates an additional third copy of the CNV. (**D**) CNV copy numbers for 358 Boer goats and 77 controls analyzed with droplet digital PCR (ddPCR). Note the correlation of increasing white with increasing CNV copy number as illustrated by representative photos in (**B**).

The high proportion of heterozygous variant calls prompted us to analyze the whole genome sequence data for large structural variants in the associated region on chromosome 17. Several read-pairs with unexpected read orientation and insert size and a two-fold increased coverage in the traditional Boer goat indicated the presence of a large genomic duplication (Fig. 2). PCR with primers flanking the breakpoints and subsequent Sanger sequencing of the obtained PCR product confirmed the existence of the duplication. The duplicated segment spanned positions 9,562,751 to 10,597,077 of chromosome 17 and was in a head-to head orientation. It contains a part of the *NR3C2* gene and the complete *ARHGAP10*, *PRMT9*, *TMEM184C*, and *EDNRA* genes (Fig. 3). Thus the sequenced traditional Boer goat had 2 wildtype and 2 mutant copies at the above mentioned non-synonymous variants in the *TMEM184C* and *EDNRA* genes. The non-duplicated allele of the solid-colored Boer goat is representative of the ancestral state, and the duplication present in the sequenced traditional colored Boer goat is derived and contains numerous sequence variants.

Quantitative analysis of the Sanger electropherograms of the *EDNRA* variant was used to genotype the 236 Boer goats of the discovery cohort (Fig. 3). We confirmed and refined the copy number variant (CNV) genotype analysis by digital droplet PCR (ddPCR) in an extended cohort of 358 Boer goats (including the 236 original animals) and 77 controls from 11 different other breeds. The ddPCR revealed the expected copy number of 2 (genotype 1/1) for all solid-colored Boer and control goats. In the remaining 343 Boer goats with the spotted or traditional phenotype, we observed copy numbers between 3 and 6 indicating the presence of at least one rare additional CNV allele with 3 copies (Fig. 3; Table 1). Subjectively, the goats with more copies had less pigmentation (Fig. 3). On average the spotted Boer goats have 3.74 copies and the traditional colored goats 3.88 copies (P = 0.0009286, Pearson's Chi-squared test).

## Discussion

Coat color genetics in domestic animals can provide valuable insights for studying fundamental aspects of gene function. We provide strong genetic evidence that a 1 Mb CNV in the goat genome causes a variable white-spotting phenotype in Boer goats. The GWAS clearly demonstrated that a single locus on chromosome 17 is controlling this trait. Whole genome sequencing of a traditional colored Boer goat did not reveal any small private homozygous non-synonymous variants but the existence of a homozygous 1 Mb duplication harboring 5 genes. The remarkable correlation of CNV copy number with decreasing pigmentation in the studied animals supports the possible causality of the CNV as it would be hard to explain the different phenotype variations, if the underlying causative variant were bi-allelic, such as with e.g. a single nucleotide variant.

None of the five duplicated genes has a known role in pigmentation. *NR3C2* encodes a mineralocorticoid receptor involved in blood pressure regulation (OMIM 177735)^27^. The physiological functions of *ARHGAP10* encoding rho GTPase-activating protein 10, *PRMT9* encoding protein arginine methyltransferase 9, and *TMEM184C* encoding transmembrane protein 184C are largely unknown. *EDNRA* encoding the endothelin A receptor is essential for branchial arch development and not known to be directly involved in pigmentation^3^,^28^,^29^. The EDNRA protein has much higher affinity for EDN1 and EDN2 compared to EDN3 (K_i EDN1_ = 0.018 nM, K_i EDN2_ = 0.058 nM, K_i EDN3_ = 6.35 nM). EDNRB binds all 3 EDNs with similar high affinity (K_i EDN1_ = 0.012, K_i EDN2_ = 0.017 nM, K_i EDN3_ = 0.016 nM)^30^,^31^. Tyr-129 in the second transmembrane domain of EDNRA is very important for the ligand specificity of EDNRA. A recombinantly expressed EDNRA with a targeted Tyr129His mutation showed 100-fold increased affinity for EDN3 (K_i EDN1_ = 0.017 nM, K_i EDN2_ = 0.025 nM, K_i EDN3_ = 0.062 nM)^31^. Similar K_i_ values were obtained for a Tyr129Phe variant. Spontaneous independent *de novo* p.Tyr129Phe mutations were shown to cause dominant mandibulofacial dysostosis with alopecia in humans and a recombinant EDNRA with the Tyr129Phe mutation is no longer able to activate downstream signaling upon stimulation with either EDN1 or EDN3 (OMIM 616367)^32^.

Although our experiment does not allow the discrimination which (or which combination) of the five duplicated genes is functionally responsible for the white-spotting phenotype, the previous knowledge on endothelin signaling suggest a plausible mechanistic hypothesis: Boer goats possess one or several additional copies of a mutant *EDNRA* gene encoding a non-functional receptor with greatly increased affinity for EDN3. We propose that this mutant receptor diminishes the amount of EDN3 in specific body regions to a level, which is insufficient to maintain the EDNRB signal required for melanocyte survival. The even and regular boundaries of the pigmented areas in Boer goats further suggest that the melanoblasts/melanocytes are lost relatively early during fetal development, perhaps already in the neural crest. It was shown before that EDN1/EDNRA signaling is required for the normal formation of neural crest derivatives such as cartilage and melanocytes^33^. If our hypothesis is correct, then the Boer goat *EDNRA* CNV has “accomplished” the remarkable feat of modulating endothelin signaling in a way that exclusively alters pigmentation without any harmful side-effects on health. A functional confirmation of this hypothesis might be obtained by genetically engineering the Boer goat CNV or at least a recombinant construct overexpressing the EDNRA^129P*he*^ into the genome of e.g. zebrafish or mice. If our hypothesis is correct, then such animals should have reduced pigmentation compared to wildtype controls. Recent advances in gene editing technology, e.g. the CRISPR/Cas9 method, should facilitate such experiments in the future.

In conclusion, we have identified a 1 Mb CNV on chromosome 17 as a candidate causative variant for the striking coat color phenotype in Boer goats. Increasing copy number at this CNV may lead to reduced body pigmentation in a dose-dependent manner. We propose a hypothesis that the ectopic expression of mutant EDNRA from the amplified sequence withdraws EDN3 and thus blocks EDNRB signaling, leading to the observed phenotype. The CNV in Boer goats suggests the possibility of a completely novel mechanism underlying a white-spotting phenotype without adverse health consequences.

## Methods

### Animals

We collected EDTA blood samples and hair root samples of 236 Boer goats from Switzerland and scored them for coat color. Blood sampling was done with owner consent and was carried out in accordance with the national guidelines for animal welfare. Blood collection from the jugular vein of goats does not require anesthesia and the study was approved according to the national guidelines for animal welfare by the “Cantonal Committee for Animal Experiments” (Canton of Bern; permit BE77/13). We had 15 solid-colored goats, 30 spotted goats and 191 traditional Boer goats showing a pigmented head and neck together with a completely white body. Genomic DNA was either isolated from EDTA-blood using the Nucleon Bacc2 kit or hair roots using Qiagen’s DNeasy Blood & Tissue Kit according to the manufacturers’ instructions. A second cohort consisted of 9 spotted and 113 traditional colored Boer goats from a single German breeding flock. Finally, DNA samples from a control cohort of 77 goats from eleven different breeds were taken from the biobank of the Institute of Genetics.

### SNP genotyping

Genomic DNA samples of 191 Boer goats were genotyped with the illumina goat SNP50 BeadChip for 53,347 SNP markers^34^. At first we removed all individuals and SNPs with call rates <95%. We also excluded SNPs strongly deviating from Hardy-Weinberg equilibrium (p = 10E-6) and markers with a minor allele frequency of <0.01. After the initial quality control steps, 187 individuals and 50,380 SNPs remained. We performed an allelic genome-wide association study with 172 cases (spotted & traditional) and 15 controls (solid-colored) by analyzing the data with the mixed model from the GenABEL package in the R environment that corrects for the population stratification^35^.

### Whole-genome sequencing

We performed a whole-genome sequencing of two unrelated animals, one solid-colored and one traditional colored Boer goat. In the course of previous projects we re-sequenced the genomes of 9 goats from other breeds as described before^36^,^37^. Briefly, we prepared fragment libraries with 300 bp insert size for each animal and collected ~150 million 2x 100 bp paired-end reads on a HiSeq 2500 instrument (illumina, San Diego, USA), which corresponds to roughly 10x coverage of the individual genomes. The reads were mapped against the goat reference genome assembly (CHIR_1.0) of a Chinese Yunnan black goat^38^ using Bowtie2 v. 2.1.0. The Genome Analysis Tool Kit’s (GATK) Unified Genotyper v. 2.6.4 was used to call SNPs and small indels jointly on all eleven genomes^39^. The snpEff software v. 3.4^40^ together with the CHIR_1.0 annotation was used to predict the functional effects of detected variants. The IGV-viewer software^41^ was used for visual inspection of sequence variants.

### PCR and Sanger sequencing

We designed primers (fwd CACTCAGCCAGCTTAGGACT; rev ACTCAGCTTCGTGGTTACCA) for amplification of a 537 bp PCR product containing a coding SNP on chromosome 17 (rs636671976, Chr17: g.10511253A > G; *EDNRA* p.Tyr129His). The PCR products were subsequently directly sequenced by Sanger sequencing using BigDye Terminator Sequencing Kit 3.1 (Life Technologies) on an ABI 3730 (Life Technologies) after treatment with exonuclease I (New England Biolabs) and rAPid Alkaline Phosphatase (Roche). Sequence data were analyzed with Sequencher 5.1 (GeneCodes).

### Droplet digital PCR

To evaluate the detected genomic duplication on chromosome 17 we designed a droplet digital PCR (ddPCR) assay. The targeted region harbors exon 1 of the *EDNRA* gene (chr 17: 10,511,000-10,511,800 bp on CHIR_1.0). Primers and probes were designed using the Primer3 program (http://bioinfo.ut.ee/primer3-0.4.0/primer3/). An 86 bp amplicon was used (fwd GAGTTCTGTCCCATGGAAAG; rev GTGTCATCAGTGATAACGCT; probe FAM-CTTAGGTTTGTGCTGTAGCT-BHQ1) to detect the *EDNRA* locus. An 86 bp fragment of the *coagulation factor II thrombin* (*F2*) gene (chr 15: 72954500-72955300 bp; CHIR_1.0) served as control amplicon. This genomic region showed equal copy-numbers among the 11 sequenced goat genomes and has no known role in coat color genetics. The sequences of the primers and probe for *F2* were: fwd CCCTTCCTAACCATCTCAGT; rev ATAACGAGAATCATGCAGGG; probe HEX-CAACAGCAAGCCTCGTAC-BHQ1. The ddPCR was performed as described before^42^. Briefly, droplets were generated using the QX200 Droplet Generator (Bio-Rad) and analyzed with the QX200 Droplet Reader (Bio-Rad). Briefly, 50–150 ng of genomic DNA were digested using 10 units of *Acc*I restriction enzyme. The restriction digest was prepared in 1x ddPCR ddPCR Supermix for Probes (Bio-Rad), after incubation at 37 °C for 60 min, primers (900 nM each) and probes (250 nM each) were added directly to the mix and droplets were generated using the QX200 Droplet Generator (Bio-Rad). The reactions were amplified with the following temperature profile: initial denaturation for 10 min at 95 °C; 50 cycles of: 30 sec at 95 °C, 60 sec at 56 °C; final droplet stabilization: 10 min at 98 °C. Droplets were analyzed with the QX200 Droplet Reader (Bio-Rad).

## Additional Information

**Accession codes:** Genome sequencing data were deposited in the Sequence Read Archive (SRA) (http://www.ncbi.nlm.nih.gov/sra) under study accession no.SRP069284.

**How to cite this article**: Menzi, F. *et al*. Genomic amplification of the caprine *EDNRA* locus might lead to a dose dependent loss of pigmentation. *Sci. Rep.* **6**, 28438; doi: 10.1038/srep28438 (2016).

## Supplementary Material

Supplementary Information

## Figures and Tables

**Table 1:** Coat color phenotypes and CNV genotypes in Boer goats.

Phenotype	Number of animals	Copy number (CNV genotype)				
		**2 (1/1)**	**3 (1/2)**	**4 (2/2)**	**5 (2/3)**	**6 (3/3)**
Solid-colored	15	15				
Spotted	39		14	21	4	
Traditional	304		47	247	9	1
						
Total	358	15	61	268	13	1
