# Supplementary material for: Genomic amplification of the caprine EDNRA locus might lead to a dose dependent loss of pigmentation
Source: Sci Rep. 2016 Jun 22;6:28438. doi: 10.1038/srep28438 (PMC4916431; doi:10.1038/srep28438)
Supplement: Supplementary Information [file srep28438-s1.pdf]

Title: Genomic amplification of the caprine *EDNRA* locus might lead to a dose dependent loss of pigmentation

Authors: Fiona Menzi, Irene Keller, Irene Reber, Julia Beck, Bertram Brenig, Ekkehard Schütz, Tosso Leeb, Cord Drögemüller

Supplementary information

PDF file

1. Additional_Information.PDF
   Supplementary Figures 1-2 and Supplementary Tables 1-2

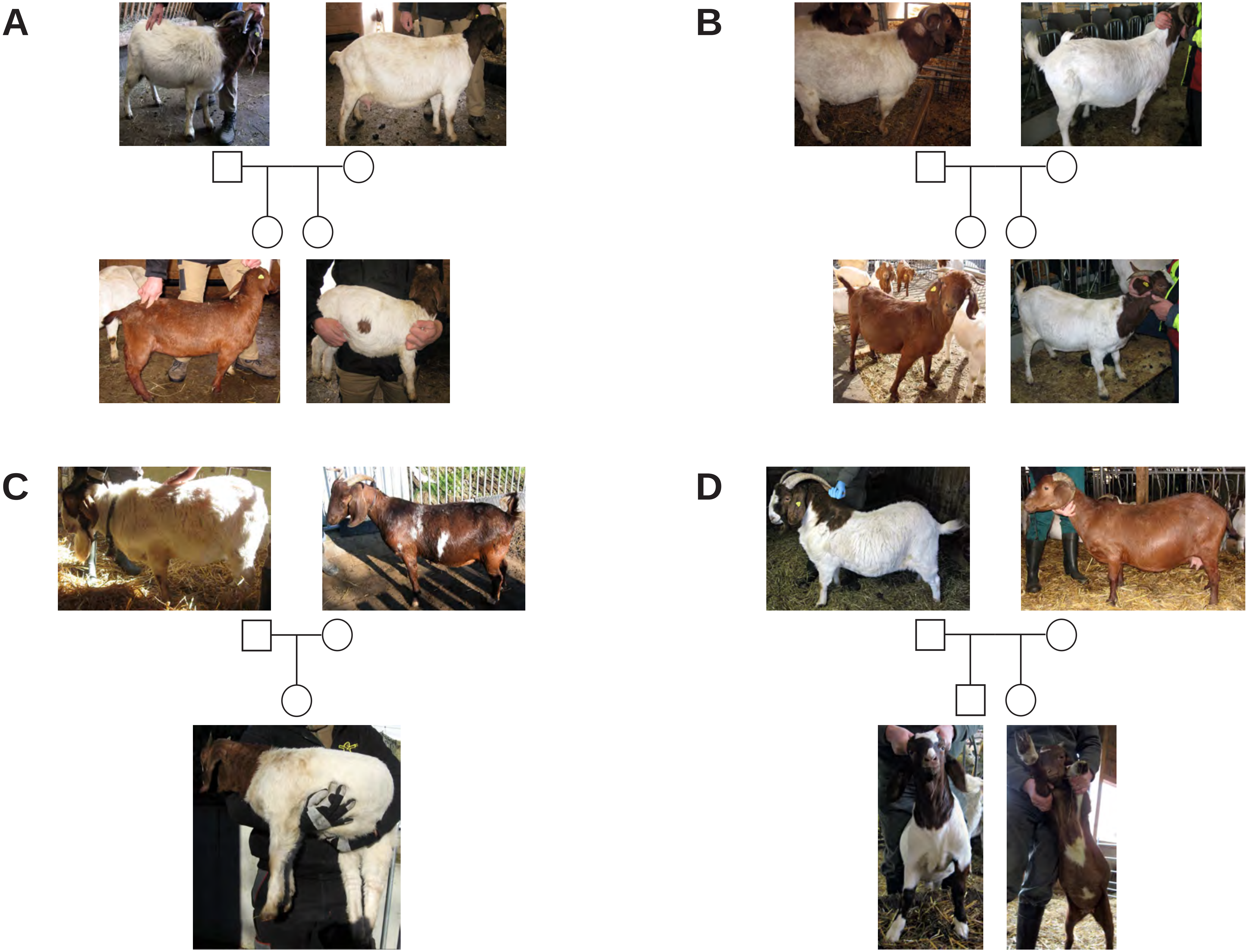

Suppl. Fig. 1 **Examples of coat color inheritance in Boer goats.**
(A+B) Note the occurrence of solid red colored lambs, lambs with red spots in the white area of the body, and traditional colored lambs born from two traditional colored parents. (C+D) Note the occurrence of lambs with red spots in the white area of the body, and traditional colored lambs born from matings of solid red colored and traditional colored parents.

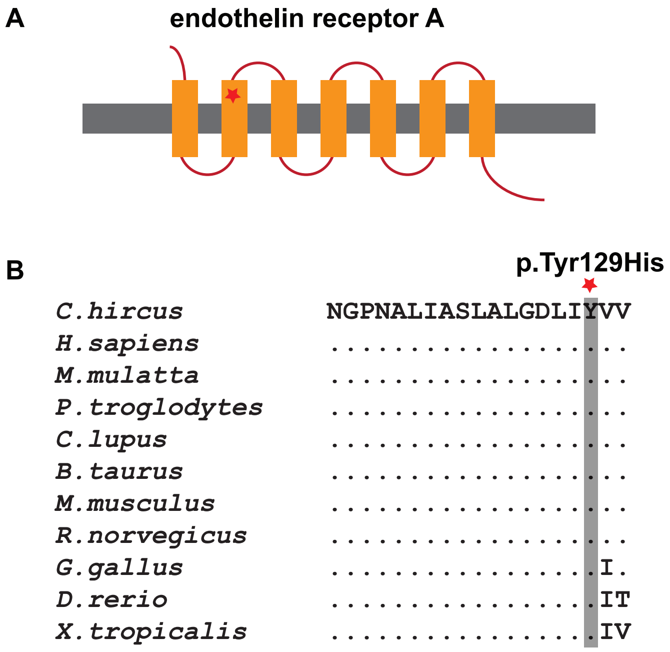

Suppl. Fig. 2 **Multispecies protein sequence alignment of EDNRA.**
(A) The protein consists of seven transmembrane domains (displayed in orange). The p.Tyr129His mutation is indicated by a red star. (B) Note the high conservation of the tyrosine at position 129 in all EDNRA homologs..

Suppl. Tab. 1 Ten best associated SNP markers for coat color differences in Boer goats.
Eight SNPs (of which seven are on chromosome 17) showed a genome-wide significant association (threshold indicated by a red line).

rs number	SNP	chr	position	A1	A2	N	effB	se_effB	chi2.1df	P1df	Pc1df
rs268287000	snp55406-scaffold858-1197053	17	10'577'664	G	A	187	-0.4691956	0.05669374	68.49165	1.274163E-16	2.079603E-15
rs268264927	snp32749-scaffold379-151202	17	11'926'018	A	G	187	-0.3477676	0.04729820	54.06162	1.942992E-13	1.775484E-12
rs268286979	snp55385-scaffold858-292727	17	11'481'990	G	A	187	-0.3308322	0.04760075	48.30453	3.649075E-12	2.645489E-11
rs268245303	snp12503-scaffold1479-728936	17	8'698'303	T	C	187	-0.2188954	0.03735236	34.34292	4.620780E-09	1.910617E-08
rs268286973	snp55379-scaffold858-66034	17	11'708'683	A	G	183	-0.2105514	0.03665209	33.00037	9.214127E-09	3.609544E-08
rs268286576	snp54969-scaffold840-1593888	29	16'642'349	C	T	187	-0.4774763	0.09023900	27.99722	1.214896E-07	3.891218E-07
rs268287011	snp55417-scaffold858-1731348	17	10'043'369	C	T	187	-0.3259177	0.06211019	27.53532	1.542518E-07	4.849542E-07
rs268245299	snp12499-scaffold1479-571823	17	8'541'190	A	G	187	-0.2357979	0.04585936	26.43771	2.721753E-07	8.187017E-07
rs268234705	snp1616-scaffold1046-857683	19	15'060'421	A	G	185	-0.3323220	0.06569107	25.59208	4.217672E-07	1.226198E-06
rs268286982	snp55388-scaffold858-438489	17	11'336'228	A	G	187	-0.1665744	0.03339274	24.88356	6.089965E-07	1.720719E-06

Suppl. Tab. 2 List of 1029 sequence variants in the associated region on goat chromosome 17.

Chr 17 position (CHIR_1.0 assembly)	Reference allele	Alternative allele	Traditional Boer goat (BUR029)	Solid-colored Boer goat (BUR009)	Grison Striped Goat (BST001)	Tessin Grey Goat (CAG038)	Chamois goat (GFG024)	Chamois goat (GFG034)	Coppernecked goat (KHZ098)	Saanen goat (SAN041)	Saanen goat (SAN049)	Saanen goat (SAN075)	Saanen goat (SAN081)	SNPeff output
9'404'639	G	A	1/1	0/0	0/0	0/0	0/0	0/0	0/0	0/0	0/0	0/0	0/0	INTERGENIC(MODIFIER)
9'450'519	C	T	1/1	0/0	0/0	0/0	0/0	0/0	0/0	0/0	0/0	0/0	0/0	INTRON(MODIFIER|||NR3C2||rna18471|2)
9'450'609	C	T	1/1	0/0	0/0	0/0	0/0	0/0	0/0	0/0	0/0	0/0	0/0	INTRON(MODIFIER|||NR3C2||rna18471|2)
9'468'871	C	G	1/1	0/0	0/0	0/0	0/0	0/0	0/0	0/0	0/0	0/0	0/0	INTRON(MODIFIER|||NR3C2||rna18471|2)
9'472'154	T	C	1/1	0/0	0/0	0/0	0/0	0/0	0/0	0/0	0/0	0/0	0/0	INTRON(MODIFIER|||NR3C2||rna18471|2)
9'472'995	G	A	1/1	0/0	0/0	0/0	0/0	0/0	0/0	0/0	0/0	0/0	0/0	INTRON(MODIFIER|||NR3C2||rna18471|2)
9'479'096	C	T	1/1	0/0	0/0	0/0	0/0	0/0	0/0	0/0	0/0	0/0	0/0	INTRON(MODIFIER|||NR3C2||rna18471|2)
9'479'521	C	A	1/1	0/0	0/0	0/0	0/0	0/0	0/0	0/0	0/0	0/0	0/0	INTRON(MODIFIER|||NR3C2||rna18471|2)
9'483'410	C	T	1/1	0/0	0/0	0/0	0/0	0/0	0/0	0/0	0/0	0/0	0/0	INTRON(MODIFIER|||NR3C2||rna18471|2)
9'487'569	T	A	1/1	0/0	0/0	0/0	0/0	0/0	0/0	0/0	0/0	0/0	0/0	INTRON(MODIFIER|||NR3C2||rna18471|2)
9'492'144	G	T	1/1	0/0	0/0	0/0	0/0	0/0	0/0	0/0	0/0	0/0	0/0	INTRON(MODIFIER|||NR3C2||rna18471|2)
9'503'050	G	A	1/1	0/0	0/0	0/0	0/0	0/0	0/0	0/0	0/0	0/0	0/0	INTRON(MODIFIER|||NR3C2||rna18471|2)
9'503'897	A	G	1/1	0/0	0/0	0/0	0/0	0/0	0/0	0/0	0/0	0/0	0/0	INTRON(MODIFIER|||NR3C2||rna18471|2)
9'519'256	T	A	1/1	0/0	0/0	0/0	0/0	0/0	0/0	0/0	0/0	0/0	0/0	INTRON(MODIFIER|||NR3C2||rna18471|2)
9'522'534	C	T	1/1	0/0	0/0	0/0	0/0	0/0	0/0	0/0	0/0	0/0	0/0	INTRON(MODIFIER|||NR3C2||rna18471|2)
9'532'392	T	A	1/1	0/0	0/0	0/0	0/0	0/0	0/0	0/0	0/0	0/0	0/0	INTRON(MODIFIER|||NR3C2||rna18471|2)
9'532'515	TCAACAA	T	1/1	0/0	0/0	0/0	0/0	0/0	0/0	0/0	0/0	0/0	0/0	INTRON(MODIFIER|||NR3C2||rna18471|2)
9'554'773	A	T	1/1	0/0	0/0	0/0	0/0	0/0	0/0	0/0	0/0	0/0	0/0	INTRON(MODIFIER|||NR3C2||rna18471|2)
9'559'355	G	A	1/1	0/0	0/0	0/0	0/0	0/0	0/0	0/0	0/0	0/0	0/0	INTRON(MODIFIER|||NR3C2||rna18471|2)
9'562'151	A	ATG	1/1	0/0	0/0	0/0	0/0	0/0	0/0	0/0	0/0	0/0	0/0	INTRON(MODIFIER|||NR3C2||rna18471|2)
9'567'619	G	A	0/1	0/0	0/0	0/0	0/0	0/0	0/0	0/0	0/0	0/0	0/0	INTRON(MODIFIER|||NR3C2||rna18471|2)
9'568'528	C	T	0/1	0/0	0/0	0/0	0/0	0/0	0/0	0/0	0/0	0/0	0/0	INTRON(MODIFIER|||NR3C2||rna18471|2)
9'569'538	C	A	0/1	0/0	0/0	0/0	0/0	0/0	0/0	./.	0/0	0/0	0/0	INTRON(MODIFIER|||NR3C2||rna18471|2)
9'570'167	A	G	0/1	0/0	0/0	0/0	0/0	0/0	0/0	0/0	0/0	0/0	0/0	INTRON(MODIFIER|||NR3C2||rna18471|2)
9'570'215	T	C	0/1	0/0	0/0	0/0	0/0	0/0	0/0	0/0	0/0	0/0	0/0	INTRON(MODIFIER|||NR3C2||rna18471|2)
9'573'914	C	T	0/1	0/0	0/0	0/0	0/0	0/0	0/0	0/0	0/0	0/0	0/0	INTRON(MODIFIER|||NR3C2||rna18471|2)
9'574'012	G	T	0/1	0/0	0/0	0/0	0/0	0/0	0/0	0/0	0/0	0/0	0/0	INTRON(MODIFIER|||NR3C2||rna18471|2)
9'574'333	A	T	0/1	0/0	0/0	0/0	0/0	0/0	0/0	0/0	0/0	0/0	0/0	INTRON(MODIFIER|||NR3C2||rna18471|2)
9'574'355	G	A	0/1	0/0	0/0	0/0	0/0	0/0	0/0	0/0	0/0	0/0	0/0	INTRON(MODIFIER|||NR3C2||rna18471|2)
9'574'890	TG	T	0/1	0/0	0/0	0/0	0/0	0/0	0/0	0/0	0/0	0/0	0/0	INTRON(MODIFIER|||NR3C2||rna18471|2)
9'575'255	T	C	0/1	0/0	0/0	0/0	0/0	0/0	0/0	0/0	0/0	0/0	0/0	INTRON(MODIFIER|||NR3C2||rna18471|2)
9'575'338	A	G	0/1	0/0	0/0	0/0	0/0	0/0	0/0	0/0	0/0	0/0	0/0	INTRON(MODIFIER|||NR3C2||rna18471|2)
9'578'649	A	G	0/1	0/0	0/0	0/0	0/0	0/0	0/0	0/0	0/0	0/0	0/0	INTRON(MODIFIER|||NR3C2||rna18471|2)
9'578'738	T	A	0/1	0/0	0/0	0/0	0/0	0/0	0/0	0/0	0/0	0/0	0/0	INTRON(MODIFIER|||NR3C2||rna18471|2)
9'580'693	T	C	0/1	0/0	0/0	0/0	0/0	0/0	0/0	0/0	0/0	0/0	0/0	INTRON(MODIFIER|||NR3C2||rna18471|2)
9'582'213	C	A	0/1	0/0	0/0	0/0	0/0	0/0	0/0	0/0	0/0	0/0	0/0	INTRON(MODIFIER|||NR3C2||rna18471|2)
9'583'342	A	G	0/1	0/0	0/0	0/0	0/0	0/0	0/0	0/0	0/0	0/0	0/0	INTRON(MODIFIER|||NR3C2||rna18471|2)
9'584'343	T	G	0/1	0/0	0/0	0/0	0/0	0/0	0/0	0/0	0/0	0/0	0/0	INTRON(MODIFIER|||NR3C2||rna18471|2)
9'584'784	A	C	0/1	0/0	0/0	0/0	0/0	0/0	0/0	0/0	0/0	0/0	0/0	INTRON(MODIFIER|||NR3C2||rna18471|2)
9'584'906	G	A	0/1	0/0	0/0	0/0	0/0	0/0	0/0	0/0	0/0	0/0	0/0	INTRON(MODIFIER|||NR3C2||rna18471|2)
9'585'440	G	A	0/1	0/0	0/0	0/0	0/0	0/0	0/0	0/0	0/0	0/0	0/0	INTRON(MODIFIER|||NR3C2||rna18471|2)
9'589'323	G	C	0/1	0/0	0/0	0/0	0/0	0/0	0/0	0/0	0/0	0/0	0/0	INTRON(MODIFIER|||NR3C2||rna18471|2)
9'591'500	G	A	0/1	0/0	0/0	0/0	0/0	0/0	0/0	0/0	0/0	0/0	0/0	INTRON(MODIFIER|||NR3C2||rna18471|2)
9'591'536	C	T	0/1	0/0	0/0	0/0	0/0	0/0	0/0	0/0	0/0	0/0	0/0	INTRON(MODIFIER|||NR3C2||rna18471|2)
9'592'991	A	T	0/1	0/0	0/0	0/0	0/0	0/0	0/0	0/0	0/0	0/0	0/0	INTRON(MODIFIER|||NR3C2||rna18471|2)
9'596'453	T	A	0/1	0/0	0/0	0/0	0/0	0/0	0/0	0/0	0/0	0/0	0/0	INTRON(MODIFIER|||NR3C2||rna18471|2)
9'597'791	C	T	0/1	0/0	0/0	0/0	0/0	0/0	0/0	0/0	0/0	0/0	0/0	INTRON(MODIFIER|||NR3C2||rna18471|2)
9'598'848	G	A	0/1	0/0	0/0	0/0	0/0	0/0	0/0	0/0	0/0	0/0	0/0	INTRON(MODIFIER|||NR3C2||rna18471|2)
9'600'318	C	T	0/1	0/0	0/0	0/0	0/0	0/0	0/0	0/0	0/0	0/0	0/0	INTRON(MODIFIER|||NR3C2||rna18471|2)
9'631'192	G	A	0/1	0/0	0/0	0/0	0/0	0/0	0/0	0/0	0/0	0/0	0/0	INTRON(MODIFIER|||NR3C2||rna18471|2)
9'631'371	A	G	0/1	0/0	0/0	0/0	0/0	0/0	0/0	0/0	0/0	0/0	0/0	INTRON(MODIFIER|||NR3C2||rna18471|2)
9'633'761	TTA	T	0/1	0/0	0/0	0/0	0/0	0/0	0/0	0/0	0/0	0/0	0/0	INTRON(MODIFIER|||NR3C2||rna18471|2)
9'636'193	G	A	0/1	0/0	0/0	0/0	0/0	0/0	0/0	0/0	0/0	0/0	0/0	INTRON(MODIFIER|||NR3C2||rna18471|2)
9'636'356	C	T	0/1	0/0	0/0	0/0	0/0	0/0	0/0	0/0	0/0	0/0	0/0	INTRON(MODIFIER|||NR3C2||rna18471|2)
9'636'628	A	G	0/1	0/0	0/0	0/0	0/0	0/0	0/0	0/0	0/0	0/0	0/0	INTRON(MODIFIER|||NR3C2||rna18471|2)
9'640'379	C	T	0/1	0/0	0/0	0/0	0/0	0/0	0/0	0/0	0/0	0/0	0/0	INTRON(MODIFIER|||NR3C2||rna18471|2)
9'652'433	C	T	0/1	0/0	0/0	0/0	0/0	0/0	0/0	0/0	0/0	0/0	0/0	INTRON(MODIFIER|||NR3C2||rna18471|3)
9'653'045	G	A	0/1	0/0	0/0	0/0	0/0	0/0	0/0	0/0	0/0	0/0	0/0	INTRON(MODIFIER|||NR3C2||rna18471|3)
9'657'575	C	T	0/1	0/0	0/0	0/0	0/0	0/0	0/0	0/0	0/0	0/0	0/0	INTRON(MODIFIER|||NR3C2||rna18471|3)
9'662'095	A	G	0/1	0/0	0/0	0/0	0/0	0/0	0/0	0/0	0/0	0/0	0/0	INTRON(MODIFIER|||NR3C2||rna18471|3)
9'683'802	A	G	0/1	0/0	0/0	0/0	0/0	0/0	0/0	0/0	0/0	0/0	0/0	INTRON(MODIFIER|||NR3C2||rna18471|3)
9'696'344	GA	G	0/1	0/0	0/0	0/0	0/0	0/0	0/0	0/0	0/0	0/0	0/0	INTRON(MODIFIER|||NR3C2||rna18471|3)
9'699'476	G	A	0/1	0/0	0/0	0/0	0/0	0/0	0/0	0/0	0/0	0/0	0/0	INTRON(MODIFIER|||NR3C2||rna18471|3)
9'700'420	C	G	0/1	0/0	0/0	0/0	0/0	0/0	0/0	0/0	0/0	0/0	0/0	INTRON(MODIFIER|||NR3C2||rna18471|3)
9'702'704	A	G	0/1	0/0	0/0	0/0	0/0	0/0	0/0	0/0	0/0	0/0	0/0	INTRON(MODIFIER|||NR3C2||rna18471|3)
9'703'860	T	G	0/1	0/0	0/0	0/0	0/0	0/0	0/0	0/0	0/0	0/0	0/0	INTRON(MODIFIER|||NR3C2||rna18471|3)
9'704'896	G	A	0/1	0/0	0/0	0/0	0/0	0/0	0/0	0/0	0/0	0/0	0/0	INTRON(MODIFIER|||NR3C2||rna18471|3)
9'704'991	C	T	0/1	0/0	0/0	0/0	0/0	0/0	0/0	0/0	0/0	0/0	0/0	INTRON(MODIFIER|||NR3C2||rna18471|3)
9'705'342	C	T	0/1	0/0	0/0	0/0	0/0	0/0	0/0	0/0	0/0	0/0	0/0	INTRON(MODIFIER|||NR3C2||rna18471|3)
9'707'934	G	T	1/1	0/0	0/0	0/0	0/0	0/0	0/0	0/0	0/0	0/0	0/0	INTRON(MODIFIER|||NR3C2||rna18471|3)
9'708'283	T	TAGATACA	0/1	0/0	0/0	0/0	0/0	0/0	0/0	0/0	0/0	0/0	0/0	INTRON(MODIFIER|||NR3C2||rna18471|3)
9'708'384	C	G	0/1	0/0	0/0	0/0	0/0	0/0	0/0	0/0	0/0	0/0	0/0	INTRON(MODIFIER|||NR3C2||rna18471|3)
9'708'958	T	C	0/1	0/0	0/0	0/0	0/0	0/0	0/0	0/0	0/0	0/0	0/0	INTRON(MODIFIER|||NR3C2||rna18471|3)
9'709'425	C	G	0/1	0/0	0/0	0/0	0/0	0/0	0/0	0/0	0/0	0/0	0/0	INTRON(MODIFIER|||NR3C2||rna18471|3)
9'709'645	A	G	0/1	0/0	0/0	0/0	0/0	0/0	0/0	0/0	0/0	0/0	0/0	INTRON(MODIFIER|||NR3C2||rna18471|3)
9'710'100	G	A	0/1	0/0	0/0	0/0	0/0	0/0	0/0	0/0	0/0	0/0	0/0	INTRON(MODIFIER|||NR3C2||rna18471|3)
9'710'588	T	C	0/1	0/0	0/0	0/0	0/0	0/0	0/0	0/0	0/0	0/0	0/0	INTRON(MODIFIER|||NR3C2||rna18471|3)
9'711'738	CGACTG	C	0/1	0/0	0/0	0/0	0/0	0/0	0/0	0/0	0/0	0/0	0/0	INTRON(MODIFIER|||NR3C2||rna18471|3)
9'711'933	C	G	0/1	0/0	0/0	0/0	0/0	0/0	0/0	0/0	0/0	0/0	0/0	INTRON(MODIFIER|||NR3C2||rna18471|3)
9'711'979	A	G	0/1	0/0	0/0	0/0	./.	0/0	0/0	0/0	0/0	0/0	0/0	INTRON(MODIFIER|||NR3C2||rna18471|3)
9'712'123	T	C	0/1	0/0	0/0	0/0	0/0	0/0	0/0	0/0	0/0	0/0	0/0	INTRON(MODIFIER|||NR3C2||rna18471|3)
9'712'210	G	T	0/1	0/0	0/0	0/0	0/0	0/0	0/0	0/0	0/0	0/0	0/0	INTRON(MODIFIER|||NR3C2||rna18471|3)
9'712'232	G	A	0/1	0/0	0/0	0/0	0/0	0/0	0/0	0/0	0/0	0/0	0/0	INTRON(MODIFIER|||NR3C2||rna18471|3)
9'712'318	G	A	0/1	0/0	0/0	0/0	0/0	0/0	0/0	0/0	0/0	0/0	0/0	INTRON(MODIFIER|||NR3C2||rna18471|3)
9'712'495	G	A	0/1	0/0	0/0	0/0	0/0	0/0	0/0	0/0	0/0	0/0	0/0	INTRON(MODIFIER|||NR3C2||rna18471|3)
9'712'827	G	C	0/1	0/0	0/0	0/0	0/0	0/0	0/0	0/0	0/0	0/0	0/0	INTRON(MODIFIER|||NR3C2||rna18471|3)
9'713'067	G	A	0/1	0/0	0/0	0/0	0/0	0/0	0/0	0/0	0/0	0/0	0/0	INTRON(MODIFIER|||NR3C2||rna18471|3)
9'713'624	A	G	0/1	0/0	0/0	0/0	0/0	0/0	0/0	0/0	0/0	0/0	0/0	INTRON(MODIFIER|||NR3C2||rna18471|3)
9'714'261	A	G	0/1	0/0	0/0	0/0	0/0	0/0	0/0	0/0	0/0	0/0	0/0	INTRON(MODIFIER|||NR3C2||rna18471|3)
9'714'553	T	G	0/1	0/0	0/0	0/0	0/0	0/0	0/0	0/0	0/0	0/0	0/0	INTRON(MODIFIER|||NR3C2||rna18471|3)
9'715'376	C	T	0/1	0/0	0/0	0/0	0/0	0/0	0/0	0/0	0/0	0/0	0/0	INTRON(MODIFIER|||NR3C2||rna18471|3)
9'717'373	C	G	0/1	0/0	0/0	0/0	0/0	0/0	0/0	0/0	0/0	0/0	0/0	INTRON(MODIFIER|||NR3C2||rna18471|3)
9'717'427	A	G	0/1	0/0	0/0	0/0	0/0	0/0	0/0	0/0	0/0	0/0	0/0	INTRON(MODIFIER|||NR3C2||rna18471|3)
9'717'569	A	G	0/1	0/0	0/0	0/0	0/0	0/0	0/0	0/0	0/0	0/0	0/0	INTRON(MODIFIER|||NR3C2||rna18471|3)
9'717'589	G	T	0/1	0/0	0/0	0/0	0/0	0/0	0/0	0/0	0/0	0/0	0/0	INTRON(MODIFIER|||NR3C2||rna18471|3)
9'718'434	T	A	0/1	0/0	0/0	0/0	0/0	0/0	0/0	0/0	0/0	0/0	0/0	INTRON(MODIFIER|||NR3C2||rna18471|3)
9'718'501	G	A	0/1	0/0	0/0	0/0	0/0	0/0	0/0	0/0	0/0	0/0	0/0	INTRON(MODIFIER|||NR3C2||rna18471|3)
9'718'539	T	C	0/1	0/0	0/0	0/0	0/0	0/0	0/0	0/0	0/0	0/0	0/0	INTRON(MODIFIER|||NR3C2||rna18471|3)
9'718'640	A	G	0/1	0/0	0/0	0/0	0/0	0/0	0/0	0/0	0/0	0/0	0/0	INTRON(MODIFIER|||NR3C2||rna18471|3)
9'718'678	T	G	0/1	0/0	0/0	0/0	0/0	0/0	0/0	0/0	0/0	0/0	0/0	INTRON(MODIFIER|||NR3C2||rna18471|3)
9'720'147	T	TTTTG	0/1	0/0	0/0	0/0	0/0	0/0	0/0	0/0	0/0	0/0	0/0	INTRON(MODIFIER|||NR3C2||rna18471|3)
9'720'412	C	G	0/1	0/0	0/0	0/0	0/0	0/0	0/0	0/0	0/0	0/0	0/0	INTRON(MODIFIER|||NR3C2||rna18471|3)
9'720'558	G	A	0/1	0/0	0/0	0/0	0/0	0/0	0/0	0/0	0/0	0/0	0/0	INTRON(MODIFIER|||NR3C2||rna18471|3)
9'720'676	C	T	0/1	0/0	0/0	0/0	0/0	0/0	0/0	0/0	0/0	0/0	0/0	INTRON(MODIFIER|||NR3C2||rna18471|3)
9'720'849	A	G	0/1	0/0	0/0	0/0	0/0	0/0	0/0	0/0	0/0	0/0	0/0	INTRON(MODIFIER|||NR3C2||rna18471|3)
9'721'733	G	A	0/1	0/0	0/0	0/0	0/0	0/0	0/0	0/0	0/0	0/0	0/0	INTRON(MODIFIER|||NR3C2||rna18471|3)
9'721'792	C	T	0/1	0/0	0/0	0/0	0/0	0/0	0/0	0/0	0/0	0/0	0/0	INTRON(MODIFIER|||NR3C2||rna18471|3)
9'721'817	AT	A	0/1	0/0	0/0	0/0	0/0	0/0	0/0	0/0	0/0	0/0	0/0	INTRON(MODIFIER|||NR3C2||rna18471|3)
9'722'021	T	A	0/1	0/0	0/0	0/0	0/0	0/0	0/0	0/0	0/0	0/0	0/0	INTRON(MODIFIER|||NR3C2||rna18471|3)
9'724'712	G	T	0/1	0/0	0/0	0/0	0/0	0/0	0/0	0/0	0/0	0/0	0/0	INTRON(MODIFIER|||NR3C2||rna18471|3)
9'725'465	G	T	0/1	0/0	0/0	0/0	0/0	0/0	0/0	0/0	0/0	0/0	0/0	INTRON(MODIFIER|||NR3C2||rna18471|3)
9'725'890	G	A	0/1	0/0	0/0	0/0	0/0	0/0	0/0	0/0	0/0	0/0	0/0	SYNONYMOUS_CODING(LOW|SILENT|gcG/gcA|A642|NR3C2||rna18471|4)
9'726'123	G	A	0/1	0/0	0/0	0/0	0/0	0/0	0/0	0/0	0/0	0/0	0/0	INTRON(MODIFIER|||NR3C2||rna18471|4)
9'726'142	A	G	0/1	0/0	0/0	0/0	0/0	0/0	0/0	0/0	0/0	0/0	0/0	INTRON(MODIFIER|||NR3C2||rna18471|4)
9'726'771	G	A	0/1	0/0	0/0	0/0	0/0	0/0	0/0	0/0	0/0	0/0	0/0	INTRON(MODIFIER|||NR3C2||rna18471|4)
9'727'015	T	C	0/1	0/0	0/0	0/0	0/0	0/0	0/0	0/0	0/0	0/0	0/0	INTRON(MODIFIER|||NR3C2||rna18471|4)
9'727'064	T	TA	0/1	0/0	0/0	0/0	0/0	0/0	0/0	0/0	0/0	0/0	0/0	INTRON(MODIFIER|||NR3C2||rna18471|4)
9'727'885	A	G	0/1	0/0	0/0	0/0	0/0	0/0	0/0	0/0	0/0	0/0	0/0	INTRON(MODIFIER|||NR3C2||rna18471|4)
9'728'427	C	G	0/1	0/0	0/0	0/0	0/0	0/0	0/0	0/0	0/0	0/0	0/0	INTRON(MODIFIER|||NR3C2||rna18471|4)
9'729'066	G	A	0/1	0/0	0/0	0/0	0/0	0/0	0/0	0/0	0/0	0/0	0/0	INTRON(MODIFIER|||NR3C2||rna18471|4)
9'731'093	G	T	0/1	0/0	0/0	0/0	0/0	0/0	0/0	0/0	0/0	0/0	0/0	INTRON(MODIFIER|||NR3C2||rna18471|4)
9'736'328	C	T	0/1	0/0	0/0	0/0	0/0	0/0	0/0	0/0	0/0	0/0	0/0	INTRON(MODIFIER|||NR3C2||rna18471|4)
9'741'783	C	T	0/1	0/0	0/0	0/0	0/0	0/0	0/0	0/0	0/0	0/0	0/0	INTRON(MODIFIER|||NR3C2||rna18471|4)
9'743'752	A	G	0/1	0/0	0/0	0/0	0/0	0/0	0/0	0/0	0/0	0/0	0/0	INTRON(MODIFIER|||NR3C2||rna18471|4)
9'746'697	A	C	1/1	0/0	0/0	0/0	0/0	0/0	0/0	0/0	0/0	0/0	0/0	INTRON(MODIFIER|||NR3C2||rna18471|4)
9'747'524	G	A	0/1	0/0	0/0	0/0	0/0	0/0	0/0	0/0	0/0	0/0	0/0	INTRON(MODIFIER|||NR3C2||rna18471|4)
9'747'838	A	G	0/1	0/0	0/0	0/0	0/0	0/0	0/0	0/0	0/0	0/0	0/0	INTRON(MODIFIER|||NR3C2||rna18471|4)
9'752'096	A	C	1/1	0/0	0/0	0/0	0/0	0/0	0/0	0/0	0/0	0/0	0/0	INTRON(MODIFIER|||NR3C2||rna18471|4)
9'753'838	T	A	0/1	0/0	0/0	0/0	0/0	0/0	0/0	0/0	0/0	0/0	0/0	INTRON(MODIFIER|||NR3C2||rna18471|4)
9'754'080	T	C	0/1	0/0	0/0	0/0	0/0	0/0	0/0	0/0	0/0	0/0	0/0	INTRON(MODIFIER|||NR3C2||rna18471|4)
9'754'132	T	C	0/1	0/0	0/0	0/0	0/0	0/0	0/0	0/0	0/0	0/0	0/0	INTRON(MODIFIER|||NR3C2||rna18471|4)
9'754'188	T	A	0/1	0/0	0/0	0/0	0/0	0/0	0/0	0/0	0/0	0/0	0/0	INTRON(MODIFIER|||NR3C2||rna18471|4)
9'754'215	T	C	0/1	0/0	0/0	0/0	0/0	0/0	0/0	0/0	0/0	0/0	0/0	INTRON(MODIFIER|||NR3C2||rna18471|4)
9'754'244	T	C	0/1	0/0	0/0	0/0	0/0	0/0	0/0	0/0	0/0	0/0	0/0	INTRON(MODIFIER|||NR3C2||rna18471|4)
9'754'308	A	G	0/1	0/0	0/0	0/0	0/0	0/0	0/0	0/0	0/0	0/0	0/0	INTRON(MODIFIER|||NR3C2||rna18471|4)
9'754'344	G	A	0/1	0/0	0/0	0/0	0/0	0/0	0/0	0/0	0/0	0/0	0/0	INTRON(MODIFIER|||NR3C2||rna18471|4)
9'754'378	G	T	0/1	0/0	0/0	0/0	0/0	0/0	0/0	0/0	0/0	0/0	0/0	INTRON(MODIFIER|||NR3C2||rna18471|4)
9'754'409	G	A	0/1	0/0	0/0	0/0	0/0	0/0	0/0	0/0	0/0	0/0	0/0	INTRON(MODIFIER|||NR3C2||rna18471|4)
9'754'443	T	C	0/1	0/0	0/0	0/0	0/0	0/0	0/0	0/0	0/0	0/0	0/0	INTRON(MODIFIER|||NR3C2||rna18471|4)
9'754'469	G	A	0/1	0/0	0/0	0/0	0/0	0/0	0/0	0/0	0/0	0/0	0/0	INTRON(MODIFIER|||NR3C2||rna18471|4)

9'754'554	C	T	0/1	0/0	0/0	0/0	0/0	0/0	0/0	0/0	0/0	0/0	0/0	INTRON(MODIFIER|||NR3C2||rna18471|4)
9'754'913	C	T	0/1	0/0	0/0	0/0	0/0	0/0	0/0	0/0	0/0	0/0	0/0	INTRON(MODIFIER|||NR3C2||rna18471|4)
9'754'979	A	C	0/1	0/0	0/0	0/0	0/0	0/0	0/0	0/0	0/0	0/0	0/0	INTRON(MODIFIER|||NR3C2||rna18471|4)
9'755'048	G	A	0/1	0/0	0/0	0/0	0/0	0/0	0/0	0/0	0/0	0/0	0/0	INTRON(MODIFIER|||NR3C2||rna18471|4)
9'755'103	A	G	0/1	0/0	0/0	0/0	0/0	0/0	0/0	0/0	0/0	0/0	0/0	INTRON(MODIFIER|||NR3C2||rna18471|4)
9'755'424	C	T	0/1	0/0	0/0	0/0	0/0	0/0	0/0	0/0	0/0	0/0	0/0	INTRON(MODIFIER|||NR3C2||rna18471|4)
9'755'448	C	T	0/1	0/0	0/0	0/0	0/0	0/0	0/0	0/0	0/0	0/0	0/0	INTRON(MODIFIER|||NR3C2||rna18471|4)
9'755'547	G	C	0/1	0/0	0/0	0/0	0/0	0/0	0/0	0/0	0/0	0/0	0/0	INTRON(MODIFIER|||NR3C2||rna18471|4)
9'755'612	T	C	0/1	0/0	0/0	0/0	0/0	0/0	0/0	0/0	0/0	0/0	0/0	INTRON(MODIFIER|||NR3C2||rna18471|4)
9'755'639	A	T	0/1	0/0	0/0	0/0	0/0	0/0	0/0	0/0	0/0	0/0	0/0	INTRON(MODIFIER|||NR3C2||rna18471|4)
9'756'091	G	T	0/1	0/0	0/0	0/0	0/0	0/0	0/0	0/0	0/0	0/0	0/0	INTRON(MODIFIER|||NR3C2||rna18471|4)
9'757'480	A	C	0/1	0/0	0/0	0/0	0/0	0/0	0/0	0/0	0/0	0/0	0/0	INTRON(MODIFIER|||NR3C2||rna18471|4)
9'759'018	G	A	0/1	0/0	0/0	0/0	0/0	0/0	0/0	0/0	0/0	0/0	0/0	INTRON(MODIFIER|||NR3C2||rna18471|4)
9'760'211	G	GA	0/1	0/0	0/0	0/0	0/0	0/0	0/0	0/0	0/0	0/0	0/0	INTRON(MODIFIER|||NR3C2||rna18471|4)
9'764'186	C	T	0/1	0/0	0/0	0/0	0/0	0/0	0/0	0/0	0/0	0/0	0/0	INTRON(MODIFIER|||NR3C2||rna18471|4)
9'764'600	A	G	0/1	0/0	0/0	0/0	0/0	0/0	0/0	0/0	0/0	0/0	0/0	INTRON(MODIFIER|||NR3C2||rna18471|4)
9'768'941	A	C	0/1	0/0	0/0	0/0	0/0	0/0	0/0	0/0	0/0	0/0	0/0	INTRON(MODIFIER|||NR3C2||rna18471|4)
9'771'183	G	C	0/1	0/0	0/0	0/0	0/0	0/0	0/0	0/0	0/0	0/0	0/0	INTRON(MODIFIER|||NR3C2||rna18471|5)
9'772'930	G	A	0/1	0/0	0/0	0/0	0/0	0/0	0/0	0/0	0/0	0/0	0/0	INTRON(MODIFIER|||NR3C2||rna18471|5)
9'776'610	C	T	0/1	0/0	0/0	0/0	0/0	0/0	0/0	0/0	0/0	0/0	0/0	INTRON(MODIFIER|||NR3C2||rna18471|6)
9'778'135	C	T	0/1	0/0	0/0	0/0	0/0	0/0	0/0	0/0	0/0	0/0	0/0	INTRON(MODIFIER|||NR3C2||rna18471|6)
9'782'344	G	A	0/1	0/0	0/0	0/0	0/0	0/0	0/0	0/0	0/0	0/0	0/0	INTRON(MODIFIER|||NR3C2||rna18471|6)
9'785'122	T	G	0/1	0/0	0/0	0/0	0/0	0/0	0/0	0/0	0/0	0/0	0/0	INTRON(MODIFIER|||NR3C2||rna18471|6)
9'788'324	A	G	0/1	0/0	0/0	0/0	0/0	0/0	0/0	0/0	0/0	0/0	0/0	INTRON(MODIFIER|||NR3C2||rna18471|6)
9'794'303	C	T	0/1	0/0	0/0	0/0	0/0	0/0	0/0	0/0	0/0	0/0	0/0	INTRON(MODIFIER|||NR3C2||rna18471|6)
9'796'984	G	T	0/1	0/0	0/0	0/0	0/0	0/0	0/0	0/0	0/0	0/0	0/0	INTRON(MODIFIER|||NR3C2||rna18471|6)
9'797'814	G	T	0/1	0/0	0/0	0/0	0/0	0/0	0/0	0/0	0/0	0/0	0/0	INTRON(MODIFIER|||NR3C2||rna18471|6)
9'800'421	AAGTTCAC	A	0/1	0/0	0/0	0/0	0/0	0/0	0/0	0/0	0/0	0/0	0/0	INTRON(MODIFIER|||NR3C2||rna18471|6)
9'802'060	T	C	0/1	0/0	0/0	0/0	0/0	0/0	0/0	0/0	0/0	0/0	0/0	INTRON(MODIFIER|||NR3C2||rna18471|6)
9'805'351	T	C	0/1	0/0	0/0	0/0	0/0	0/0	0/0	0/0	0/0	0/0	0/0	INTRON(MODIFIER|||NR3C2||rna18471|6)
9'809'489	G	A	0/1	0/0	0/0	0/0	0/0	0/0	0/0	0/0	0/0	0/0	0/0	INTRON(MODIFIER|||NR3C2||rna18471|6)
9'812'797	G	A	0/1	0/0	0/0	0/0	0/0	0/0	0/0	0/0	0/0	0/0	0/0	INTRON(MODIFIER|||NR3C2||rna18471|6)
9'817'632	C	T	0/1	0/0	0/0	0/0	0/0	0/0	0/0	0/0	0/0	0/0	0/0	INTRON(MODIFIER|||NR3C2||rna18471|7)
9'825'324	C	T	0/1	0/0	0/0	0/0	0/0	0/0	0/0	0/0	0/0	0/0	0/0	INTRON(MODIFIER|||NR3C2||rna18471|8)
9'825'677	T	A	0/1	0/0	0/0	0/0	0/0	0/0	0/0	0/0	0/0	0/0	0/0	INTRON(MODIFIER|||NR3C2||rna18471|8)
9'827'631	G	A	0/1	0/0	0/0	0/0	0/0	0/0	0/0	0/0	0/0	0/0	0/0	INTRON(MODIFIER|||NR3C2||rna18471|8)
9'841'424	T	C	0/1	0/0	0/0	0/0	0/0	0/0	0/0	0/0	0/0	0/0	0/0	INTRON(MODIFIER|||NR3C2||rna18471|8)
9'845'984	A	G	0/1	0/0	0/0	0/0	0/0	0/0	0/0	0/0	0/0	0/0	0/0	INTRON(MODIFIER|||NR3C2||rna18471|8)
9'847'423	T	G	0/1	0/0	0/0	0/0	0/0	0/0	0/0	0/0	0/0	0/0	0/0	INTRON(MODIFIER|||NR3C2||rna18471|8)
9'856'904	C	T	0/1	0/0	0/0	0/0	0/0	0/0	0/0	0/0	0/0	0/0	0/0	INTRON(MODIFIER|||NR3C2||rna18471|8)
9'857'807	C	T	0/1	0/0	0/0	0/0	0/0	0/0	0/0	0/0	0/0	0/0	0/0	INTRON(MODIFIER|||NR3C2||rna18471|8)
9'861'754	C	G	0/1	0/0	0/0	0/0	0/0	0/0	0/0	0/0	0/0	0/0	0/0	SYNONYMOUS_CODING(LOW|SILENT|acC/acG|T978|NR3C2||rna18471|9)
9'863'916	G	A	0/1	0/0	0/0	0/0	0/0	0/0	0/0	0/0	0/0	0/0	0/0	UTR_3_PRIME(MODIFIER|||NR3C2||rna18471|9)
9'871'414	G	C	0/1	0/0	0/0	0/0	0/0	0/0	0/0	0/0	0/0	0/0	0/0	UTR_3_PRIME(MODIFIER|||ARHGAP10||rna18472|18)
9'873'052	G	A	0/1	0/0	0/0	0/0	0/0	0/0	0/0	0/0	0/0	0/0	0/0	INTRON(MODIFIER|||ARHGAP10||rna18472|17)
9'873'085	A	G	0/1	0/0	0/0	0/0	0/0	0/0	0/0	0/0	0/0	0/0	0/0	INTRON(MODIFIER|||ARHGAP10||rna18472|17)
9'880'586	C	T	0/1	0/0	0/0	0/0	0/0	0/0	0/0	0/0	0/0	0/0	0/0	INTRON(MODIFIER|||ARHGAP10||rna18472|15),UPSTREAM(MODIFIER||955||TRNAC-ACA||rna18473|)
9'885'406	C	T	0/1	0/0	0/0	0/0	0/0	0/0	0/0	0/0	0/0	0/0	0/0	DOWNSTREAM(MODIFIER||3794||TRNAC-ACA||rna18473|),INTRON(MODIFIER|||ARHGAP10||rna18472|15)
9'886'667	C	CT	0/1	0/0	0/0	0/0	0/0	0/0	0/0	0/0	0/0	0/0	0/0	INTRON(MODIFIER|||ARHGAP10||rna18472|15)
9'888'906	G	A	0/1	0/0	0/0	0/0	0/0	0/0	0/0	0/0	0/0	0/0	0/0	INTRON(MODIFIER|||ARHGAP10||rna18472|15)
9'890'567	G	A	0/1	0/0	0/0	0/0	0/0	0/0	0/0	0/0	0/0	0/0	0/0	INTRON(MODIFIER|||ARHGAP10||rna18472|15)
9'891'905	T	C	0/1	0/0	0/0	0/0	0/0	0/0	0/0	0/0	0/0	0/0	0/0	INTRON(MODIFIER|||ARHGAP10||rna18472|15)
9'900'514	C	T	0/1	0/0	0/0	0/0	0/0	0/0	0/0	0/0	0/0	0/0	0/0	INTRON(MODIFIER|||ARHGAP10||rna18472|14)
9'901'296	A	G	0/1	0/0	0/0	0/0	0/0	0/0	0/0	0/0	0/0	0/0	0/0	INTRON(MODIFIER|||ARHGAP10||rna18472|14)
9'901'367	G	A	0/1	0/0	0/0	0/0	0/0	0/0	0/0	0/0	0/0	0/0	0/0	INTRON(MODIFIER|||ARHGAP10||rna18472|14)
9'901'587	C	T	0/1	0/0	0/0	0/0	0/0	0/0	0/0	0/0	0/0	0/0	0/0	INTRON(MODIFIER|||ARHGAP10||rna18472|14)
9'902'726	T	C	0/1	0/0	0/0	0/0	0/0	0/0	0/0	0/0	0/0	0/0	0/0	INTRON(MODIFIER|||ARHGAP10||rna18472|14)
9'903'277	C	T	0/1	0/0	0/0	0/0	0/0	0/0	0/0	0/0	0/0	0/0	0/0	INTRON(MODIFIER|||ARHGAP10||rna18472|14)
9'904'171	T	C	0/1	0/0	0/0	0/0	0/0	0/0	0/0	0/0	0/0	0/0	0/0	INTRON(MODIFIER|||ARHGAP10||rna18472|14)
9'905'711	A	G	0/1	0/0	0/0	0/0	0/0	0/0	0/0	0/0	0/0	0/0	0/0	INTRON(MODIFIER|||ARHGAP10||rna18472|14)
9'908'081	C	A	0/1	0/0	0/0	0/0	0/0	0/0	0/0	0/0	0/0	0/0	0/0	INTRON(MODIFIER|||ARHGAP10||rna18472|14)
9'910'110	G	T	0/1	0/0	0/0	0/0	0/0	0/0	0/0	0/0	0/0	0/0	0/0	INTRON(MODIFIER|||ARHGAP10||rna18472|14)
9'911'583	A	T	0/1	0/0	0/0	0/0	0/0	0/0	0/0	0/0	0/0	0/0	0/0	INTRON(MODIFIER|||ARHGAP10||rna18472|14)
9'912'729	A	G	0/1	0/0	0/0	0/0	0/0	0/0	0/0	0/0	0/0	0/0	0/0	INTRON(MODIFIER|||ARHGAP10||rna18472|14)
9'913'137	C	T	0/1	0/0	0/0	0/0	0/0	0/0	0/0	0/0	0/0	0/0	0/0	INTRON(MODIFIER|||ARHGAP10||rna18472|14)
9'915'681	A	G	0/1	0/0	0/0	0/0	0/0	0/0	0/0	0/0	0/0	0/0	0/0	INTRON(MODIFIER|||ARHGAP10||rna18472|14)
9'917'417	T	C	0/1	0/0	0/0	0/0	0/0	0/0	0/0	0/0	0/0	0/0	0/0	INTRON(MODIFIER|||ARHGAP10||rna18472|14)
9'919'052	T	A	0/1	0/0	0/0	0/0	0/0	0/0	0/0	0/0	0/0	0/0	0/0	INTRON(MODIFIER|||ARHGAP10||rna18472|14)
9'920'137	C	T	0/1	0/0	0/0	0/0	0/0	0/0	0/0	0/0	0/0	0/0	0/0	INTRON(MODIFIER|||ARHGAP10||rna18472|14)
9'920'527	A	G	0/1	0/0	0/0	0/0	0/0	0/0	0/0	0/0	0/0	0/0	0/0	INTRON(MODIFIER|||ARHGAP10||rna18472|14)
9'920'733	A	T	0/1	0/0	0/0	0/0	0/0	0/0	0/0	0/0	0/0	0/0	0/0	INTRON(MODIFIER|||ARHGAP10||rna18472|14)
9'922'055	CA	C	0/1	0/0	0/0	0/0	0/0	0/0	0/0	0/0	0/0	0/0	0/0	INTRON(MODIFIER|||ARHGAP10||rna18472|14)
9'924'363	C	T	0/1	0/0	0/0	0/0	0/0	0/0	0/0	0/0	0/0	0/0	0/0	INTRON(MODIFIER|||ARHGAP10||rna18472|14)
9'924'734	C	CAAAAT	0/1	0/0	0/0	0/0	0/0	0/0	0/0	0/0	0/0	0/0	0/0	INTRON(MODIFIER|||ARHGAP10||rna18472|14)
9'924'762	G	A	0/1	0/0	0/0	0/0	0/0	0/0	0/0	0/0	0/0	0/0	0/0	INTRON(MODIFIER|||ARHGAP10||rna18472|14)
9'925'157	A	C	0/1	0/0	0/0	0/0	0/0	0/0	0/0	0/0	0/0	0/0	0/0	INTRON(MODIFIER|||ARHGAP10||rna18472|14)
9'927'583	C	T	0/1	0/0	0/0	0/0	0/0	0/0	0/0	0/0	0/0	0/0	0/0	INTRON(MODIFIER|||ARHGAP10||rna18472|14)
9'928'036	T	C	0/1	0/0	0/0	0/0	0/0	0/0	0/0	0/0	0/0	0/0	0/0	INTRON(MODIFIER|||ARHGAP10||rna18472|14)
9'928'771	T	C	0/1	0/0	0/0	0/0	0/0	0/0	0/0	0/0	0/0	0/0	0/0	INTRON(MODIFIER|||ARHGAP10||rna18472|13)
9'928'814	T	C	0/1	0/0	0/0	0/0	0/0	0/0	0/0	0/0	0/0	0/0	0/0	INTRON(MODIFIER|||ARHGAP10||rna18472|13)
9'929'140	A	AAAAT	0/1	0/0	0/0	0/0	0/0	0/0	0/0	0/0	0/0	0/0	0/0	INTRON(MODIFIER|||ARHGAP10||rna18472|13)
9'933'872	A	T	0/1	0/0	0/0	0/0	0/0	0/0	0/0	0/0	0/0	0/0	0/0	INTRON(MODIFIER|||ARHGAP10||rna18472|13)
9'933'922	A	T	0/1	0/0	0/0	0/0	0/0	0/0	0/0	0/0	0/0	0/0	0/0	INTRON(MODIFIER|||ARHGAP10||rna18472|13)
9'935'553	C	T	0/1	0/0	0/0	0/0	0/0	0/0	0/0	0/0	0/0	0/0	0/0	INTRON(MODIFIER|||ARHGAP10||rna18472|13)
9'935'870	A	G	0/1	0/0	0/0	0/0	0/0	0/0	0/0	0/0	0/0	0/0	0/0	INTRON(MODIFIER|||ARHGAP10||rna18472|13)
9'938'224	AT	A	0/1	0/0	0/0	0/0	0/0	0/0	0/0	./.	0/0	0/0	0/0	INTRON(MODIFIER|||ARHGAP10||rna18472|13)
9'941'139	C	T	0/1	0/0	0/0	0/0	0/0	0/0	0/0	0/0	0/0	0/0	0/0	INTRON(MODIFIER|||ARHGAP10||rna18472|13)
9'941'186	A	G	0/1	0/0	0/0	0/0	0/0	0/0	0/0	0/0	0/0	0/0	0/0	INTRON(MODIFIER|||ARHGAP10||rna18472|13)
9'941'213	A	C	0/1	0/0	0/0	0/0	0/0	0/0	0/0	0/0	0/0	0/0	0/0	INTRON(MODIFIER|||ARHGAP10||rna18472|13)
9'941'219	G	T	0/1	0/0	0/0	0/0	0/0	0/0	0/0	0/0	0/0	0/0	0/0	INTRON(MODIFIER|||ARHGAP10||rna18472|13)
9'943'599	C	T	0/1	0/0	0/0	0/0	0/0	0/0	0/0	0/0	0/0	0/0	0/0	INTRON(MODIFIER|||ARHGAP10||rna18472|13)
9'946'953	G	A	0/1	0/0	0/0	0/0	0/0	0/0	0/0	0/0	0/0	0/0	0/0	INTRON(MODIFIER|||ARHGAP10||rna18472|13)
9'951'826	C	T	0/1	0/0	0/0	0/0	0/0	0/0	0/0	0/0	0/0	0/0	0/0	INTRON(MODIFIER|||ARHGAP10||rna18472|13)
9'974'287	G	C	0/1	0/0	0/0	0/0	0/0	0/0	0/0	0/0	0/0	0/0	0/0	INTRON(MODIFIER|||ARHGAP10||rna18472|13)
9'990'908	G	A	0/1	0/0	0/0	0/0	0/0	0/0	0/0	0/0	0/0	0/0	0/0	INTRON(MODIFIER|||ARHGAP10||rna18472|11)
9'991'862	C	T	0/1	0/0	0/0	0/0	0/0	0/0	0/0	0/0	0/0	0/0	0/0	INTRON(MODIFIER|||ARHGAP10||rna18472|11)
9'991'948	A	C	0/1	0/0	0/0	0/0	0/0	0/0	0/0	0/0	0/0	0/0	0/0	INTRON(MODIFIER|||ARHGAP10||rna18472|11)
9'993'124	G	A	0/1	0/0	0/0	0/0	0/0	0/0	0/0	0/0	0/0	0/0	0/0	INTRON(MODIFIER|||ARHGAP10||rna18472|11)
9'994'066	A	G	0/1	0/0	0/0	0/0	0/0	0/0	0/0	0/0	0/0	0/0	0/0	INTRON(MODIFIER|||ARHGAP10||rna18472|11)
9'994'320	T	TA	0/1	0/0	0/0	0/0	0/0	0/0	0/0	0/0	0/0	0/0	0/0	INTRON(MODIFIER|||ARHGAP10||rna18472|11)
9'994'558	T	C	0/1	0/0	0/0	0/0	0/0	0/0	0/0	0/0	0/0	0/0	0/0	INTRON(MODIFIER|||ARHGAP10||rna18472|11)
9'994'734	C	A	0/1	0/0	0/0	0/0	0/0	0/0	0/0	0/0	0/0	0/0	0/0	INTRON(MODIFIER|||ARHGAP10||rna18472|11)
9'995'031	C	T	0/1	0/0	0/0	0/0	0/0	0/0	0/0	0/0	0/0	0/0	0/0	INTRON(MODIFIER|||ARHGAP10||rna18472|11)
9'995'947	T	C	0/1	0/0	0/0	0/0	0/0	0/0	0/0	0/0	0/0	0/0	0/0	INTRON(MODIFIER|||ARHGAP10||rna18472|11)
9'996'075	A	C	0/1	0/0	0/0	0/0	0/0	0/0	0/0	0/0	0/0	0/0	0/0	INTRON(MODIFIER|||ARHGAP10||rna18472|11)
9'999'480	T	A	0/1	0/0	0/0	0/0	0/0	0/0	0/0	0/0	0/0	0/0	0/0	INTRON(MODIFIER|||ARHGAP10||rna18472|11)
10'001'821	T	C	0/1	0/0	0/0	0/0	0/0	0/0	0/0	0/0	0/0	0/0	0/0	INTRON(MODIFIER|||ARHGAP10||rna18472|10)
10'002'248	G	A	0/1	0/0	0/0	0/0	0/0	0/0	0/0	0/0	0/0	0/0	0/0	INTRON(MODIFIER|||ARHGAP10||rna18472|10)
10'006'772	A	C	0/1	0/0	0/0	0/0	0/0	0/0	0/0	0/0	0/0	0/0	0/0	INTRON(MODIFIER|||ARHGAP10||rna18472|10)
10'006'895	C	T	0/1	0/0	0/0	0/0	0/0	0/0	0/0	0/0	0/0	0/0	0/0	INTRON(MODIFIER|||ARHGAP10||rna18472|10)
10'006'903	T	C	0/1	0/0	0/0	0/0	0/0	0/0	0/0	0/0	0/0	0/0	0/0	INTRON(MODIFIER|||ARHGAP10||rna18472|10)
10'007'043	A	T	0/1	0/0	0/0	0/0	0/0	0/0	0/0	./.	0/0	0/0	0/0	INTRON(MODIFIER|||ARHGAP10||rna18472|10)
10'012'310	T	C	0/1	0/0	0/0	0/0	0/0	0/0	0/0	0/0	0/0	0/0	0/0	INTRON(MODIFIER|||ARHGAP10||rna18472|9)
10'015'774	C	T	0/1	0/0	0/0	0/0	0/0	0/0	0/0	0/0	0/0	0/0	0/0	INTRON(MODIFIER|||ARHGAP10||rna18472|8)
10'015'803	G	A	0/1	0/0	0/0	0/0	0/0	0/0	0/0	0/0	0/0	0/0	0/0	INTRON(MODIFIER|||ARHGAP10||rna18472|8)
10'016'357	C	G	0/1	0/0	0/0	0/0	0/0	0/0	0/0	0/0	0/0	0/0	0/0	INTRON(MODIFIER|||ARHGAP10||rna18472|8)
10'021'533	T	G	0/1	0/0	0/0	0/0	0/0	0/0	0/0	0/0	0/0	0/0	0/0	INTRON(MODIFIER|||ARHGAP10||rna18472|8)
10'023'449	TTGA	T	0/1	0/0	0/0	0/0	0/0	0/0	0/0	0/0	0/0	0/0	0/0	INTRON(MODIFIER|||ARHGAP10||rna18472|8)
10'023'454	G	T	0/1	0/0	0/0	0/0	0/0	0/0	0/0	0/0	0/0	0/0	0/0	INTRON(MODIFIER|||ARHGAP10||rna18472|8)
10'023'914	C	T	0/1	0/0	0/0	0/0	0/0	0/0	0/0	0/0	0/0	0/0	0/0	INTRON(MODIFIER|||ARHGAP10||rna18472|8)
10'024'091	G	A	0/1	0/0	0/0	0/0	0/0	0/0	0/0	0/0	0/0	0/0	0/0	INTRON(MODIFIER|||ARHGAP10||rna18472|8)
10'024'941	G	GTA	0/1	0/0	0/0	0/0	0/0	0/0	0/0	0/0	0/0	0/0	0/0	INTRON(MODIFIER|||ARHGAP10||rna18472|8)
10'025'203	A	T	0/1	0/0	0/0	0/0	0/0	0/0	0/0	0/0	0/0	0/0	0/0	INTRON(MODIFIER|||ARHGAP10||rna18472|8)
10'025'216	G	A	0/1	0/0	0/0	0/0	0/0	0/0	0/0	0/0	0/0	0/0	0/0	INTRON(MODIFIER|||ARHGAP10||rna18472|8)
10'026'959	A	AGG	0/1	0/0	0/0	0/0	0/0	0/0	0/0	0/0	0/0	0/0	0/0	INTRON(MODIFIER|||ARHGAP10||rna18472|8)
10'028'869	G	A	0/1	0/0	0/0	0/0	0/0	0/0	0/0	0/0	0/0	0/0	0/0	INTRON(MODIFIER|||ARHGAP10||rna18472|8)
10'029'835	G	A	0/1	0/0	0/0	0/0	0/0	0/0	0/0	0/0	0/0	0/0	0/0	INTRON(MODIFIER|||ARHGAP10||rna18472|8)
10'030'655	C	T	0/1	0/0	0/0	0/0	0/0	0/0	0/0	0/0	0/0	0/0	0/0	INTRON(MODIFIER|||ARHGAP10||rna18472|8)
10'036'398	A	C	0/1	0/0	0/0	0/0	0/0	0/0	0/0	0/0	0/0	0/0	0/0	INTRON(MODIFIER|||ARHGAP10||rna18472|8)
10'041'529	C	G	0/1	0/0	0/0	0/0	0/0	0/0	0/0	0/0	0/0	0/0	0/0	INTRON(MODIFIER|||ARHGAP10||rna18472|7)
10'043'159	T	C	0/1	0/0	0/0	0/0	0/0	0/0	0/0	0/0	0/0	0/0	0/0	INTRON(MODIFIER|||ARHGAP10||rna18472|6)
10'043'972	A	G	0/1	0/0	0/0	0/0	0/0	0/0	0/0	0/0	0/0	0/0	0/0	INTRON(MODIFIER|||ARHGAP10||rna18472|6)
10'044'904	A	T	0/1	0/0	0/0	0/0	0/0	0/0	0/0	0/0	0/0	0/0	0/0	INTRON(MODIFIER|||ARHGAP10||rna18472|5)
10'045'371	C	T	0/1	0/0	0/0	0/0	0/0	0/0	0/0	0/0	0/0	0/0	0/0	INTRON(MODIFIER|||ARHGAP10||rna18472|5)
10'045'523	T	C	0/1	0/0	0/0	0/0	0/0	0/0	0/0	0/0	0/0	0/0	0/0	INTRON(MODIFIER|||ARHGAP10||rna18472|5)
10'045'565	G	A	0/1	0/0	0/0	0/0	0/0	0/0	0/0	0/0	0/0	0/0	0/0	INTRON(MODIFIER|||ARHGAP10||rna18472|5)
10'046'143	G	A	0/1	0/0	0/0	0/0	0/0	0/0	0/0	0/0	0/0	0/0	0/0	INTRON(MODIFIER|||ARHGAP10||rna18472|5)
10'046'535	T	C	0/1	0/0	0/0	./.	./.	0/0	./.	0/0	./.	0/0	0/0	INTRON(MODIFIER|||ARHGAP10||rna18472|5)
10'047'524	G	A	0/1	0/0	0/0	0/0	0/0	0/0	0/0	0/0	0/0	0/0	0/0	INTRON(MODIFIER|||ARHGAP10||rna18472|5)
10'049'376	C	A	0/1	0/0	0/0	0/0	0/0	0/0	0/0	0/0	0/0	0/0	0/0	INTRON(MODIFIER|||ARHGAP10||rna18472|5)
10'049'670	A	C	0/1	0/0	0/0	0/0	0/0	0/0	0/0	0/0	0/0	0/0	0/0	INTRON(MODIFIER|||ARHGAP10||rna18472|5)
10'049'969	G	T	0/1	0/0	0/0	0/0	0/0	0/0	0/0	0/0	0/0	0/0	0/0	INTRON(MODIFIER|||ARHGAP10||rna18472|5)
10'050'449	T	G	0/1	0/0	./.	0/0	0/0	0/0	0/0	0/0	0/0	0/0	0/0	INTRON(MODIFIER|||ARHGAP10||rna18472|5)

10'050'683	A	G	0/1	0/0	0/0	0/0	0/0	0/0	0/0	0/0	0/0	0/0	0/0	INTRON(MODIFIER|||ARHGAP10||rna18472|5)
10'051'596	AGAGACACGG	A	0/1	0/0	0/0	0/0	0/0	0/0	0/0	0/0	0/0	0/0	0/0	INTRON(MODIFIER|||ARHGAP10||rna18472|5)
10'052'425	T	C	0/1	0/0	0/0	0/0	./.	0/0	0/0	0/0	0/0	0/0	0/0	INTRON(MODIFIER|||ARHGAP10||rna18472|5)
10'053'289	C	T	0/1	0/0	0/0	0/0	0/0	0/0	0/0	0/0	0/0	0/0	0/0	INTRON(MODIFIER|||ARHGAP10||rna18472|5)
10'053'522	G	A	0/1	0/0	0/0	0/0	0/0	0/0	0/0	0/0	0/0	0/0	0/0	INTRON(MODIFIER|||ARHGAP10||rna18472|5)
10'054'859	T	C	0/1	0/0	0/0	0/0	0/0	0/0	0/0	0/0	0/0	0/0	0/0	INTRON(MODIFIER|||ARHGAP10||rna18472|5)
10'056'635	G	A	0/1	0/0	0/0	0/0	0/0	0/0	0/0	0/0	0/0	0/0	0/0	INTRON(MODIFIER|||ARHGAP10||rna18472|5)
10'058'844	G	A	0/1	0/0	0/0	0/0	0/0	0/0	0/0	0/0	0/0	0/0	0/0	INTRON(MODIFIER|||ARHGAP10||rna18472|5)
10'058'937	A	G	0/1	0/0	0/0	0/0	0/0	0/0	0/0	0/0	0/0	0/0	0/0	INTRON(MODIFIER|||ARHGAP10||rna18472|5)
10'060'028	T	C	0/1	0/0	0/0	0/0	0/0	0/0	0/0	0/0	0/0	0/0	0/0	INTRON(MODIFIER|||ARHGAP10||rna18472|5)
10'062'561	T	G	0/1	0/0	0/0	0/0	0/0	0/0	0/0	0/0	0/0	0/0	0/0	INTRON(MODIFIER|||ARHGAP10||rna18472|5)
10'062'648	A	G	0/1	0/0	0/0	0/0	0/0	0/0	0/0	0/0	0/0	0/0	0/0	INTRON(MODIFIER|||ARHGAP10||rna18472|5)
10'063'578	T	C	0/1	0/0	0/0	0/0	0/0	0/0	0/0	0/0	0/0	0/0	0/0	INTRON(MODIFIER|||ARHGAP10||rna18472|5)
10'065'954	T	C	0/1	0/0	0/0	0/0	0/0	0/0	0/0	0/0	0/0	0/0	0/0	INTRON(MODIFIER|||ARHGAP10||rna18472|5)
10'068'651	T	G	0/1	0/0	0/0	0/0	0/0	0/0	0/0	0/0	0/0	0/0	0/0	INTRON(MODIFIER|||ARHGAP10||rna18472|5)
10'092'000	G	C	0/1	0/0	0/0	0/0	0/0	0/0	0/0	0/0	0/0	0/0	0/0	INTRON(MODIFIER|||ARHGAP10||rna18472|2)
10'092'922	G	C	0/1	0/0	0/0	0/0	0/0	0/0	0/0	0/0	0/0	0/0	0/0	INTRON(MODIFIER|||ARHGAP10||rna18472|2)
10'093'277	C	G	0/1	0/0	0/0	0/0	0/0	0/0	0/0	0/0	0/0	0/0	0/0	INTRON(MODIFIER|||ARHGAP10||rna18472|2)
10'093'692	A	G	0/1	0/0	0/0	0/0	0/0	0/0	0/0	0/0	0/0	0/0	0/0	INTRON(MODIFIER|||ARHGAP10||rna18472|2)
10'093'894	C	CA	0/1	0/0	0/0	0/0	0/0	0/0	0/0	0/0	0/0	0/0	0/0	INTRON(MODIFIER|||ARHGAP10||rna18472|2)
10'093'936	G	A	0/1	0/0	0/0	0/0	0/0	0/0	0/0	0/0	0/0	0/0	0/0	INTRON(MODIFIER|||ARHGAP10||rna18472|2)
10'094'325	C	A	0/1	0/0	0/0	0/0	0/0	0/0	0/0	0/0	0/0	0/0	0/0	INTRON(MODIFIER|||ARHGAP10||rna18472|2)
10'094'357	T	C	0/1	0/0	0/0	0/0	0/0	0/0	0/0	0/0	0/0	0/0	0/0	INTRON(MODIFIER|||ARHGAP10||rna18472|2)
10'094'414	C	T	0/1	0/0	0/0	0/0	0/0	0/0	0/0	0/0	0/0	0/0	0/0	INTRON(MODIFIER|||ARHGAP10||rna18472|2)
10'094'676	T	A	0/1	0/0	0/0	0/0	0/0	0/0	0/0	0/0	0/0	0/0	0/0	INTRON(MODIFIER|||ARHGAP10||rna18472|2)
10'094'718	C	T	0/1	0/0	0/0	0/0	0/0	0/0	0/0	0/0	0/0	0/0	0/0	INTRON(MODIFIER|||ARHGAP10||rna18472|2)
10'094'798	C	A	0/1	0/0	0/0	0/0	0/0	0/0	0/0	0/0	0/0	0/0	0/0	INTRON(MODIFIER|||ARHGAP10||rna18472|2)
10'094'994	T	C	0/1	0/0	0/0	0/0	0/0	0/0	0/0	0/0	0/0	0/0	0/0	INTRON(MODIFIER|||ARHGAP10||rna18472|2)
10'095'300	TG	T	0/1	0/0	0/0	0/0	0/0	0/0	0/0	0/0	0/0	0/0	0/0	INTRON(MODIFIER|||ARHGAP10||rna18472|2)
10'095'483	T	G	0/1	0/0	0/0	0/0	0/0	0/0	0/0	0/0	0/0	0/0	0/0	INTRON(MODIFIER|||ARHGAP10||rna18472|2)
10'095'568	GTCTTTT	G	0/1	0/0	0/0	0/0	0/0	0/0	0/0	0/0	0/0	0/0	0/0	INTRON(MODIFIER|||ARHGAP10||rna18472|2)
10'096'349	A	G	0/1	0/0	0/0	0/0	0/0	0/0	0/0	0/0	0/0	0/0	0/0	INTRON(MODIFIER|||ARHGAP10||rna18472|1)
10'096'548	A	T	0/1	0/0	0/0	0/0	0/0	0/0	0/0	0/0	0/0	0/0	0/0	INTRON(MODIFIER|||ARHGAP10||rna18472|1)
10'096'590	A	G	0/1	0/0	0/0	0/0	0/0	0/0	0/0	0/0	0/0	0/0	0/0	INTRON(MODIFIER|||ARHGAP10||rna18472|1)
10'101'524	A	G	0/1	0/0	0/0	0/0	0/0	0/0	0/0	0/0	0/0	0/0	0/0	INTERGENIC(MODIFIER),UPSTREAM(MODIFIER||3240||ARHGAP10||rna18472|)
10'101'710	A	G	0/1	0/0	0/0	0/0	0/0	0/0	0/0	0/0	0/0	0/0	0/0	INTERGENIC(MODIFIER),UPSTREAM(MODIFIER||3426||ARHGAP10||rna18472|)
10'102'068	C	G	0/1	0/0	0/0	0/0	0/0	0/0	0/0	0/0	0/0	0/0	0/0	INTERGENIC(MODIFIER),UPSTREAM(MODIFIER||3784||ARHGAP10||rna18472|)
10'102'157	AAGG	A	0/1	0/0	0/0	0/0	0/0	0/0	0/0	0/0	0/0	0/0	0/0	INTERGENIC(MODIFIER),UPSTREAM(MODIFIER||3874||ARHGAP10||rna18472|)
10'102'359	G	A	0/1	0/0	0/0	0/0	0/0	0/0	0/0	0/0	0/0	0/0	0/0	INTERGENIC(MODIFIER),UPSTREAM(MODIFIER||4075||ARHGAP10||rna18472|)
10'102'371	T	A	0/1	0/0	0/0	0/0	0/0	0/0	0/0	0/0	0/0	0/0	0/0	INTERGENIC(MODIFIER),UPSTREAM(MODIFIER||4087||ARHGAP10||rna18472|)
10'102'389	G	A	0/1	0/0	0/0	0/0	0/0	0/0	0/0	0/0	0/0	0/0	0/0	INTERGENIC(MODIFIER),UPSTREAM(MODIFIER||4105||ARHGAP10||rna18472|)
10'102'462	G	A	0/1	0/0	0/0	0/0	0/0	0/0	0/0	0/0	0/0	0/0	0/0	INTERGENIC(MODIFIER),UPSTREAM(MODIFIER||4178||ARHGAP10||rna18472|)
10'102'671	A	G	0/1	0/0	0/0	0/0	0/0	0/0	0/0	0/0	0/0	0/0	0/0	INTERGENIC(MODIFIER),UPSTREAM(MODIFIER||4387||ARHGAP10||rna18472|)
10'102'976	C	CT	0/1	0/0	0/0	0/0	0/0	0/0	0/0	0/0	0/0	0/0	0/0	INTERGENIC(MODIFIER),UPSTREAM(MODIFIER||4693||ARHGAP10||rna18472|)
10'103'015	G	A	0/1	0/0	0/0	0/0	0/0	0/0	0/0	0/0	0/0	0/0	0/0	INTERGENIC(MODIFIER),UPSTREAM(MODIFIER||4731||ARHGAP10||rna18472|)
10'103'193	G	A	1/1	0/0	0/0	0/0	0/0	0/0	0/0	0/0	0/0	0/0	0/0	INTERGENIC(MODIFIER),UPSTREAM(MODIFIER||4909||ARHGAP10||rna18472|)
10'103'639	A	G	0/1	0/0	0/0	0/0	0/0	0/0	0/0	0/0	0/0	0/0	0/0	INTERGENIC(MODIFIER)
10'104'889	A	C	0/1	0/0	0/0	0/0	0/0	0/0	0/0	0/0	0/0	0/0	0/0	INTERGENIC(MODIFIER)
10'105'480	A	G	0/1	0/0	0/0	0/0	0/0	0/0	0/0	0/0	0/0	0/0	0/0	INTERGENIC(MODIFIER)
10'105'741	G	A	0/1	0/0	0/0	0/0	0/0	0/0	0/0	0/0	0/0	0/0	0/0	INTERGENIC(MODIFIER)
10'105'910	AAAAACAACAAC	A	1/1	0/0	0/0	0/0	0/0	0/0	0/0	0/0	0/0	0/0	0/0	INTERGENIC(MODIFIER)
10'105'979	A	G	1/1	0/0	0/0	0/0	0/0	0/0	0/0	0/0	0/0	0/0	0/0	INTERGENIC(MODIFIER)
10'106'186	C	T	0/1	0/0	0/0	0/0	0/0	0/0	0/0	0/0	0/0	0/0	0/0	INTERGENIC(MODIFIER)
10'106'379	C	T	0/1	0/0	0/0	0/0	0/0	0/0	0/0	0/0	0/0	0/0	./.	INTERGENIC(MODIFIER)
10'107'262	T	C	0/1	0/0	0/0	0/0	0/0	0/0	0/0	0/0	0/0	0/0	0/0	INTERGENIC(MODIFIER)
10'107'270	T	C	0/1	0/0	0/0	0/0	0/0	0/0	0/0	0/0	0/0	0/0	0/0	INTERGENIC(MODIFIER)
10'107'295	CT	C	0/1	0/0	0/0	0/0	0/0	0/0	0/0	0/0	0/0	0/0	0/0	INTERGENIC(MODIFIER)
10'107'299	T	C	0/1	0/0	0/0	0/0	0/0	0/0	0/0	0/0	0/0	0/0	0/0	INTERGENIC(MODIFIER)
10'107'827	A	G	0/1	0/0	0/0	0/0	0/0	0/0	0/0	0/0	0/0	0/0	0/0	INTERGENIC(MODIFIER)
10'109'464	G	A	0/1	0/0	0/0	0/0	0/0	0/0	0/0	0/0	0/0	0/0	0/0	INTERGENIC(MODIFIER)
10'114'151	T	C	0/1	0/0	0/0	0/0	0/0	0/0	0/0	0/0	0/0	0/0	0/0	INTERGENIC(MODIFIER)
10'117'936	T	TAGTAGC	0/1	0/0	0/0	0/0	0/0	0/0	0/0	0/0	0/0	0/0	0/0	INTERGENIC(MODIFIER)
10'118'267	A	G	0/1	0/0	0/0	0/0	0/0	0/0	0/0	0/0	0/0	0/0	0/0	INTERGENIC(MODIFIER)
10'119'056	T	C	0/1	0/0	0/0	0/0	0/0	0/0	0/0	0/0	0/0	0/0	0/0	INTERGENIC(MODIFIER)
10'119'150	G	A	0/1	0/0	0/0	0/0	0/0	0/0	0/0	0/0	0/0	0/0	0/0	INTERGENIC(MODIFIER)
10'119'165	A	G	0/1	0/0	0/0	0/0	0/0	0/0	0/0	0/0	0/0	0/0	0/0	INTERGENIC(MODIFIER)
10'119'786	AT	A	0/1	0/0	0/0	0/0	0/0	0/0	0/0	0/0	0/0	0/0	0/0	INTERGENIC(MODIFIER)
10'119'834	T	C	0/1	0/0	0/0	0/0	0/0	0/0	0/0	0/0	0/0	0/0	0/0	INTERGENIC(MODIFIER)
10'119'997	T	C	0/1	0/0	0/0	0/0	0/0	0/0	0/0	0/0	0/0	0/0	0/0	INTERGENIC(MODIFIER)
10'120'182	A	G	0/1	0/0	0/0	0/0	0/0	0/0	0/0	0/0	0/0	0/0	0/0	INTERGENIC(MODIFIER)
10'121'010	A	C	0/1	0/0	0/0	0/0	0/0	0/0	0/0	0/0	0/0	0/0	0/0	INTERGENIC(MODIFIER)
10'121'033	C	G	0/1	0/0	0/0	0/0	0/0	0/0	0/0	0/0	0/0	0/0	0/0	INTERGENIC(MODIFIER)
10'121'137	T	TGG	1/1	0/0	0/0	0/0	0/0	0/0	0/0	0/0	0/0	0/0	0/0	INTERGENIC(MODIFIER)
10'123'432	A	G	0/1	0/0	0/0	0/0	0/0	0/0	0/0	0/0	0/0	0/0	0/0	INTERGENIC(MODIFIER)
10'124'403	T	G	0/1	0/0	0/0	0/0	0/0	0/0	0/0	0/0	0/0	0/0	0/0	INTERGENIC(MODIFIER)
10'124'528	C	T	0/1	0/0	0/0	0/0	0/0	0/0	0/0	0/0	0/0	0/0	0/0	INTERGENIC(MODIFIER)
10'126'791	A	G	0/1	0/0	0/0	0/0	0/0	0/0	0/0	0/0	0/0	0/0	0/0	INTERGENIC(MODIFIER)
10'126'967	TG	T	0/1	0/0	0/0	0/0	0/0	0/0	0/0	0/0	0/0	0/0	0/0	INTERGENIC(MODIFIER)
10'127'338	G	A	0/1	0/0	0/0	0/0	0/0	0/0	0/0	0/0	0/0	0/0	0/0	INTERGENIC(MODIFIER)
10'127'639	G	A	0/1	0/0	0/0	0/0	0/0	0/0	0/0	0/0	0/0	0/0	0/0	INTERGENIC(MODIFIER)
10'128'136	G	A	0/1	0/0	0/0	0/0	0/0	0/0	0/0	0/0	0/0	0/0	0/0	INTERGENIC(MODIFIER)
10'128'232	T	C	0/1	0/0	0/0	0/0	0/0	0/0	0/0	0/0	0/0	0/0	0/0	INTERGENIC(MODIFIER)
10'130'240	C	T	0/1	0/0	0/0	0/0	0/0	0/0	0/0	0/0	0/0	0/0	0/0	INTERGENIC(MODIFIER)
10'130'305	C	G	0/1	0/0	0/0	0/0	0/0	0/0	0/0	0/0	0/0	0/0	0/0	INTERGENIC(MODIFIER)
10'130'336	G	A	0/1	0/0	0/0	0/0	0/0	0/0	0/0	0/0	0/0	0/0	0/0	INTERGENIC(MODIFIER)
10'131'691	C	T	0/1	0/0	0/0	0/0	0/0	0/0	0/0	0/0	0/0	0/0	0/0	INTERGENIC(MODIFIER)
10'132'792	T	G	0/1	0/0	0/0	0/0	0/0	0/0	0/0	0/0	0/0	0/0	0/0	INTERGENIC(MODIFIER)
10'142'185	A	C	0/1	0/0	0/0	0/0	0/0	0/0	0/0	0/0	0/0	0/0	0/0	INTERGENIC(MODIFIER)
10'142'613	G	A	0/1	0/0	0/0	0/0	0/0	0/0	0/0	0/0	0/0	0/0	0/0	INTERGENIC(MODIFIER)
10'145'493	T	G	0/1	0/0	0/0	0/0	0/0	0/0	0/0	0/0	0/0	0/0	0/0	INTERGENIC(MODIFIER)
10'148'280	C	T	0/1	0/0	0/0	0/0	0/0	0/0	0/0	0/0	0/0	0/0	0/0	DOWNSTREAM(MODIFIER||3442||id200169||rna18474|),INTERGENIC(MODIFIER)
10'152'039	C	T	0/1	0/0	0/0	0/0	0/0	0/0	0/0	0/0	0/0	0/0	0/0	INTERGENIC(MODIFIER),UPSTREAM(MODIFIER||95||id200169||rna18474|)
10'160'090	G	A	0/1	0/0	0/0	0/0	0/0	0/0	0/0	0/0	0/0	0/0	0/0	INTERGENIC(MODIFIER)
10'162'091	G	A	0/1	0/0	0/0	0/0	0/0	0/0	0/0	0/0	0/0	0/0	0/0	INTERGENIC(MODIFIER)
10'162'232	A	T	0/1	0/0	0/0	0/0	0/0	0/0	0/0	0/0	0/0	0/0	0/0	INTERGENIC(MODIFIER)
10'171'570	G	T	0/1	0/0	0/0	0/0	0/0	0/0	0/0	0/0	0/0	0/0	0/0	INTERGENIC(MODIFIER)
10'172'614	A	G	0/1	0/0	0/0	0/0	0/0	0/0	0/0	0/0	0/0	0/0	0/0	INTERGENIC(MODIFIER)
10'173'896	G	A	0/1	0/0	0/0	0/0	0/0	0/0	0/0	0/0	0/0	0/0	0/0	INTERGENIC(MODIFIER)
10'174'110	C	T	0/1	0/0	0/0	0/0	0/0	0/0	0/0	0/0	0/0	0/0	0/0	INTERGENIC(MODIFIER)
10'178'460	G	A	0/1	0/0	0/0	0/0	0/0	0/0	0/0	0/0	0/0	0/0	0/0	INTERGENIC(MODIFIER)
10'180'268	C	T	0/1	0/0	0/0	0/0	0/0	0/0	0/0	0/0	0/0	0/0	0/0	INTERGENIC(MODIFIER)
10'180'685	G	A	0/1	0/0	0/0	0/0	0/0	0/0	0/0	0/0	0/0	0/0	0/0	INTERGENIC(MODIFIER)
10'181'010	C	T	0/1	0/0	0/0	0/0	0/0	0/0	0/0	0/0	0/0	0/0	0/0	INTERGENIC(MODIFIER)
10'183'351	C	T	0/1	0/0	0/0	0/0	0/0	0/0	0/0	0/0	0/0	0/0	0/0	INTERGENIC(MODIFIER)
10'185'238	A	G	0/1	0/0	0/0	0/0	0/0	0/0	0/0	0/0	0/0	0/0	0/0	INTERGENIC(MODIFIER)
10'186'414	C	G	0/1	0/0	0/0	0/0	0/0	0/0	0/0	0/0	0/0	0/0	0/0	INTERGENIC(MODIFIER)
10'188'650	T	C	0/1	0/0	0/0	0/0	0/0	0/0	0/0	0/0	0/0	0/0	0/0	INTERGENIC(MODIFIER)
10'190'445	T	G	0/1	0/0	0/0	0/0	0/0	0/0	0/0	0/0	0/0	0/0	0/0	INTERGENIC(MODIFIER)
10'198'471	T	C	0/1	0/0	0/0	0/0	0/0	0/0	0/0	0/0	0/0	0/0	0/0	INTERGENIC(MODIFIER)
10'198'817	G	C	0/1	0/0	0/0	0/0	0/0	0/0	0/0	0/0	0/0	0/0	0/0	INTERGENIC(MODIFIER)
10'199'782	C	T	0/1	0/0	0/0	0/0	0/0	0/0	0/0	0/0	0/0	0/0	0/0	INTERGENIC(MODIFIER)
10'200'125	A	G	0/1	0/0	0/0	0/0	0/0	0/0	0/0	0/0	0/0	0/0	0/0	INTERGENIC(MODIFIER)
10'201'682	C	T	0/1	0/0	0/0	0/0	0/0	0/0	0/0	0/0	0/0	0/0	0/0	INTERGENIC(MODIFIER)
10'203'659	C	T	0/1	0/0	0/0	0/0	0/0	0/0	0/0	0/0	0/0	0/0	0/0	INTERGENIC(MODIFIER)
10'205'328	G	C	0/1	0/0	0/0	0/0	0/0	0/0	0/0	0/0	0/0	0/0	0/0	INTERGENIC(MODIFIER)
10'205'886	T	C	0/1	0/0	0/0	0/0	0/0	0/0	0/0	0/0	0/0	0/0	0/0	INTERGENIC(MODIFIER)
10'206'506	A	C	0/1	0/0	0/0	0/0	0/0	0/0	0/0	0/0	0/0	0/0	0/0	INTERGENIC(MODIFIER)
10'210'153	C	T	0/1	0/0	0/0	0/0	0/0	0/0	0/0	0/0	0/0	0/0	0/0	INTERGENIC(MODIFIER)
10'210'494	A	G	0/1	0/0	0/0	0/0	0/0	0/0	0/0	0/0	0/0	0/0	0/0	INTERGENIC(MODIFIER)
10'213'223	T	A	0/1	0/0	0/0	0/0	0/0	0/0	0/0	0/0	0/0	0/0	0/0	INTERGENIC(MODIFIER)
10'214'768	G	A	0/1	0/0	0/0	0/0	0/0	0/0	0/0	0/0	0/0	0/0	0/0	INTERGENIC(MODIFIER)
10'215'619	A	G	0/1	0/0	0/0	0/0	0/0	0/0	0/0	0/0	0/0	0/0	0/0	INTERGENIC(MODIFIER)
10'217'312	G	A	0/1	0/0	0/0	0/0	0/0	0/0	0/0	0/0	0/0	0/0	0/0	INTERGENIC(MODIFIER)
10'221'031	C	CA	0/1	0/0	0/0	0/0	0/0	0/0	0/0	0/0	0/0	0/0	0/0	INTERGENIC(MODIFIER)
10'221'708	T	C	0/1	0/0	0/0	0/0	0/0	0/0	0/0	0/0	0/0	0/0	0/0	INTERGENIC(MODIFIER)
10'225'718	CA	C	0/1	0/0	0/0	0/0	0/0	0/0	0/0	0/0	0/0	0/0	0/0	INTERGENIC(MODIFIER)
10'233'241	T	C	0/1	0/0	0/0	0/0	0/0	0/0	0/0	0/0	0/0	0/0	0/0	INTERGENIC(MODIFIER)
10'237'055	G	A	0/1	0/0	0/0	0/0	0/0	0/0	0/0	0/0	0/0	0/0	0/0	INTERGENIC(MODIFIER)
10'239'052	A	C	0/1	0/0	0/0	0/0	0/0	0/0	0/0	0/0	0/0	0/0	0/0	INTERGENIC(MODIFIER)
10'241'561	T	A	0/1	0/0	0/0	0/0	0/0	0/0	0/0	0/0	0/0	0/0	0/0	INTERGENIC(MODIFIER)
10'243'039	TAAA	T	0/1	0/0	0/0	0/0	0/0	0/0	0/0	0/0	0/0	0/0	0/0	INTERGENIC(MODIFIER)
10'247'545	A	G	0/1	0/0	0/0	0/0	0/0	0/0	0/0	0/0	0/0	0/0	0/0	INTERGENIC(MODIFIER)
10'248'762	C	T	0/1	0/0	0/0	0/0	0/0	0/0	0/0	0/0	0/0	0/0	0/0	INTERGENIC(MODIFIER)
10'262'255	C	T	0/1	0/0	0/0	0/0	0/0	0/0	0/0	0/0	0/0	0/0	0/0	INTERGENIC(MODIFIER)
10'267'473	C	T	0/1	0/0	0/0	0/0	0/0	0/0	0/0	0/0	0/0	0/0	0/0	INTERGENIC(MODIFIER)
10'270'151	C	A	0/1	0/0	0/0	0/0	0/0	0/0	0/0	0/0	0/0	0/0	0/0	INTERGENIC(MODIFIER)
10'274'255	C	T	0/1	0/0	0/0	0/0	0/0	0/0	0/0	0/0	0/0	0/0	0/0	INTERGENIC(MODIFIER)
10'282'571	C	T	0/1	0/0	0/0	0/0	0/0	0/0	0/0	0/0	0/0	0/0	0/0	INTERGENIC(MODIFIER)
10'287'335	C	G	0/1	0/0	0/0	0/0	0/0	0/0	0/0	0/0	0/0	0/0	0/0	INTERGENIC(MODIFIER)
10'290'295	A	G	0/1	0/0	0/0	0/0	0/0	0/0	0/0	0/0	0/0	0/0	0/0	INTERGENIC(MODIFIER)
10'298'355	A	G	0/1	0/0	0/0	0/0	0/0	0/0	0/0	0/0	0/0	0/0	0/0	INTERGENIC(MODIFIER),UPSTREAM(MODIFIER||1618||PRMT10||rna18475|)
10'304'348	G	C	0/1	0/0	0/0	0/0	0/0	0/0	0/0	0/0	0/0	0/0	0/0	INTRON(MODIFIER|||PRMT10||rna18475|2)
10'304'795	A	T	0/1	0/0	0/0	0/0	0/0	0/0	0/0	0/0	0/0	0/0	0/0	INTRON(MODIFIER|||PRMT10||rna18475|2)

10'309'145	T	C	0/1	0/0	0/0	0/0	0/0	0/0	0/0	0/0	0/0	0/0	0/0	INTRON(MODIFIER|||PRMT10||rna18475|5)
10'310'789	C	G	0/1	0/0	0/0	0/0	0/0	0/0	0/0	0/0	0/0	0/0	0/0	INTRON(MODIFIER|||PRMT10||rna18475|6)
10'313'362	G	A	0/1	0/0	0/0	0/0	0/0	0/0	0/0	0/0	0/0	0/0	0/0	INTRON(MODIFIER|||PRMT10||rna18475|6)
10'319'960	G	A	0/1	0/0	0/0	0/0	0/0	0/0	0/0	0/0	0/0	0/0	0/0	INTRON(MODIFIER|||PRMT10||rna18475|8)
10'322'518	C	T	0/1	0/0	0/0	0/0	0/0	0/0	0/0	0/0	0/0	0/0	0/0	INTRON(MODIFIER|||PRMT10||rna18475|9)
10'322'624	A	G	0/1	0/0	0/0	0/0	0/0	0/0	0/0	0/0	0/0	0/0	0/0	INTRON(MODIFIER|||PRMT10||rna18475|9)
10'322'905	A	G	0/1	0/0	0/0	0/0	0/0	0/0	0/0	0/0	0/0	0/0	0/0	INTRON(MODIFIER|||PRMT10||rna18475|9)
10'324'561	A	G	0/1	0/0	0/0	0/0	0/0	0/0	0/0	0/0	0/0	0/0	0/0	INTRON(MODIFIER|||PRMT10||rna18475|9)
10'324'705	A	G	0/1	0/0	0/0	0/0	0/0	0/0	0/0	0/0	0/0	0/0	0/0	INTRON(MODIFIER|||PRMT10||rna18475|9)
10'325'024	A	G	0/1	0/0	0/0	0/0	0/0	0/0	0/0	0/0	0/0	0/0	0/0	INTRON(MODIFIER|||PRMT10||rna18475|9)
10'325'232	C	CTCTT	0/1	0/0	0/0	0/0	0/0	0/0	0/0	0/0	0/0	0/0	0/0	INTRON(MODIFIER|||PRMT10||rna18475|9)
10'325'245	C	G	0/1	0/0	0/0	0/0	0/0	0/0	0/0	0/0	0/0	0/0	0/0	INTRON(MODIFIER|||PRMT10||rna18475|9)
10'325'327	T	C	0/1	0/0	0/0	0/0	0/0	0/0	0/0	0/0	0/0	0/0	0/0	INTRON(MODIFIER|||PRMT10||rna18475|9)
10'325'352	C	T	0/1	0/0	0/0	0/0	0/0	0/0	0/0	0/0	0/0	0/0	0/0	INTRON(MODIFIER|||PRMT10||rna18475|9)
10'326'017	T	G	0/1	0/0	0/0	0/0	0/0	0/0	0/0	0/0	0/0	0/0	0/0	INTRON(MODIFIER|||PRMT10||rna18475|9)
10'326'250	A	G	0/1	0/0	0/0	0/0	0/0	0/0	0/0	0/0	0/0	0/0	0/0	INTRON(MODIFIER|||PRMT10||rna18475|9)
10'326'593	A	T	0/1	0/0	0/0	0/0	0/0	0/0	0/0	0/0	0/0	0/0	0/0	INTRON(MODIFIER|||PRMT10||rna18475|9)
10'326'895	G	A	0/1	0/0	0/0	0/0	0/0	0/0	0/0	0/0	0/0	0/0	0/0	INTRON(MODIFIER|||PRMT10||rna18475|9)
10'327'982	T	C	0/1	0/0	0/0	0/0	0/0	0/0	0/0	0/0	0/0	0/0	0/0	INTRON(MODIFIER|||PRMT10||rna18475|10)
10'328'162	G	A	0/1	0/0	0/0	0/0	0/0	0/0	0/0	0/0	0/0	0/0	0/0	INTRON(MODIFIER|||PRMT10||rna18475|10)
10'328'421	G	A	0/1	0/0	0/0	0/0	0/0	0/0	0/0	0/0	0/0	0/0	0/0	INTRON(MODIFIER|||PRMT10||rna18475|10)
10'328'538	G	T	0/1	0/0	0/0	0/0	0/0	0/0	0/0	0/0	0/0	0/0	0/0	INTRON(MODIFIER|||PRMT10||rna18475|10)
10'328'688	G	GTA	0/1	0/0	0/0	0/0	0/0	0/0	0/0	0/0	0/0	0/0	0/0	INTRON(MODIFIER|||PRMT10||rna18475|10)
10'329'446	C	G	0/1	0/0	0/0	0/0	0/0	0/0	0/0	0/0	0/0	0/0	0/0	INTRON(MODIFIER|||PRMT10||rna18475|10)
10'330'173	A	G	0/1	0/0	0/0	0/0	0/0	0/0	0/0	0/0	0/0	0/0	0/0	INTRON(MODIFIER|||PRMT10||rna18475|10)
10'333'539	G	A	0/1	0/0	0/0	0/0	0/0	0/0	0/0	0/0	0/0	0/0	0/0	DOWNSTREAM(MODIFIER||2619||TMEM184C||rna18476|),INTRON(MODIFIER|||PRMT10||rna18475|10)
10'334'419	A	G	0/1	0/0	0/0	0/0	0/0	0/0	0/0	0/0	0/0	0/0	0/0	DOWNSTREAM(MODIFIER||1739||TMEM184C||rna18476|),INTRON(MODIFIER|||PRMT10||rna18475|11)
10'335'563	T	C	0/1	0/0	0/0	0/0	0/0	0/0	0/0	0/0	0/0	0/0	0/0	DOWNSTREAM(MODIFIER||2||PRMT10||rna18475|),DOWNSTREAM(MODIFIER||595||TMEM184C||rna18476|),INTERGENIC(MODIFIER)
10'335'904	C	G	0/1	0/0	0/0	0/0	0/0	0/0	0/0	0/0	0/0	0/0	0/0	DOWNSTREAM(MODIFIER||254||TMEM184C||rna18476|),DOWNSTREAM(MODIFIER||343||PRMT10||rna18475|),INTERGENIC(MODIFIER)
10'360'217	G	C	0/1	0/0	0/0	0/0	0/0	0/0	0/0	0/0	0/0	0/0	0/0	NON_SYNONYMOUS_CODING(MODERATE|MISSENSE|cCt/cGt|P77R|TMEM184C||rna18476|2)
10'370'533	TGATTATGC	T	0/1	0/0	0/0	0/0	0/0	0/0	0/0	0/0	0/0	0/0	0/0	INTERGENIC(MODIFIER),UPSTREAM(MODIFIER||2153||TMEM184C||rna18476|)
10'389'883	T	C	0/1	0/0	0/0	0/0	0/0	0/0	0/0	0/0	0/0	0/0	0/0	INTERGENIC(MODIFIER)
10'424'379	A	G	0/1	0/0	0/0	0/0	0/0	0/0	0/0	0/0	0/0	0/0	0/0	INTERGENIC(MODIFIER)
10'425'339	G	T	0/1	0/0	0/0	0/0	0/0	0/0	0/0	0/0	0/0	0/0	0/0	INTERGENIC(MODIFIER)
10'440'403	GGAGA	G	0/1	0/0	0/0	0/0	0/0	0/0	0/0	0/0	0/0	0/0	0/0	DOWNSTREAM(MODIFIER||3074||EDNRA||rna18477|),DOWNSTREAM(MODIFIER||3074||EDNRA||rna18478|),INTERGENIC(MODIFIER)
10'441'502	T	C	0/1	0/0	0/0	0/0	0/0	0/0	0/0	0/0	0/0	0/0	0/0	DOWNSTREAM(MODIFIER||1976||EDNRA||rna18477|),DOWNSTREAM(MODIFIER||1976||EDNRA||rna18478|),INTERGENIC(MODIFIER)
10'441'541	A	G	0/1	0/0	0/0	0/0	0/0	0/0	0/0	0/0	0/0	0/0	0/0	DOWNSTREAM(MODIFIER||1937||EDNRA||rna18477|),DOWNSTREAM(MODIFIER||1937||EDNRA||rna18478|),INTERGENIC(MODIFIER)
10'441'586	C	T	0/1	0/0	0/0	0/0	0/0	0/0	0/0	0/0	0/0	0/0	0/0	DOWNSTREAM(MODIFIER||1892||EDNRA||rna18477|),DOWNSTREAM(MODIFIER||1892||EDNRA||rna18478|),INTERGENIC(MODIFIER)
10'442'242	T	C	0/1	0/0	0/0	0/0	0/0	0/0	0/0	0/0	0/0	0/0	0/0	DOWNSTREAM(MODIFIER||1236||EDNRA||rna18477|),DOWNSTREAM(MODIFIER||1236||EDNRA||rna18478|),INTERGENIC(MODIFIER)
10'442'675	T	C	0/1	0/0	0/0	0/0	0/0	0/0	0/0	0/0	0/0	0/0	0/0	DOWNSTREAM(MODIFIER||803||EDNRA||rna18477|),DOWNSTREAM(MODIFIER||803||EDNRA||rna18478|),INTERGENIC(MODIFIER)
10'443'075	C	T	0/1	0/0	0/0	0/0	0/0	0/0	0/0	0/0	0/0	0/0	0/0	DOWNSTREAM(MODIFIER||403||EDNRA||rna18477|),DOWNSTREAM(MODIFIER||403||EDNRA||rna18478|),INTERGENIC(MODIFIER)
10'443'116	CATGTATAT	C	0/1	0/0	0/0	0/0	0/0	0/0	0/0	0/0	0/0	0/0	0/0	DOWNSTREAM(MODIFIER||361||EDNRA||rna18477|),DOWNSTREAM(MODIFIER||361||EDNRA||rna18478|),INTERGENIC(MODIFIER)
10'444'158	G	A	0/1	0/0	0/0	0/0	0/0	0/0	0/0	0/0	0/0	0/0	0/0	UTR_3_PRIME(MODIFIER|||EDNRA||rna18477|5),UTR_3_PRIME(MODIFIER|||EDNRA||rna18478|7)
10'444'567	G	A	0/1	0/0	0/0	0/0	0/0	0/0	0/0	0/0	0/0	0/0	0/0	INTRON(MODIFIER|||EDNRA||rna18477|4),INTRON(MODIFIER|||EDNRA||rna18478|6)
10'444'984	C	T	0/1	0/0	0/0	0/0	0/0	0/0	0/0	0/0	0/0	0/0	0/0	INTRON(MODIFIER|||EDNRA||rna18477|4),INTRON(MODIFIER|||EDNRA||rna18478|6)
10'444'995	TC	T	0/1	0/0	0/0	0/0	0/0	0/0	0/0	0/0	0/0	0/0	0/0	INTRON(MODIFIER|||EDNRA||rna18477|4),INTRON(MODIFIER|||EDNRA||rna18478|6)
10'447'400	G	A	0/1	0/0	0/0	0/0	0/0	0/0	0/0	0/0	0/0	0/0	0/0	INTRON(MODIFIER|||EDNRA||rna18477|4),INTRON(MODIFIER|||EDNRA||rna18478|6)
10'447'434	G	A	0/1	0/0	0/0	0/0	0/0	0/0	0/0	0/0	0/0	0/0	0/0	INTRON(MODIFIER|||EDNRA||rna18477|4),INTRON(MODIFIER|||EDNRA||rna18478|6)
10'447'777	C	T	0/1	0/0	0/0	0/0	0/0	0/0	0/0	0/0	0/0	0/0	0/0	INTRON(MODIFIER|||EDNRA||rna18477|4),INTRON(MODIFIER|||EDNRA||rna18478|6)
10'448'163	C	A	0/1	0/0	0/0	0/0	0/0	0/0	0/0	0/0	0/0	0/0	0/0	INTRON(MODIFIER|||EDNRA||rna18477|4),INTRON(MODIFIER|||EDNRA||rna18478|6)
10'450'231	A	T	0/1	0/0	0/0	0/0	0/0	0/0	0/0	0/0	0/0	0/0	0/0	INTRON(MODIFIER|||EDNRA||rna18477|2),INTRON(MODIFIER|||EDNRA||rna18478|4)
10'450'509	T	C	0/1	0/0	0/0	0/0	0/0	0/0	0/0	0/0	0/0	0/0	0/0	INTRON(MODIFIER|||EDNRA||rna18477|2),INTRON(MODIFIER|||EDNRA||rna18478|4)
10'451'140	C	G	0/1	0/0	0/0	0/0	0/0	0/0	0/0	0/0	0/0	0/0	0/0	INTRON(MODIFIER|||EDNRA||rna18477|2),INTRON(MODIFIER|||EDNRA||rna18478|4)
10'452'593	GC	G	0/1	0/0	0/0	0/0	0/0	0/0	0/0	0/0	0/0	0/0	0/0	INTRON(MODIFIER|||EDNRA||rna18477|2),INTRON(MODIFIER|||EDNRA||rna18478|4)
10'453'699	A	G	0/1	0/0	0/0	0/0	0/0	0/0	0/0	0/0	0/0	0/0	0/0	INTRON(MODIFIER|||EDNRA||rna18477|2),INTRON(MODIFIER|||EDNRA||rna18478|4)
10'459'860	G	A	0/1	0/0	0/0	0/0	0/0	0/0	0/0	0/0	0/0	0/0	0/0	INTRON(MODIFIER|||EDNRA||rna18477|1),INTRON(MODIFIER|||EDNRA||rna18478|3)
10'463'586	T	C	0/1	0/0	0/0	0/0	0/0	0/0	0/0	0/0	0/0	0/0	0/0	INTRON(MODIFIER|||EDNRA||rna18477|1),INTRON(MODIFIER|||EDNRA||rna18478|2)
10'466'953	A	G	1/1	0/0	0/0	0/0	0/0	0/0	0/0	0/0	0/0	0/0	0/0	INTRON(MODIFIER|||EDNRA||rna18477|1),INTRON(MODIFIER|||EDNRA||rna18478|2)
10'471'257	G	A	0/1	0/0	0/0	0/0	0/0	0/0	0/0	0/0	0/0	0/0	0/0	INTRON(MODIFIER|||EDNRA||rna18477|1),INTRON(MODIFIER|||EDNRA||rna18478|2)
10'471'562	C	T	1/1	0/0	0/0	0/0	0/0	0/0	0/0	0/0	0/0	0/0	0/0	INTRON(MODIFIER|||EDNRA||rna18477|1),INTRON(MODIFIER|||EDNRA||rna18478|2)
10'473'164	A	G	1/1	0/0	0/0	0/0	0/0	0/0	0/0	0/0	0/0	0/0	0/0	INTRON(MODIFIER|||EDNRA||rna18477|1),INTRON(MODIFIER|||EDNRA||rna18478|2)
10'473'628	T	C	0/1	0/0	0/0	0/0	0/0	0/0	0/0	0/0	0/0	0/0	0/0	INTRON(MODIFIER|||EDNRA||rna18477|1),INTRON(MODIFIER|||EDNRA||rna18478|2)
10'475'655	T	C	0/1	0/0	0/0	0/0	0/0	0/0	0/0	0/0	0/0	0/0	0/0	INTRON(MODIFIER|||EDNRA||rna18477|1),INTRON(MODIFIER|||EDNRA||rna18478|2)
10'478'122	T	C	0/1	0/0	0/0	0/0	0/0	0/0	0/0	0/0	0/0	0/0	0/0	INTRON(MODIFIER|||EDNRA||rna18477|1),INTRON(MODIFIER|||EDNRA||rna18478|1)
10'478'424	C	T	0/1	0/0	0/0	0/0	0/0	0/0	0/0	0/0	0/0	0/0	0/0	INTRON(MODIFIER|||EDNRA||rna18477|1),INTRON(MODIFIER|||EDNRA||rna18478|1)
10'480'794	G	A	0/1	0/0	0/0	0/0	0/0	0/0	0/0	0/0	0/0	0/0	0/0	INTRON(MODIFIER|||EDNRA||rna18477|1),INTRON(MODIFIER|||EDNRA||rna18478|1)
10'482'146	G	A	0/1	0/0	0/0	0/0	0/0	0/0	0/0	0/0	0/0	0/0	0/0	INTRON(MODIFIER|||EDNRA||rna18477|1),INTRON(MODIFIER|||EDNRA||rna18478|1)
10'483'125	T	C	0/1	0/0	0/0	0/0	0/0	0/0	0/0	0/0	0/0	0/0	0/0	INTRON(MODIFIER|||EDNRA||rna18477|1),INTRON(MODIFIER|||EDNRA||rna18478|1)
10'485'551	C	A	0/1	0/0	0/0	0/0	0/0	0/0	0/0	0/0	0/0	0/0	0/0	INTRON(MODIFIER|||EDNRA||rna18477|1),INTRON(MODIFIER|||EDNRA||rna18478|1)
10'487'939	T	C	0/1	0/0	0/0	0/0	0/0	0/0	0/0	0/0	0/0	0/0	0/0	INTRON(MODIFIER|||EDNRA||rna18477|1),INTRON(MODIFIER|||EDNRA||rna18478|1)
10'492'132	C	T	0/1	0/0	0/0	0/0	0/0	0/0	0/0	0/0	0/0	0/0	0/0	INTRON(MODIFIER|||EDNRA||rna18477|1),INTRON(MODIFIER|||EDNRA||rna18478|1)
10'493'920	A	G	0/1	0/0	0/0	0/0	0/0	0/0	0/0	0/0	0/0	0/0	0/0	INTRON(MODIFIER|||EDNRA||rna18477|1),INTRON(MODIFIER|||EDNRA||rna18478|1)
10'497'667	T	C	0/1	0/0	0/0	0/0	0/0	0/0	0/0	0/0	0/0	0/0	0/0	INTRON(MODIFIER|||EDNRA||rna18477|1),INTRON(MODIFIER|||EDNRA||rna18478|1)
10'501'389	A	G	0/1	0/0	0/0	0/0	0/0	0/0	0/0	0/0	0/0	0/0	0/0	INTRON(MODIFIER|||EDNRA||rna18477|1),INTRON(MODIFIER|||EDNRA||rna18478|1)
10'506'880	C	T	0/1	0/0	0/0	0/0	0/0	0/0	0/0	0/0	0/0	0/0	0/0	INTRON(MODIFIER|||EDNRA||rna18477|1),INTRON(MODIFIER|||EDNRA||rna18478|1)
10'511'253	A	G	0/1	0/0	0/0	0/0	0/0	0/0	0/0	0/0	0/0	0/0	0/0	NON_SYNONYMOUS_CODING(MODERATE|MISSENSE|Tat/Cat|Y129H|EDNRA||rna18477|1),NON_SYNONYMOUS_CODING(MODERATE|MISSENSE|Tat/Cat|Y129H|EDNRA||rna18478|1)
10'544'494	A	G	0/1	0/0	0/0	0/0	0/0	0/0	0/0	0/0	0/0	0/0	0/0	INTERGENIC(MODIFIER)
10'546'288	G	A	0/1	0/0	0/0	0/0	0/0	0/0	0/0	0/0	0/0	0/0	0/0	INTERGENIC(MODIFIER)
10'547'475	C	A	0/1	0/0	0/0	0/0	0/0	0/0	0/0	0/0	0/0	0/0	0/0	INTERGENIC(MODIFIER)
10'553'242	A	G	0/1	0/0	0/0	0/0	0/0	0/0	0/0	0/0	0/0	0/0	0/0	INTERGENIC(MODIFIER)
10'554'680	C	A	0/1	0/0	0/0	0/0	0/0	0/0	0/0	0/0	0/0	0/0	0/0	INTERGENIC(MODIFIER)
10'558'003	A	G	0/1	0/0	0/0	0/0	0/0	0/0	0/0	0/0	0/0	0/0	0/0	INTERGENIC(MODIFIER)
10'558'674	A	C	0/1	0/0	0/0	0/0	0/0	0/0	0/0	0/0	0/0	0/0	0/0	INTERGENIC(MODIFIER)
10'560'363	A	G	0/1	0/0	0/0	0/0	0/0	0/0	0/0	0/0	0/0	0/0	0/0	INTERGENIC(MODIFIER)
10'562'308	T	TG	1/1	0/0	0/0	0/0	0/0	0/0	0/0	0/0	0/0	0/0	0/0	INTERGENIC(MODIFIER)
10'562'413	G	A	0/1	0/0	0/0	0/0	0/0	0/0	0/0	0/0	0/0	0/0	0/0	INTERGENIC(MODIFIER)
10'563'449	T	G	0/1	0/0	0/0	0/0	0/0	0/0	0/0	0/0	0/0	0/0	0/0	INTERGENIC(MODIFIER)
10'563'583	CTCTGTTTTA	C	0/1	0/0	0/0	0/0	0/0	0/0	0/0	0/0	0/0	0/0	0/0	INTERGENIC(MODIFIER)
10'563'774	GTCTGCCA	G	0/1	0/0	0/0	0/0	0/0	0/0	0/0	0/0	0/0	0/0	0/0	INTERGENIC(MODIFIER)
10'564'935	T	C	0/1	0/0	0/0	0/0	0/0	0/0	0/0	0/0	0/0	0/0	0/0	INTERGENIC(MODIFIER)
10'568'154	A	G	0/1	0/0	0/0	0/0	0/0	0/0	0/0	0/0	0/0	0/0	0/0	INTERGENIC(MODIFIER)
10'572'662	T	A	0/1	0/0	0/0	0/0	0/0	0/0	0/0	0/0	0/0	0/0	0/0	INTERGENIC(MODIFIER)
10'574'784	A	G	0/1	0/0	0/0	0/0	0/0	0/0	0/0	0/0	0/0	0/0	0/0	INTERGENIC(MODIFIER)
10'578'137	TTG	T	0/1	0/0	0/0	0/0	0/0	0/0	0/0	0/0	0/0	0/0	0/0	INTERGENIC(MODIFIER)
10'578'496	G	T	0/1	0/0	0/0	0/0	0/0	0/0	0/0	0/0	0/0	0/0	0/0	INTERGENIC(MODIFIER)
10'578'696	A	G	0/1	0/0	0/0	0/0	0/0	0/0	0/0	0/0	0/0	0/0	0/0	INTERGENIC(MODIFIER)
10'578'746	C	A	0/1	0/0	0/0	0/0	0/0	0/0	0/0	0/0	0/0	0/0	0/0	INTERGENIC(MODIFIER)
10'578'783	T	C	0/1	0/0	0/0	0/0	0/0	0/0	0/0	0/0	0/0	0/0	0/0	INTERGENIC(MODIFIER)
10'578'848	TA	T	0/1	0/0	0/0	0/0	0/0	0/0	0/0	0/0	0/0	0/0	0/0	INTERGENIC(MODIFIER)
10'578'974	T	C	0/1	0/0	0/0	0/0	0/0	0/0	0/0	0/0	0/0	0/0	0/0	INTERGENIC(MODIFIER)
10'578'983	G	A	0/1	0/0	0/0	0/0	0/0	0/0	0/0	0/0	0/0	0/0	0/0	INTERGENIC(MODIFIER)
10'578'992	T	C	0/1	0/0	0/0	0/0	0/0	0/0	0/0	0/0	0/0	0/0	0/0	INTERGENIC(MODIFIER)
10'579'157	C	CA	0/1	0/0	0/0	0/0	0/0	0/0	0/0	0/0	0/0	0/0	0/0	INTERGENIC(MODIFIER)
10'579'301	G	A	0/1	0/0	0/0	0/0	0/0	0/0	0/0	0/0	0/0	0/0	0/0	INTERGENIC(MODIFIER)
10'579'311	C	A	0/1	0/0	0/0	0/0	0/0	0/0	0/0	0/0	0/0	0/0	0/0	INTERGENIC(MODIFIER)
10'579'682	A	G	0/1	0/0	0/0	0/0	0/0	0/0	0/0	0/0	0/0	0/0	0/0	INTERGENIC(MODIFIER)
10'579'725	G	C	0/1	0/0	0/0	0/0	0/0	0/0	0/0	0/0	0/0	0/0	0/0	INTERGENIC(MODIFIER)
10'579'740	A	G	0/1	0/0	0/0	0/0	0/0	0/0	0/0	0/0	0/0	0/0	0/0	INTERGENIC(MODIFIER)
10'579'925	G	A	0/1	0/0	0/0	0/0	0/0	0/0	0/0	0/0	0/0	0/0	0/0	INTERGENIC(MODIFIER)
10'579'989	C	T	0/1	0/0	0/0	0/0	0/0	0/0	0/0	0/0	0/0	0/0	0/0	INTERGENIC(MODIFIER)
10'581'810	T	C	0/1	0/0	0/0	0/0	0/0	0/0	0/0	0/0	0/0	0/0	0/0	INTERGENIC(MODIFIER)
10'582'198	G	A	0/1	0/0	0/0	0/0	0/0	0/0	0/0	0/0	0/0	0/0	0/0	INTERGENIC(MODIFIER)
10'582'786	C	G	0/1	0/0	0/0	0/0	0/0	0/0	0/0	0/0	0/0	0/0	0/0	INTERGENIC(MODIFIER)
10'583'559	G	A	0/1	0/0	0/0	0/0	0/0	0/0	0/0	0/0	0/0	0/0	0/0	INTERGENIC(MODIFIER)
10'584'793	T	C	0/1	0/0	0/0	0/0	0/0	0/0	0/0	0/0	0/0	0/0	0/0	INTERGENIC(MODIFIER)
10'585'900	T	A	0/1	0/0	0/0	0/0	0/0	0/0	0/0	0/0	0/0	0/0	0/0	INTERGENIC(MODIFIER)
10'586'292	A	T	0/1	0/0	0/0	0/0	0/0	0/0	0/0	0/0	0/0	0/0	0/0	INTERGENIC(MODIFIER)
10'586'410	G	A	0/1	0/0	0/0	0/0	0/0	0/0	0/0	0/0	0/0	0/0	0/0	INTERGENIC(MODIFIER)
10'589'638	A	G	0/1	0/0	0/0	0/0	0/0	0/0	0/0	0/0	0/0	0/0	0/0	INTERGENIC(MODIFIER)
10'590'290	A	G	0/1	0/0	0/0	0/0	0/0	0/0	0/0	0/0	0/0	0/0	0/0	INTERGENIC(MODIFIER)
10'600'055	A	C	0/1	0/0	0/0	0/0	0/0	0/0	0/0	0/0	0/0	0/0	0/0	INTERGENIC(MODIFIER)
10'600'613	CTG	C	0/1	0/0	0/0	0/0	0/0	0/0	0/0	0/0	0/0	0/0	0/0	INTERGENIC(MODIFIER)
10'601'888	C	T	0/1	0/0	0/0	0/0	0/0	0/0	0/0	0/0	0/0	0/0	0/0	INTERGENIC(MODIFIER)
10'602'506	GA	G	0/1	0/0	0/0	0/0	0/0	0/0	0/0	0/0	0/0	0/0	0/0	INTERGENIC(MODIFIER)
10'605'719	A	AC	0/1	0/0	0/0	0/0	0/0	0/0	0/0	0/0	0/0	0/0	0/0	INTERGENIC(MODIFIER)
10'609'559	A	T	0/1	0/0	0/0	0/0	0/0	0/0	0/0	0/0	0/0	0/0	0/0	INTERGENIC(MODIFIER)
10'610'214	TG	T	0/1	0/0	0/0	0/0	0/0	0/0	0/0	0/0	0/0	0/0	0/0	INTERGENIC(MODIFIER)
10'612'106	G	A	0/1	0/0	0/0	0/0	0/0	0/0	0/0	0/0	0/0	0/0	0/0	INTERGENIC(MODIFIER)
10'616'724	C	T	0/1	0/0	0/0	0/0	0/0	0/0	0/0	0/0	0/0	0/0	0/0	INTERGENIC(MODIFIER)
10'617'647	T	G	0/1	0/0	0/0	0/0	0/0	0/0	0/0	0/0	0/0	0/0	0/0	INTERGENIC(MODIFIER)
10'620'615	T	G	0/1	0/0	0/0	0/0	0/0	0/0	0/0	0/0	0/0	0/0	0/0	INTERGENIC(MODIFIER)
10'626'591	A	G	0/1	0/0	0/0	0/0	0/0	0/0	0/0	0/0	0/0	0/0	0/0	INTERGENIC(MODIFIER)
10'626'995	T	G	0/1	0/0	0/0	0/0	0/0	0/0	0/0	0/0	0/0	0/0	0/0	INTERGENIC(MODIFIER)
10'627'206	C	T	0/1	0/0	0/0	0/0	0/0	0/0	0/0	0/0	0/0	0/0	0/0	INTERGENIC(MODIFIER)
10'630'524	T	C	0/1	0/0	0/0	0/0	0/0	0/0	0/0	0/0	0/0	0/0	0/0	INTERGENIC(MODIFIER)
10'632'427	C	A	0/1	0/0	0/0	0/0	0/0	0/0	0/0	0/0	0/0	0/0	0/0	INTERGENIC(MODIFIER)
10'632'465	C	T	0/1	0/0	0/0	0/0	0/0	0/0	0/0	0/0	0/0	0/0	0/0	INTERGENIC(MODIFIER)
10'632'780	C	CAAAT	0/1	0/0	0/0	0/0	0/0	0/0	0/0	0/0	0/0	0/0	0/0	INTERGENIC(MODIFIER)
10'632'797	T	C	0/1	0/0	0/0	0/0	0/0	0/0	0/0	0/0	0/0	0/0	0/0	INTERGENIC(MODIFIER)
10'632'913	G	A	0/1	0/0	0/0	0/0	0/0	0/0	0/0	0/0	0/0	0/0	0/0	INTERGENIC(MODIFIER)
10'635'959	A	G	0/1	0/0	0/0	0/0	0/0	0/0	0/0	0/0	0/0	0/0	0/0	INTERGENIC(MODIFIER)
10'642'090	A	G	0/1	0/0	0/0	0/0	0/0	0/0	0/0	0/0	0/0	0/0	0/0	INTERGENIC(MODIFIER)
10'644'441	C	T	0/1	0/0	0/0	0/0	0/0	0/0	0/0	0/0	0/0	0/0	0/0	INTERGENIC(MODIFIER)

10'645'025	C	T	0/1	0/0	0/0	0/0	0/0	0/0	0/0	0/0	0/0	0/0	0/0	INTERGENIC(MODIFIER)
10'646'060	ATCCCATTT	A	0/1	0/0	0/0	0/0	0/0	0/0	0/0	0/0	0/0	0/0	0/0	INTERGENIC(MODIFIER)
10'646'323	C	A	0/1	0/0	0/0	0/0	0/0	0/0	0/0	0/0	0/0	0/0	0/0	INTERGENIC(MODIFIER)
10'648'223	A	T	0/1	0/0	0/0	0/0	0/0	0/0	0/0	0/0	0/0	0/0	0/0	INTERGENIC(MODIFIER)
10'649'153	G	C	0/1	0/0	0/0	0/0	0/0	0/0	0/0	0/0	0/0	0/0	0/0	INTERGENIC(MODIFIER)
10'649'175	A	C	0/1	0/0	0/0	0/0	0/0	0/0	0/0	0/0	0/0	0/0	0/0	INTERGENIC(MODIFIER)
10'650'958	A	G	0/1	0/0	0/0	0/0	0/0	0/0	0/0	0/0	0/0	0/0	0/0	INTERGENIC(MODIFIER)
10'652'048	C	CCAT	0/1	0/0	0/0	0/0	0/0	0/0	0/0	0/0	0/0	0/0	0/0	INTERGENIC(MODIFIER)
10'653'194	C	T	0/1	0/0	0/0	0/0	0/0	0/0	0/0	0/0	0/0	0/0	0/0	INTERGENIC(MODIFIER)
10'653'214	C	T	0/1	0/0	0/0	0/0	0/0	0/0	0/0	0/0	0/0	0/0	0/0	INTERGENIC(MODIFIER)
10'654'684	T	A	0/1	0/0	0/0	0/0	0/0	0/0	0/0	0/0	0/0	0/0	0/0	INTERGENIC(MODIFIER)
10'655'315	A	G	0/1	0/0	0/0	0/0	0/0	0/0	0/0	0/0	0/0	0/0	0/0	INTERGENIC(MODIFIER)
10'655'506	T	G	0/1	0/0	0/0	0/0	0/0	0/0	0/0	0/0	0/0	0/0	0/0	INTERGENIC(MODIFIER)
10'658'984	C	T	0/1	0/0	0/0	0/0	0/0	0/0	0/0	0/0	0/0	0/0	0/0	INTERGENIC(MODIFIER)
10'659'710	A	G	0/1	0/0	0/0	0/0	0/0	0/0	0/0	0/0	0/0	0/0	0/0	INTERGENIC(MODIFIER)
10'661'060	A	G	0/1	0/0	0/0	0/0	0/0	0/0	0/0	0/0	0/0	0/0	0/0	INTERGENIC(MODIFIER)
10'666'156	T	C	0/1	0/0	0/0	0/0	0/0	0/0	0/0	0/0	0/0	0/0	0/0	INTERGENIC(MODIFIER)
10'677'490	G	C	0/1	0/0	0/0	0/0	0/0	0/0	0/0	0/0	0/0	0/0	0/0	INTERGENIC(MODIFIER)
10'679'225	A	C	0/1	0/0	0/0	0/0	0/0	0/0	0/0	0/0	0/0	0/0	0/0	INTERGENIC(MODIFIER)
10'682'380	G	A	0/1	0/0	0/0	0/0	0/0	0/0	0/0	0/0	0/0	0/0	0/0	INTERGENIC(MODIFIER)
10'682'608	A	C	0/1	0/0	0/0	0/0	0/0	0/0	0/0	0/0	0/0	0/0	0/0	INTERGENIC(MODIFIER)
10'682'695	G	T	0/1	0/0	0/0	0/0	0/0	0/0	0/0	0/0	0/0	0/0	0/0	INTERGENIC(MODIFIER)
10'691'521	T	C	0/1	0/0	0/0	0/0	0/0	0/0	0/0	0/0	0/0	0/0	0/0	INTERGENIC(MODIFIER)
10'693'296	G	A	0/1	0/0	0/0	0/0	0/0	0/0	0/0	0/0	0/0	0/0	0/0	INTERGENIC(MODIFIER)
10'698'866	T	A	0/1	0/0	0/0	0/0	./.	0/0	0/0	0/0	0/0	0/0	0/0	INTERGENIC(MODIFIER)
10'699'029	C	T	0/1	0/0	0/0	0/0	./.	0/0	0/0	0/0	0/0	0/0	0/0	INTERGENIC(MODIFIER)
10'700'569	C	A	0/1	0/0	0/0	0/0	0/0	0/0	0/0	0/0	0/0	0/0	0/0	INTERGENIC(MODIFIER)
10'700'920	A	T	0/1	0/0	0/0	0/0	0/0	0/0	0/0	0/0	0/0	0/0	0/0	INTERGENIC(MODIFIER)
10'702'442	G	A	0/1	0/0	0/0	0/0	0/0	0/0	0/0	0/0	0/0	0/0	0/0	INTERGENIC(MODIFIER)
10'703'083	G	A	0/1	0/0	0/0	0/0	0/0	0/0	0/0	0/0	0/0	0/0	0/0	INTERGENIC(MODIFIER)
10'709'432	T	C	0/1	0/0	0/0	0/0	0/0	0/0	0/0	0/0	0/0	0/0	0/0	INTERGENIC(MODIFIER)
10'709'641	G	A	0/1	0/0	0/0	0/0	0/0	0/0	0/0	0/0	0/0	0/0	0/0	INTERGENIC(MODIFIER)
10'709'891	G	A	0/1	0/0	0/0	0/0	0/0	0/0	0/0	0/0	0/0	0/0	0/0	INTERGENIC(MODIFIER)
10'709'999	A	G	0/1	0/0	0/0	0/0	0/0	0/0	0/0	0/0	0/0	0/0	0/0	INTERGENIC(MODIFIER)
10'710'005	G	A	0/1	0/0	0/0	0/0	0/0	0/0	0/0	0/0	0/0	0/0	0/0	INTERGENIC(MODIFIER)
10'710'054	T	C	0/1	0/0	0/0	0/0	0/0	0/0	0/0	0/0	0/0	0/0	0/0	INTERGENIC(MODIFIER)
10'710'460	G	T	0/1	0/0	0/0	0/0	0/0	0/0	0/0	0/0	0/0	0/0	0/0	INTERGENIC(MODIFIER)
10'710'628	G	A	0/1	0/0	0/0	0/0	0/0	0/0	0/0	0/0	0/0	0/0	0/0	INTERGENIC(MODIFIER)
10'710'749	A	G	0/1	0/0	0/0	0/0	0/0	0/0	0/0	0/0	0/0	0/0	0/0	INTERGENIC(MODIFIER)
10'712'078	G	A	0/1	0/0	0/0	0/0	0/0	0/0	0/0	0/0	0/0	0/0	0/0	INTERGENIC(MODIFIER)
10'713'405	A	G	0/1	0/0	0/0	0/0	0/0	0/0	0/0	0/0	0/0	0/0	0/0	INTERGENIC(MODIFIER)
10'715'068	A	T	0/1	0/0	0/0	0/0	0/0	0/0	0/0	0/0	0/0	0/0	0/0	INTERGENIC(MODIFIER)
10'716'650	G	A	0/1	0/0	0/0	0/0	0/0	0/0	0/0	0/0	0/0	0/0	0/0	INTERGENIC(MODIFIER)
10'723'486	A	G	0/1	0/0	0/0	0/0	0/0	0/0	0/0	0/0	0/0	0/0	0/0	INTERGENIC(MODIFIER)
10'733'466	T	A	0/1	0/0	0/0	0/0	0/0	0/0	0/0	0/0	0/0	0/0	0/0	INTERGENIC(MODIFIER)
10'738'589	A	G	0/1	0/0	0/0	0/0	0/0	0/0	0/0	0/0	0/0	0/0	0/0	INTERGENIC(MODIFIER)
10'740'925	T	C	1/1	0/0	0/0	0/0	0/0	0/0	0/0	0/0	0/0	0/0	0/0	INTERGENIC(MODIFIER)
10'741'416	A	G	0/1	0/0	0/0	0/0	0/0	0/0	0/0	0/0	0/0	0/0	0/0	INTERGENIC(MODIFIER)
10'746'156	A	T	0/1	0/0	0/0	0/0	0/0	0/0	0/0	0/0	0/0	0/0	0/0	INTERGENIC(MODIFIER)
10'746'774	A	G	0/1	0/0	0/0	0/0	0/0	0/0	0/0	0/0	0/0	0/0	0/0	INTERGENIC(MODIFIER)
10'747'464	TACTCAGCCCTC	T	0/1	0/0	0/0	0/0	0/0	0/0	0/0	0/0	0/0	0/0	0/0	INTERGENIC(MODIFIER)
10'749'184	C	T	0/1	0/0	0/0	0/0	0/0	0/0	0/0	0/0	0/0	0/0	0/0	INTERGENIC(MODIFIER)
10'751'146	A	G	0/1	0/0	0/0	0/0	0/0	0/0	0/0	0/0	0/0	0/0	0/0	INTERGENIC(MODIFIER)
10'752'812	A	G	0/1	0/0	0/0	0/0	0/0	0/0	0/0	0/0	0/0	0/0	0/0	INTERGENIC(MODIFIER)
10'753'127	C	T	0/1	0/0	0/0	0/0	0/0	0/0	0/0	0/0	0/0	0/0	0/0	INTERGENIC(MODIFIER)
10'754'659	G	A	0/1	0/0	0/0	0/0	0/0	0/0	0/0	0/0	0/0	0/0	0/0	INTERGENIC(MODIFIER)
10'754'696	C	T	0/1	0/0	0/0	0/0	0/0	0/0	0/0	0/0	0/0	0/0	0/0	INTERGENIC(MODIFIER)
10'756'127	A	G	0/1	0/0	0/0	0/0	0/0	0/0	0/0	0/0	0/0	0/0	0/0	INTERGENIC(MODIFIER)
10'756'985	G	A	0/1	0/0	0/0	0/0	0/0	0/0	0/0	0/0	0/0	0/0	0/0	INTERGENIC(MODIFIER)
10'758'288	AG	A	0/1	0/0	0/0	0/0	0/0	0/0	0/0	0/0	0/0	0/0	0/0	INTERGENIC(MODIFIER)
10'760'184	C	T	0/1	0/0	0/0	0/0	0/0	0/0	0/0	0/0	0/0	0/0	0/0	INTERGENIC(MODIFIER)
10'763'194	G	A	0/1	0/0	0/0	0/0	0/0	0/0	0/0	0/0	0/0	0/0	0/0	INTERGENIC(MODIFIER)
10'763'657	A	G	0/1	0/0	0/0	0/0	0/0	0/0	0/0	0/0	0/0	0/0	0/0	INTERGENIC(MODIFIER)
10'765'302	G	A	0/1	0/0	0/0	0/0	0/0	0/0	0/0	0/0	0/0	0/0	0/0	INTERGENIC(MODIFIER)
10'765'561	T	C	0/1	0/0	0/0	0/0	0/0	0/0	0/0	0/0	0/0	0/0	0/0	INTERGENIC(MODIFIER)
10'765'673	C	T	0/1	0/0	0/0	0/0	0/0	0/0	0/0	0/0	0/0	0/0	0/0	INTERGENIC(MODIFIER)
10'766'352	G	A	0/1	0/0	0/0	0/0	0/0	0/0	0/0	0/0	0/0	0/0	0/0	INTERGENIC(MODIFIER)
10'766'524	T	A	0/1	0/0	0/0	0/0	0/0	0/0	0/0	0/0	0/0	0/0	0/0	INTERGENIC(MODIFIER)
10'766'873	C	G	0/1	0/0	0/0	0/0	0/0	0/0	0/0	0/0	0/0	0/0	0/0	INTERGENIC(MODIFIER)
10'766'932	C	T	0/1	0/0	0/0	0/0	0/0	0/0	0/0	0/0	0/0	0/0	0/0	INTERGENIC(MODIFIER)
10'767'110	T	C	0/1	0/0	0/0	0/0	0/0	0/0	0/0	0/0	0/0	0/0	0/0	INTERGENIC(MODIFIER)
10'767'308	AT	A	0/1	0/0	0/0	0/0	0/0	0/0	0/0	0/0	0/0	0/0	0/0	INTERGENIC(MODIFIER)
10'768'062	C	T	0/1	0/0	0/0	0/0	0/0	0/0	0/0	0/0	0/0	0/0	0/0	INTERGENIC(MODIFIER)
10'770'589	G	A	0/1	0/0	0/0	0/0	0/0	0/0	0/0	0/0	0/0	0/0	0/0	INTERGENIC(MODIFIER)
10'772'371	AT	A	0/1	0/0	0/0	0/0	0/0	0/0	0/0	0/0	0/0	0/0	0/0	INTERGENIC(MODIFIER)
10'774'110	G	A	0/1	0/0	0/0	0/0	0/0	0/0	0/0	0/0	0/0	0/0	0/0	INTERGENIC(MODIFIER)
10'776'160	G	A	0/1	0/0	0/0	0/0	0/0	0/0	0/0	0/0	0/0	0/0	0/0	INTERGENIC(MODIFIER)
10'778'773	A	G	0/1	0/0	0/0	0/0	0/0	0/0	0/0	0/0	0/0	0/0	0/0	INTERGENIC(MODIFIER)
10'783'977	G	A	0/1	0/0	0/0	0/0	0/0	0/0	0/0	0/0	0/0	0/0	0/0	INTERGENIC(MODIFIER)
10'784'993	C	T	0/1	0/0	0/0	0/0	0/0	0/0	0/0	0/0	0/0	0/0	0/0	INTERGENIC(MODIFIER)
10'785'786	G	T	0/1	0/0	0/0	0/0	0/0	0/0	0/0	0/0	0/0	0/0	0/0	INTERGENIC(MODIFIER)
10'786'065	C	T	0/1	0/0	0/0	0/0	0/0	0/0	0/0	0/0	0/0	0/0	0/0	INTERGENIC(MODIFIER)
10'786'474	C	T	0/1	0/0	0/0	0/0	0/0	0/0	0/0	0/0	0/0	0/0	0/0	INTERGENIC(MODIFIER)
10'787'142	C	T	0/1	0/0	0/0	0/0	0/0	0/0	0/0	0/0	0/0	0/0	0/0	INTERGENIC(MODIFIER)
10'787'254	C	T	0/1	0/0	0/0	0/0	0/0	0/0	0/0	0/0	0/0	0/0	0/0	INTERGENIC(MODIFIER)
10'787'504	G	C	0/1	0/0	0/0	0/0	0/0	0/0	0/0	0/0	0/0	0/0	0/0	INTERGENIC(MODIFIER)
10'787'701	T	G	0/1	0/0	0/0	0/0	0/0	0/0	0/0	0/0	0/0	0/0	0/0	INTERGENIC(MODIFIER)
10'787'847	C	T	0/1	0/0	0/0	0/0	0/0	0/0	0/0	0/0	0/0	0/0	0/0	INTERGENIC(MODIFIER)
10'787'996	G	A	0/1	0/0	0/0	0/0	0/0	0/0	0/0	0/0	0/0	0/0	0/0	INTERGENIC(MODIFIER)
10'788'004	A	G	0/1	0/0	0/0	0/0	0/0	0/0	0/0	0/0	0/0	0/0	0/0	INTERGENIC(MODIFIER)
10'788'031	C	T	0/1	0/0	0/0	0/0	0/0	0/0	0/0	0/0	0/0	0/0	0/0	INTERGENIC(MODIFIER)
10'788'351	G	A	0/1	0/0	0/0	0/0	0/0	0/0	0/0	0/0	0/0	0/0	0/0	INTERGENIC(MODIFIER)
10'788'385	C	T	0/1	0/0	0/0	0/0	0/0	0/0	0/0	0/0	0/0	0/0	0/0	INTERGENIC(MODIFIER)
10'788'508	T	C	0/1	0/0	0/0	0/0	0/0	0/0	0/0	0/0	0/0	0/0	0/0	INTERGENIC(MODIFIER)
10'788'799	T	A	0/1	0/0	0/0	0/0	0/0	0/0	0/0	0/0	0/0	0/0	0/0	INTERGENIC(MODIFIER)
10'789'213	A	T	0/1	0/0	0/0	0/0	0/0	0/0	0/0	0/0	0/0	0/0	0/0	INTERGENIC(MODIFIER)
10'789'299	C	T	0/1	0/0	0/0	0/0	0/0	0/0	0/0	0/0	0/0	0/0	0/0	INTERGENIC(MODIFIER)
10'789'426	A	G	0/1	0/0	0/0	0/0	0/0	0/0	0/0	0/0	0/0	0/0	0/0	INTERGENIC(MODIFIER)
10'789'458	A	G	0/1	0/0	0/0	0/0	0/0	0/0	0/0	0/0	0/0	0/0	0/0	INTERGENIC(MODIFIER)
10'790'329	T	C	0/1	0/0	0/0	0/0	0/0	0/0	0/0	0/0	0/0	0/0	0/0	INTERGENIC(MODIFIER)
10'792'101	C	CA	0/1	0/0	0/0	0/0	0/0	0/0	0/0	0/0	0/0	0/0	0/0	INTERGENIC(MODIFIER)
10'792'107	T	C	0/1	0/0	0/0	0/0	0/0	0/0	0/0	0/0	0/0	0/0	0/0	INTERGENIC(MODIFIER)
10'792'160	C	T	0/1	0/0	0/0	0/0	0/0	0/0	0/0	0/0	0/0	0/0	0/0	INTERGENIC(MODIFIER)
10'792'539	G	C	0/1	0/0	0/0	0/0	0/0	0/0	0/0	0/0	0/0	0/0	0/0	INTERGENIC(MODIFIER)
10'793'293	T	G	0/1	0/0	0/0	0/0	0/0	0/0	0/0	0/0	0/0	0/0	0/0	INTERGENIC(MODIFIER)
10'793'730	G	A	0/1	0/0	0/0	0/0	0/0	0/0	0/0	0/0	0/0	0/0	0/0	INTERGENIC(MODIFIER)
10'794'450	G	A	1/1	0/0	0/0	0/0	0/0	0/0	0/0	0/0	0/0	0/0	0/0	INTERGENIC(MODIFIER)
10'794'477	A	G	0/1	0/0	0/0	0/0	0/0	0/0	0/0	0/0	0/0	0/0	0/0	INTERGENIC(MODIFIER)
10'796'635	A	G	0/1	0/0	0/0	0/0	0/0	0/0	0/0	0/0	0/0	0/0	0/0	INTERGENIC(MODIFIER)
10'802'579	G	A	0/1	0/0	0/0	0/0	0/0	0/0	0/0	0/0	0/0	0/0	0/0	INTERGENIC(MODIFIER)
10'808'189	A	G	0/1	0/0	0/0	0/0	0/0	0/0	0/0	0/0	0/0	0/0	0/0	INTERGENIC(MODIFIER)
10'811'860	T	A	0/1	0/0	0/0	0/0	0/0	0/0	0/0	0/0	0/0	0/0	0/0	INTERGENIC(MODIFIER)
10'813'108	A	T	0/1	0/0	0/0	0/0	0/0	0/0	0/0	0/0	0/0	0/0	0/0	INTERGENIC(MODIFIER)
10'813'716	C	T	0/1	0/0	0/0	0/0	0/0	0/0	0/0	0/0	0/0	0/0	0/0	INTERGENIC(MODIFIER)
10'814'086	C	G	0/1	0/0	0/0	0/0	0/0	0/0	0/0	0/0	0/0	0/0	0/0	INTERGENIC(MODIFIER)
10'814'252	T	C	0/1	0/0	0/0	0/0	0/0	0/0	0/0	0/0	0/0	0/0	0/0	INTERGENIC(MODIFIER)
10'815'141	T	C	0/1	0/0	0/0	0/0	0/0	0/0	0/0	0/0	0/0	0/0	0/0	INTERGENIC(MODIFIER)
10'817'181	G	A	0/1	0/0	0/0	0/0	0/0	0/0	0/0	0/0	0/0	0/0	0/0	INTERGENIC(MODIFIER)
10'817'937	A	C	0/1	0/0	0/0	0/0	0/0	0/0	0/0	0/0	0/0	0/0	0/0	INTERGENIC(MODIFIER)
10'821'095	T	C	0/1	0/0	0/0	0/0	0/0	0/0	0/0	0/0	0/0	0/0	0/0	INTERGENIC(MODIFIER)
10'821'455	T	C	0/1	0/0	0/0	0/0	0/0	0/0	0/0	0/0	0/0	0/0	0/0	INTERGENIC(MODIFIER)
10'821'639	A	G	0/1	0/0	0/0	0/0	0/0	0/0	0/0	0/0	0/0	0/0	0/0	INTERGENIC(MODIFIER)
10'821'903	A	C	0/1	0/0	0/0	0/0	0/0	0/0	0/0	0/0	0/0	0/0	0/0	INTERGENIC(MODIFIER)
10'822'545	C	T	0/1	0/0	0/0	0/0	0/0	0/0	0/0	0/0	0/0	0/0	0/0	INTERGENIC(MODIFIER)
10'822'726	T	A	0/1	0/0	0/0	0/0	0/0	0/0	0/0	0/0	0/0	0/0	0/0	INTERGENIC(MODIFIER)
10'823'302	C	T	0/1	0/0	0/0	0/0	0/0	0/0	0/0	0/0	0/0	0/0	0/0	INTERGENIC(MODIFIER)
10'824'812	G	A	0/1	0/0	0/0	0/0	0/0	0/0	0/0	0/0	0/0	0/0	0/0	INTERGENIC(MODIFIER)
10'832'903	G	A	0/1	0/0	0/0	0/0	0/0	0/0	0/0	0/0	0/0	0/0	0/0	INTERGENIC(MODIFIER)
10'834'672	T	C	0/1	0/0	0/0	0/0	0/0	0/0	0/0	0/0	0/0	0/0	0/0	INTERGENIC(MODIFIER)
10'835'621	C	T	0/1	0/0	0/0	0/0	0/0	0/0	0/0	0/0	0/0	0/0	0/0	INTERGENIC(MODIFIER)
10'836'000	G	A	0/1	0/0	0/0	0/0	0/0	0/0	0/0	0/0	0/0	0/0	0/0	INTERGENIC(MODIFIER)
10'836'539	G	T	0/1	0/0	0/0	0/0	0/0	0/0	0/0	0/0	0/0	0/0	0/0	INTERGENIC(MODIFIER)
10'841'283	C	T	0/1	0/0	0/0	0/0	0/0	0/0	0/0	0/0	0/0	0/0	0/0	INTERGENIC(MODIFIER)
10'845'471	C	T	0/1	0/0	0/0	0/0	0/0	0/0	0/0	0/0	0/0	0/0	0/0	INTERGENIC(MODIFIER)
10'850'729	T	A	0/1	0/0	0/0	0/0	0/0	0/0	0/0	0/0	0/0	0/0	0/0	INTERGENIC(MODIFIER)
10'856'580	T	C	0/1	0/0	0/0	0/0	0/0	0/0	0/0	0/0	0/0	0/0	0/0	INTERGENIC(MODIFIER)
10'860'482	A	G	0/1	0/0	0/0	0/0	0/0	0/0	0/0	0/0	0/0	0/0	0/0	INTERGENIC(MODIFIER)
10'861'882	CAG	C	0/1	0/0	0/0	0/0	0/0	0/0	0/0	0/0	0/0	0/0	0/0	INTERGENIC(MODIFIER)
10'867'848	T	A	0/1	0/0	0/0	0/0	0/0	0/0	0/0	0/0	0/0	0/0	0/0	INTERGENIC(MODIFIER)
10'874'190	A	C	0/1	0/0	0/0	0/0	0/0	0/0	0/0	0/0	0/0	0/0	0/0	INTERGENIC(MODIFIER)
10'881'246	A	G	0/1	0/0	0/0	0/0	0/0	0/0	0/0	0/0	0/0	0/0	0/0	INTERGENIC(MODIFIER)
10'882'903	G	A	0/1	0/0	0/0	0/0	./.	0/0	0/0	0/0	0/0	0/0	0/0	INTERGENIC(MODIFIER)
10'888'927	G	A	0/1	0/0	0/0	0/0	0/0	0/0	0/0	0/0	0/0	0/0	0/0	INTERGENIC(MODIFIER)

10'893'548	A	C	0/1	0/0	0/0	0/0	0/0	0/0	0/0	0/0	0/0	0/0	0/0	INTERGENIC(MODIFIER)
10'904'142	GAA	G	0/1	0/0	0/0	0/0	0/0	0/0	0/0	0/0	0/0	0/0	0/0	INTERGENIC(MODIFIER)
10'913'948	T	C	0/1	0/0	0/0	0/0	0/0	0/0	0/0	0/0	0/0	0/0	0/0	INTERGENIC(MODIFIER)
10'917'155	G	A	0/1	0/0	0/0	0/0	0/0	0/0	0/0	0/0	0/0	0/0	0/0	INTERGENIC(MODIFIER)
10'918'118	C	G	0/1	0/0	0/0	0/0	0/0	0/0	0/0	0/0	0/0	0/0	0/0	INTERGENIC(MODIFIER)
10'922'914	T	C	0/1	0/0	0/0	0/0	0/0	0/0	0/0	0/0	0/0	0/0	0/0	INTERGENIC(MODIFIER)
10'929'351	G	A	0/1	0/0	0/0	0/0	0/0	0/0	0/0	0/0	0/0	0/0	0/0	INTERGENIC(MODIFIER)
10'975'115	A	G	0/1	0/0	0/0	0/0	0/0	0/0	0/0	0/0	0/0	0/0	0/0	INTERGENIC(MODIFIER)
10'979'594	C	T	0/1	0/0	0/0	0/0	0/0	0/0	0/0	0/0	0/0	0/0	0/0	INTERGENIC(MODIFIER)
10'984'292	A	G	0/1	0/0	0/0	0/0	0/0	0/0	0/0	0/0	0/0	0/0	0/0	INTERGENIC(MODIFIER)
10'986'951	G	A	0/1	0/0	0/0	0/0	0/0	0/0	0/0	0/0	0/0	0/0	0/0	INTERGENIC(MODIFIER)
10'990'253	A	T	0/1	0/0	0/0	0/0	0/0	0/0	0/0	0/0	0/0	0/0	0/0	INTERGENIC(MODIFIER)
10'999'314	T	G	0/1	0/0	0/0	0/0	0/0	0/0	0/0	0/0	0/0	0/0	0/0	INTERGENIC(MODIFIER)
11'002'826	G	T	0/1	0/0	0/0	0/0	0/0	0/0	0/0	0/0	0/0	0/0	0/0	INTERGENIC(MODIFIER)
11'006'063	CAGAA	C	0/1	0/0	0/0	0/0	0/0	0/0	0/0	0/0	0/0	0/0	0/0	INTERGENIC(MODIFIER)
11'007'174	G	A	0/1	0/0	0/0	0/0	0/0	0/0	0/0	0/0	0/0	0/0	0/0	INTERGENIC(MODIFIER)
11'007'801	C	T	0/1	0/0	0/0	0/0	0/0	0/0	0/0	0/0	0/0	0/0	0/0	INTERGENIC(MODIFIER)
11'008'710	T	C	0/1	0/0	0/0	0/0	0/0	0/0	0/0	0/0	0/0	0/0	0/0	INTERGENIC(MODIFIER)
11'011'116	C	T	0/1	0/0	0/0	0/0	0/0	0/0	0/0	0/0	0/0	0/0	0/0	INTERGENIC(MODIFIER)
11'012'995	A	C	0/1	0/0	0/0	0/0	0/0	0/0	0/0	0/0	0/0	0/0	0/0	INTERGENIC(MODIFIER)
11'015'198	T	A	0/1	0/0	0/0	0/0	0/0	0/0	0/0	0/0	0/0	0/0	0/0	INTERGENIC(MODIFIER)
11'015'855	A	G	0/1	0/0	0/0	0/0	0/0	0/0	0/0	0/0	0/0	0/0	0/0	INTERGENIC(MODIFIER)
11'016'623	A	G	0/1	0/0	0/0	0/0	0/0	0/0	0/0	0/0	0/0	0/0	0/0	INTERGENIC(MODIFIER)
11'024'782	T	C	1/1	0/0	0/0	0/0	0/0	0/0	0/0	0/0	0/0	0/0	0/0	INTERGENIC(MODIFIER)
11'030'558	C	T	0/1	0/0	0/0	0/0	0/0	0/0	0/0	0/0	0/0	0/0	0/0	INTERGENIC(MODIFIER)
11'031'612	T	C	0/1	0/0	0/0	0/0	0/0	0/0	0/0	0/0	0/0	0/0	0/0	INTERGENIC(MODIFIER)
11'032'263	A	G	0/1	0/0	0/0	0/0	0/0	0/0	0/0	0/0	0/0	0/0	0/0	INTERGENIC(MODIFIER)
11'032'539	C	T	0/1	0/0	0/0	0/0	0/0	0/0	0/0	0/0	0/0	0/0	0/0	INTERGENIC(MODIFIER)
11'033'607	T	C	0/1	0/0	0/0	0/0	0/0	0/0	0/0	0/0	0/0	0/0	0/0	INTERGENIC(MODIFIER)
11'034'917	A	G	0/1	0/0	0/0	0/0	0/0	0/0	0/0	0/0	0/0	0/0	0/0	INTERGENIC(MODIFIER)
11'034'987	C	T	0/1	0/0	0/0	0/0	0/0	0/0	0/0	0/0	0/0	0/0	0/0	INTERGENIC(MODIFIER)
11'036'638	A	AT	0/1	0/0	0/0	0/0	0/0	0/0	0/0	0/0	0/0	0/0	0/0	INTERGENIC(MODIFIER)
11'043'258	C	CT	0/1	0/0	0/0	0/0	0/0	0/0	0/0	0/0	0/0	0/0	0/0	INTERGENIC(MODIFIER)
11'043'455	C	T	1/1	0/0	0/0	0/0	0/0	0/0	0/0	0/0	0/0	0/0	0/0	INTERGENIC(MODIFIER)
11'058'605	A	C	0/1	0/0	0/0	0/0	0/0	0/0	0/0	0/0	0/0	0/0	0/0	INTERGENIC(MODIFIER)
11'067'102	G	A	0/1	0/0	0/0	0/0	0/0	0/0	0/0	0/0	0/0	0/0	0/0	INTERGENIC(MODIFIER)
11'075'056	A	T	0/1	0/0	0/0	0/0	0/0	0/0	0/0	0/0	0/0	0/0	0/0	INTERGENIC(MODIFIER)
11'075'543	C	A	0/1	0/0	0/0	0/0	0/0	0/0	0/0	0/0	0/0	0/0	0/0	INTERGENIC(MODIFIER)
11'075'627	G	A	0/1	0/0	0/0	0/0	0/0	0/0	0/0	0/0	0/0	0/0	0/0	INTERGENIC(MODIFIER)
11'076'137	G	A	0/1	0/0	0/0	0/0	0/0	0/0	0/0	0/0	0/0	0/0	0/0	INTERGENIC(MODIFIER)
11'076'233	C	T	0/1	0/0	0/0	0/0	0/0	0/0	0/0	0/0	0/0	0/0	0/0	INTERGENIC(MODIFIER)
11'076'339	C	T	0/1	0/0	0/0	0/0	0/0	0/0	0/0	0/0	0/0	0/0	0/0	INTERGENIC(MODIFIER)
11'077'928	C	T	0/1	0/0	0/0	0/0	0/0	0/0	0/0	0/0	0/0	0/0	0/0	INTERGENIC(MODIFIER)
11'083'661	A	G	0/1	0/0	0/0	0/0	0/0	0/0	0/0	0/0	0/0	0/0	0/0	INTERGENIC(MODIFIER)
11'084'039	A	G	0/1	0/0	0/0	0/0	0/0	0/0	0/0	0/0	0/0	0/0	0/0	INTERGENIC(MODIFIER)
11'084'542	C	T	0/1	0/0	0/0	0/0	0/0	0/0	0/0	0/0	0/0	0/0	0/0	INTERGENIC(MODIFIER)
11'087'842	A	C	0/1	0/0	0/0	0/0	0/0	0/0	0/0	0/0	0/0	0/0	0/0	INTERGENIC(MODIFIER)
11'089'930	G	A	0/1	0/0	0/0	0/0	0/0	0/0	0/0	0/0	0/0	0/0	0/0	INTERGENIC(MODIFIER)
11'098'031	G	A	0/1	0/0	0/0	0/0	0/0	0/0	0/0	0/0	0/0	0/0	0/0	INTERGENIC(MODIFIER)
11'107'341	G	A	0/1	0/0	0/0	0/0	0/0	0/0	0/0	0/0	0/0	0/0	0/0	INTERGENIC(MODIFIER)
11'109'594	T	C	0/1	0/0	0/0	0/0	0/0	0/0	0/0	0/0	0/0	0/0	0/0	INTERGENIC(MODIFIER)
11'109'808	G	A	0/1	0/0	0/0	0/0	0/0	0/0	0/0	0/0	0/0	0/0	0/0	INTERGENIC(MODIFIER)
11'109'888	T	A	0/1	0/0	0/0	0/0	0/0	0/0	0/0	0/0	0/0	0/0	0/0	INTERGENIC(MODIFIER)
11'109'909	T	C	0/1	0/0	0/0	0/0	0/0	0/0	0/0	0/0	0/0	0/0	0/0	INTERGENIC(MODIFIER)
11'109'942	C	T	0/1	0/0	0/0	0/0	0/0	0/0	0/0	0/0	0/0	0/0	0/0	INTERGENIC(MODIFIER)
11'110'017	T	C	0/1	0/0	0/0	0/0	0/0	0/0	0/0	0/0	0/0	0/0	0/0	INTERGENIC(MODIFIER)
11'110'124	A	T	0/1	0/0	0/0	0/0	0/0	0/0	0/0	0/0	0/0	0/0	0/0	INTERGENIC(MODIFIER)
11'110'750	G	A	0/1	0/0	0/0	0/0	0/0	0/0	0/0	0/0	0/0	0/0	0/0	INTERGENIC(MODIFIER)
11'110'769	A	C	0/1	0/0	0/0	0/0	0/0	0/0	0/0	0/0	0/0	0/0	0/0	INTERGENIC(MODIFIER)
11'111'020	C	T	0/1	0/0	0/0	0/0	0/0	0/0	0/0	0/0	0/0	0/0	0/0	INTERGENIC(MODIFIER)
11'111'780	G	A	0/1	0/0	0/0	0/0	0/0	0/0	0/0	0/0	0/0	0/0	0/0	INTERGENIC(MODIFIER)
11'111'920	T	C	0/1	0/0	0/0	0/0	0/0	0/0	0/0	0/0	0/0	0/0	0/0	INTERGENIC(MODIFIER)
11'113'020	TTTTGA	T	0/1	0/0	0/0	0/0	0/0	0/0	0/0	0/0	0/0	0/0	0/0	INTERGENIC(MODIFIER)
11'113'205	G	A	0/1	0/0	0/0	0/0	0/0	0/0	0/0	0/0	0/0	0/0	0/0	INTERGENIC(MODIFIER)
11'114'065	C	G	0/1	0/0	0/0	0/0	0/0	0/0	0/0	0/0	0/0	0/0	0/0	INTERGENIC(MODIFIER)
11'114'350	T	C	0/1	0/0	0/0	0/0	0/0	0/0	0/0	0/0	0/0	0/0	0/0	INTERGENIC(MODIFIER)
11'116'070	TA	T	0/1	0/0	0/0	0/0	0/0	0/0	0/0	0/0	0/0	0/0	0/0	INTERGENIC(MODIFIER)
11'116'139	G	A	0/1	0/0	0/0	0/0	0/0	0/0	0/0	0/0	0/0	0/0	0/0	INTERGENIC(MODIFIER)
11'116'226	A	G	0/1	0/0	0/0	0/0	0/0	0/0	0/0	0/0	0/0	0/0	0/0	INTERGENIC(MODIFIER)
11'117'536	A	C	0/1	0/0	0/0	0/0	0/0	0/0	0/0	0/0	0/0	0/0	0/0	INTERGENIC(MODIFIER)
11'117'743	A	G	0/1	0/0	0/0	0/0	0/0	0/0	0/0	0/0	0/0	0/0	0/0	INTERGENIC(MODIFIER)
11'117'825	A	G	0/1	0/0	0/0	0/0	0/0	0/0	0/0	0/0	0/0	0/0	0/0	INTERGENIC(MODIFIER)
11'118'301	TG	T	0/1	0/0	0/0	0/0	0/0	0/0	0/0	0/0	0/0	0/0	0/0	INTERGENIC(MODIFIER)
11'118'445	T	C	0/1	0/0	0/0	0/0	0/0	0/0	0/0	0/0	0/0	0/0	0/0	INTERGENIC(MODIFIER)
11'122'015	C	T	0/1	0/0	0/0	0/0	0/0	0/0	0/0	0/0	0/0	0/0	0/0	INTERGENIC(MODIFIER),UPSTREAM(MODIFIER||1616||TTC29||rna18479|)
11'123'958	A	G	0/1	0/0	0/0	0/0	0/0	0/0	0/0	0/0	0/0	0/0	0/0	INTRON(MODIFIER|||TTC29||rna18479|1)
11'125'260	G	T	0/1	0/0	0/0	0/0	0/0	0/0	0/0	0/0	0/0	0/0	0/0	INTRON(MODIFIER|||TTC29||rna18479|2)
11'126'022	T	C	0/1	0/0	0/0	0/0	0/0	0/0	0/0	0/0	0/0	0/0	0/0	INTRON(MODIFIER|||TTC29||rna18479|2)
11'126'151	T	G	0/1	0/0	0/0	0/0	0/0	0/0	0/0	0/0	0/0	0/0	0/0	INTRON(MODIFIER|||TTC29||rna18479|2)
11'126'717	A	C	1/1	0/0	0/0	0/0	0/0	0/0	0/0	0/0	0/0	0/0	0/0	INTRON(MODIFIER|||TTC29||rna18479|2)
11'128'831	G	A	0/1	0/0	0/0	0/0	0/0	0/0	0/0	0/0	0/0	0/0	0/0	INTRON(MODIFIER|||TTC29||rna18479|2)
11'129'118	C	G	0/1	0/0	0/0	0/0	0/0	0/0	0/0	0/0	0/0	0/0	0/0	INTRON(MODIFIER|||TTC29||rna18479|2)
11'130'343	A	G	0/1	0/0	0/0	0/0	0/0	0/0	0/0	0/0	0/0	0/0	0/0	INTRON(MODIFIER|||TTC29||rna18479|2)
11'130'695	C	T	0/1	0/0	0/0	0/0	0/0	0/0	0/0	0/0	0/0	0/0	0/0	INTRON(MODIFIER|||TTC29||rna18479|2)
11'130'905	A	G	0/1	0/0	0/0	0/0	0/0	0/0	0/0	0/0	0/0	0/0	0/0	INTRON(MODIFIER|||TTC29||rna18479|2)
11'130'955	C	T	0/1	0/0	0/0	0/0	0/0	0/0	0/0	0/0	0/0	0/0	0/0	INTRON(MODIFIER|||TTC29||rna18479|2)
11'132'007	A	G	0/1	0/0	0/0	0/0	0/0	0/0	0/0	0/0	0/0	0/0	0/0	INTRON(MODIFIER|||TTC29||rna18479|3)
11'132'478	C	T	0/1	0/0	0/0	0/0	0/0	0/0	0/0	0/0	0/0	0/0	0/0	INTRON(MODIFIER|||TTC29||rna18479|3)
11'139'132	G	A	0/1	0/0	0/0	0/0	0/0	0/0	0/0	0/0	0/0	0/0	0/0	INTRON(MODIFIER|||TTC29||rna18479|4)
11'139'403	G	A	0/1	0/0	0/0	0/0	0/0	0/0	0/0	0/0	0/0	0/0	0/0	INTRON(MODIFIER|||TTC29||rna18479|4)
11'140'434	C	T	0/1	0/0	0/0	0/0	0/0	0/0	0/0	0/0	0/0	0/0	0/0	INTRON(MODIFIER|||TTC29||rna18479|4)
11'144'065	C	T	0/1	0/0	0/0	0/0	0/0	0/0	0/0	0/0	0/0	0/0	0/0	INTRON(MODIFIER|||TTC29||rna18479|4)
11'149'582	A	G	0/1	0/0	0/0	0/0	0/0	0/0	0/0	0/0	0/0	0/0	0/0	INTRON(MODIFIER|||TTC29||rna18479|4)
11'151'598	A	AT	0/1	0/0	0/0	0/0	0/0	0/0	0/0	0/0	0/0	0/0	0/0	INTRON(MODIFIER|||TTC29||rna18479|4)
11'152'875	AAAGAC	A	0/1	0/0	0/0	0/0	0/0	0/0	0/0	0/0	0/0	0/0	0/0	INTRON(MODIFIER|||TTC29||rna18479|4)
11'152'926	GAA	G	0/1	0/0	0/0	0/0	0/0	0/0	0/0	0/0	0/0	0/0	0/0	INTRON(MODIFIER|||TTC29||rna18479|4)
11'154'369	A	G	0/1	0/0	0/0	0/0	0/0	0/0	0/0	0/0	0/0	0/0	0/0	INTRON(MODIFIER|||TTC29||rna18479|4)
11'156'898	T	C	0/1	0/0	0/0	0/0	0/0	0/0	0/0	0/0	0/0	0/0	0/0	INTRON(MODIFIER|||TTC29||rna18479|4)
11'157'007	A	C	0/1	0/0	0/0	0/0	0/0	0/0	0/0	0/0	0/0	0/0	0/0	INTRON(MODIFIER|||TTC29||rna18479|4)
11'157'426	G	A	0/1	0/0	0/0	0/0	0/0	0/0	0/0	0/0	0/0	0/0	0/0	INTRON(MODIFIER|||TTC29||rna18479|4)
11'157'597	T	C	0/1	0/0	0/0	0/0	0/0	0/0	0/0	0/0	0/0	0/0	0/0	INTRON(MODIFIER|||TTC29||rna18479|4)
11'158'441	G	C	0/1	0/0	0/0	0/0	0/0	0/0	0/0	0/0	0/0	0/0	0/0	INTRON(MODIFIER|||TTC29||rna18479|4)
11'159'441	C	T	0/1	0/0	0/0	0/0	0/0	0/0	0/0	0/0	0/0	0/0	0/0	INTRON(MODIFIER|||TTC29||rna18479|4)
11'161'545	G	C	0/1	0/0	0/0	0/0	0/0	0/0	0/0	0/0	0/0	0/0	0/0	INTRON(MODIFIER|||TTC29||rna18479|4)
11'161'766	G	A	0/1	0/0	0/0	0/0	0/0	0/0	0/0	0/0	0/0	0/0	0/0	INTRON(MODIFIER|||TTC29||rna18479|4)
11'174'040	A	G	0/1	0/0	0/0	0/0	0/0	0/0	0/0	0/0	0/0	0/0	0/0	INTRON(MODIFIER|||TTC29||rna18479|6)
11'175'360	G	A	0/1	0/0	0/0	0/0	0/0	0/0	0/0	0/0	0/0	0/0	0/0	INTRON(MODIFIER|||TTC29||rna18479|6)
11'178'111	A	G	0/1	0/0	0/0	0/0	0/0	0/0	0/0	0/0	0/0	0/0	0/0	INTRON(MODIFIER|||TTC29||rna18479|6)
11'178'118	A	G	0/1	0/0	0/0	0/0	0/0	0/0	0/0	0/0	0/0	0/0	0/0	INTRON(MODIFIER|||TTC29||rna18479|6)
11'179'924	G	A	0/1	0/0	0/0	0/0	0/0	0/0	0/0	0/0	0/0	0/0	0/0	INTRON(MODIFIER|||TTC29||rna18479|6)
11'181'595	G	A	0/1	0/0	0/0	0/0	0/0	0/0	0/0	0/0	0/0	0/0	0/0	INTRON(MODIFIER|||TTC29||rna18479|6)
11'181'854	C	T	0/1	0/0	0/0	0/0	0/0	0/0	0/0	0/0	0/0	0/0	0/0	INTRON(MODIFIER|||TTC29||rna18479|6)
11'185'132	A	G	0/1	0/0	0/0	0/0	0/0	0/0	0/0	0/0	0/0	0/0	0/0	INTRON(MODIFIER|||TTC29||rna18479|6)
11'186'298	C	T	0/1	0/0	0/0	0/0	0/0	0/0	0/0	0/0	0/0	0/0	0/0	INTRON(MODIFIER|||TTC29||rna18479|6)
11'186'427	G	A	0/1	0/0	0/0	0/0	0/0	0/0	0/0	0/0	0/0	0/0	0/0	INTRON(MODIFIER|||TTC29||rna18479|6)
11'186'519	A	G	0/1	0/0	0/0	0/0	0/0	0/0	0/0	0/0	0/0	0/0	0/0	INTRON(MODIFIER|||TTC29||rna18479|6)
11'186'812	C	A	0/1	0/0	0/0	0/0	0/0	0/0	0/0	0/0	0/0	0/0	0/0	INTRON(MODIFIER|||TTC29||rna18479|6)
11'188'362	C	T	0/1	0/0	0/0	0/0	0/0	0/0	0/0	0/0	0/0	0/0	0/0	INTRON(MODIFIER|||TTC29||rna18479|6)
11'188'490	G	A	0/1	0/0	0/0	0/0	0/0	0/0	0/0	0/0	0/0	0/0	0/0	INTRON(MODIFIER|||TTC29||rna18479|6)
11'188'833	G	A	0/1	0/0	0/0	0/0	0/0	0/0	0/0	0/0	0/0	0/0	0/0	INTRON(MODIFIER|||TTC29||rna18479|6)
11'188'870	G	A	0/1	0/0	0/0	0/0	0/0	0/0	0/0	0/0	0/0	0/0	0/0	INTRON(MODIFIER|||TTC29||rna18479|6)
11'188'935	G	A	0/1	0/0	0/0	0/0	0/0	0/0	0/0	0/0	0/0	0/0	0/0	INTRON(MODIFIER|||TTC29||rna18479|6)
11'189'775	G	A	0/1	0/0	0/0	0/0	0/0	0/0	0/0	0/0	0/0	0/0	0/0	INTRON(MODIFIER|||TTC29||rna18479|6)
11'190'502	A	C	0/1	0/0	0/0	0/0	0/0	0/0	0/0	0/0	0/0	0/0	0/0	INTRON(MODIFIER|||TTC29||rna18479|6)
11'190'560	G	T	0/1	0/0	0/0	0/0	0/0	0/0	0/0	0/0	0/0	0/0	0/0	INTRON(MODIFIER|||TTC29||rna18479|6)
11'190'684	C	T	0/1	0/0	0/0	0/0	0/0	0/0	0/0	0/0	0/0	0/0	0/0	INTRON(MODIFIER|||TTC29||rna18479|6)
11'194'290	G	A	0/1	0/0	0/0	0/0	0/0	0/0	0/0	0/0	0/0	0/0	0/0	INTRON(MODIFIER|||TTC29||rna18479|6)
11'196'783	T	C	0/1	0/0	0/0	0/0	0/0	0/0	0/0	0/0	0/0	0/0	0/0	INTRON(MODIFIER|||TTC29||rna18479|6)
11'197'862	C	T	0/1	0/0	0/0	0/0	0/0	0/0	0/0	0/0	0/0	0/0	0/0	INTRON(MODIFIER|||TTC29||rna18479|6)
11'213'125	G	A	0/1	0/0	0/0	0/0	0/0	0/0	0/0	0/0	0/0	0/0	0/0	INTRON(MODIFIER|||TTC29||rna18479|8)
11'213'341	A	T	0/1	0/0	0/0	0/0	0/0	0/0	0/0	0/0	0/0	0/0	0/0	INTRON(MODIFIER|||TTC29||rna18479|8)
11'225'220	C	G	0/1	0/0	0/0	0/0	0/0	0/0	0/0	0/0	0/0	0/0	0/0	INTRON(MODIFIER|||TTC29||rna18479|8)
11'225'229	C	T	0/1	0/0	0/0	0/0	0/0	0/0	0/0	0/0	0/0	0/0	0/0	INTRON(MODIFIER|||TTC29||rna18479|8)
11'225'294	A	G	0/1	0/0	0/0	0/0	0/0	0/0	0/0	0/0	0/0	0/0	0/0	INTRON(MODIFIER|||TTC29||rna18479|8)
11'225'379	GTTGT	G	0/1	0/0	0/0	0/0	0/0	0/0	0/0	0/0	0/0	0/0	0/0	INTRON(MODIFIER|||TTC29||rna18479|8)
11'225'528	T	G	1/1	0/0	0/0	0/0	0/0	0/0	0/0	0/0	0/0	0/0	0/0	INTRON(MODIFIER|||TTC29||rna18479|8)
11'225'812	A	G	0/1	0/0	0/0	0/0	0/0	0/0	0/0	0/0	0/0	0/0	0/0	INTRON(MODIFIER|||TTC29||rna18479|8)
11'226'052	C	T	0/1	0/0	0/0	0/0	0/0	0/0	0/0	0/0	0/0	0/0	0/0	INTRON(MODIFIER|||TTC29||rna18479|8)
11'228'063	T	G	0/1	0/0	0/0	0/0	0/0	0/0	0/0	0/0	0/0	0/0	0/0	INTRON(MODIFIER|||TTC29||rna18479|8)
11'234'053	A	G	0/1	0/0	0/0	0/0	0/0	0/0	0/0	0/0	0/0	0/0	0/0	INTRON(MODIFIER|||TTC29||rna18479|8)
11'235'264	T	C	0/1	0/0	0/0	0/0	0/0	0/0	0/0	0/0	0/0	0/0	0/0	INTRON(MODIFIER|||TTC29||rna18479|8)
11'240'769	AT	A	0/1	0/0	0/0	0/0	0/0	0/0	0/0	0/0	0/0	0/0	0/0	INTRON(MODIFIER|||TTC29||rna18479|8)
11'240'773	C	A	0/1	0/0	0/0	0/0	0/0	0/0	0/0	0/0	0/0	0/0	0/0	INTRON(MODIFIER|||TTC29||rna18479|8)

11'243'343	A	T	0/1	0/0	0/0	0/0	0/0	0/0	0/0	0/0	0/0	0/0	0/0	INTRON(MODIFIER|||TTC29||rna18479|8)
11'250'270	TCAGA	T	0/1	0/0	0/0	0/0	0/0	0/0	0/0	0/0	0/0	0/0	0/0	INTRON(MODIFIER|||TTC29||rna18479|8)
11'251'428	A	G	0/1	0/0	0/0	0/0	0/0	0/0	0/0	0/0	0/0	0/0	0/0	INTRON(MODIFIER|||TTC29||rna18479|8)
11'255'327	C	T	0/1	0/0	0/0	0/0	0/0	0/0	0/0	0/0	0/0	0/0	0/0	INTRON(MODIFIER|||TTC29||rna18479|9)
11'256'869	G	C	0/1	0/0	0/0	0/0	0/0	0/0	0/0	0/0	0/0	0/0	0/0	INTRON(MODIFIER|||TTC29||rna18479|9)
11'257'024	C	T	0/1	0/0	0/0	0/0	0/0	0/0	0/0	0/0	0/0	0/0	0/0	INTRON(MODIFIER|||TTC29||rna18479|9)
11'258'055	G	A	0/1	0/0	0/0	0/0	0/0	0/0	0/0	0/0	0/0	0/0	0/0	INTRON(MODIFIER|||TTC29||rna18479|9)
11'261'731	T	C	0/1	0/0	0/0	0/0	0/0	0/0	0/0	0/0	0/0	0/0	0/0	INTRON(MODIFIER|||TTC29||rna18479|9)
11'262'955	T	G	0/1	0/0	0/0	0/0	0/0	0/0	0/0	0/0	0/0	0/0	0/0	INTRON(MODIFIER|||TTC29||rna18479|9)
11'265'776	G	C	0/1	0/0	0/0	0/0	0/0	0/0	0/0	0/0	0/0	0/0	0/0	INTRON(MODIFIER|||TTC29||rna18479|9)
11'268'851	C	T	0/1	0/0	0/0	0/0	0/0	0/0	0/0	0/0	0/0	0/0	0/0	INTRON(MODIFIER|||TTC29||rna18479|9)
11'269'174	T	A	0/1	0/0	0/0	0/0	0/0	0/0	0/0	0/0	0/0	0/0	0/0	INTRON(MODIFIER|||TTC29||rna18479|9)
11'270'411	G	A	0/1	0/0	0/0	0/0	0/0	0/0	0/0	0/0	0/0	0/0	0/0	INTRON(MODIFIER|||TTC29||rna18479|10)
11'271'487	G	A	0/1	0/0	0/0	0/0	0/0	0/0	0/0	0/0	0/0	0/0	0/0	INTRON(MODIFIER|||TTC29||rna18479|10)
11'279'009	AAAAACTCTATATTC	A	0/1	0/0	0/0	0/0	0/0	0/0	0/0	0/0	0/0	0/0	0/0	INTRON(MODIFIER|||TTC29||rna18479|11)
11'281'884	G	T	0/1	0/0	0/0	0/0	0/0	0/0	0/0	0/0	0/0	0/0	0/0	INTRON(MODIFIER|||TTC29||rna18479|11)
11'282'952	T	C	0/1	0/0	0/0	0/0	0/0	0/0	0/0	0/0	0/0	0/0	0/0	INTRON(MODIFIER|||TTC29||rna18479|11)
11'283'137	G	T	0/1	0/0	0/0	0/0	0/0	0/0	0/0	0/0	0/0	0/0	0/0	INTRON(MODIFIER|||TTC29||rna18479|11)
11'284'475	A	C	0/1	0/0	0/0	0/0	0/0	0/0	0/0	0/0	0/0	0/0	0/0	INTRON(MODIFIER|||TTC29||rna18479|11)
11'286'209	A	G	0/1	0/0	0/0	0/0	0/0	0/0	0/0	0/0	0/0	0/0	0/0	INTRON(MODIFIER|||TTC29||rna18479|11)
11'294'945	G	A	0/1	0/0	0/0	0/0	0/0	0/0	0/0	0/0	0/0	0/0	0/0	INTRON(MODIFIER|||TTC29||rna18479|11)
11'295'256	G	A	0/1	0/0	0/0	0/0	0/0	0/0	0/0	0/0	0/0	0/0	0/0	INTRON(MODIFIER|||TTC29||rna18479|11)
11'299'256	TAAAAAAAAAAAAAA	T	0/1	0/0	0/0	0/0	0/0	0/0	0/0	0/0	0/0	0/0	0/0	INTRON(MODIFIER|||TTC29||rna18479|11)
11'301'125	C	T	1/1	0/0	0/0	0/0	0/0	0/0	0/0	0/0	0/0	0/0	0/0	INTRON(MODIFIER|||TTC29||rna18479|11)
11'306'584	T	G	0/1	0/0	0/0	0/0	0/0	0/0	0/0	0/0	0/0	0/0	0/0	INTRON(MODIFIER|||TTC29||rna18479|11)
11'307'266	A	G	0/1	0/0	0/0	0/0	0/0	0/0	0/0	0/0	0/0	0/0	0/0	INTRON(MODIFIER|||TTC29||rna18479|11)
11'309'017	A	G	0/1	0/0	0/0	0/0	0/0	0/0	0/0	0/0	0/0	0/0	0/0	INTRON(MODIFIER|||TTC29||rna18479|11)
11'309'060	C	T	0/1	0/0	0/0	0/0	0/0	0/0	0/0	0/0	0/0	0/0	0/0	INTRON(MODIFIER|||TTC29||rna18479|11)
11'309'271	AAATT	A	0/1	0/0	0/0	0/0	0/0	0/0	0/0	0/0	0/0	0/0	0/0	INTRON(MODIFIER|||TTC29||rna18479|11)
11'309'568	TAC	T	0/1	0/0	0/0	0/0	0/0	0/0	0/0	0/0	0/0	0/0	0/0	INTRON(MODIFIER|||TTC29||rna18479|11)
11'310'129	A	G	0/1	0/0	0/0	0/0	0/0	0/0	0/0	0/0	0/0	0/0	0/0	INTRON(MODIFIER|||TTC29||rna18479|11)
11'312'144	C	T	0/1	0/0	0/0	0/0	0/0	0/0	0/0	0/0	0/0	0/0	0/0	INTRON(MODIFIER|||TTC29||rna18479|11)
11'313'746	G	A	0/1	0/0	0/0	0/0	0/0	0/0	0/0	0/0	0/0	0/0	0/0	INTRON(MODIFIER|||TTC29||rna18479|11)
11'315'879	G	A	0/1	0/0	0/0	0/0	0/0	0/0	0/0	0/0	0/0	0/0	0/0	INTRON(MODIFIER|||TTC29||rna18479|11)
11'316'718	AAACT	A	0/1	0/0	0/0	0/0	0/0	0/0	0/0	0/0	0/0	0/0	0/0	INTRON(MODIFIER|||TTC29||rna18479|11)
11'320'830	G	A	0/1	0/0	0/0	0/0	0/0	0/0	0/0	./.	./.	0/0	./.	INTRON(MODIFIER|||TTC29||rna18479|11)
11'321'087	G	A	0/1	0/0	0/0	0/0	0/0	0/0	0/0	./.	./.	0/0	./.	INTRON(MODIFIER|||TTC29||rna18479|11)
11'321'764	CA	C	0/1	0/0	0/0	0/0	0/0	0/0	0/0	0/0	0/0	0/0	0/0	INTRON(MODIFIER|||TTC29||rna18479|11)
11'322'217	C	T	0/1	0/0	0/0	0/0	0/0	0/0	0/0	0/0	0/0	0/0	0/0	INTRON(MODIFIER|||TTC29||rna18479|11)
11'323'141	T	C	0/1	0/0	0/0	0/0	0/0	0/0	0/0	0/0	0/0	0/0	0/0	INTRON(MODIFIER|||TTC29||rna18479|11)
11'323'468	A	G	0/1	0/0	0/0	0/0	0/0	0/0	0/0	0/0	0/0	0/0	0/0	INTRON(MODIFIER|||TTC29||rna18479|11)
11'323'620	A	G	0/1	0/0	0/0	0/0	0/0	0/0	0/0	0/0	0/0	0/0	0/0	INTRON(MODIFIER|||TTC29||rna18479|11)
11'323'921	A	G	0/1	0/0	0/0	0/0	0/0	0/0	0/0	0/0	0/0	0/0	0/0	INTRON(MODIFIER|||TTC29||rna18479|11)
11'324'769	T	C	0/1	0/0	0/0	0/0	0/0	0/0	0/0	0/0	0/0	0/0	0/0	INTRON(MODIFIER|||TTC29||rna18479|11)
11'325'006	G	A	0/1	0/0	0/0	0/0	0/0	0/0	0/0	0/0	0/0	0/0	0/0	INTRON(MODIFIER|||TTC29||rna18479|11)
11'325'638	AC	A	0/1	0/0	0/0	0/0	0/0	0/0	0/0	0/0	0/0	0/0	0/0	INTRON(MODIFIER|||TTC29||rna18479|11)
11'330'480	A	G	0/1	0/0	0/0	0/0	0/0	0/0	0/0	0/0	0/0	0/0	0/0	INTRON(MODIFIER|||TTC29||rna18479|11)
11'330'930	G	T	0/1	0/0	0/0	0/0	0/0	0/0	0/0	0/0	0/0	0/0	0/0	INTRON(MODIFIER|||TTC29||rna18479|11)
11'333'577	T	A	0/1	0/0	0/0	0/0	0/0	0/0	0/0	0/0	0/0	0/0	0/0	INTRON(MODIFIER|||TTC29||rna18479|11)
11'334'926	T	C	0/1	0/0	0/0	0/0	0/0	0/0	0/0	0/0	0/0	0/0	0/0	INTRON(MODIFIER|||TTC29||rna18479|11)
11'334'959	G	A	0/1	0/0	0/0	0/0	0/0	0/0	0/0	0/0	0/0	0/0	0/0	INTRON(MODIFIER|||TTC29||rna18479|11)
11'344'192	C	T	0/1	0/0	0/0	0/0	0/0	0/0	0/0	0/0	0/0	0/0	0/0	INTRON(MODIFIER|||TTC29||rna18479|11)
11'348'220	A	C	0/1	0/0	0/0	0/0	0/0	0/0	0/0	0/0	0/0	0/0	0/0	INTRON(MODIFIER|||TTC29||rna18479|11)
11'349'250	A	G	0/1	0/0	0/0	0/0	0/0	0/0	0/0	0/0	0/0	0/0	0/0	INTRON(MODIFIER|||TTC29||rna18479|11)
11'349'326	A	C	0/1	0/0	0/0	0/0	0/0	0/0	0/0	0/0	0/0	0/0	0/0	INTRON(MODIFIER|||TTC29||rna18479|11)
11'350'761	G	A	0/1	0/0	0/0	0/0	0/0	0/0	0/0	0/0	0/0	0/0	0/0	INTRON(MODIFIER|||TTC29||rna18479|11)
11'351'700	A	G	0/1	0/0	0/0	0/0	0/0	0/0	0/0	0/0	0/0	0/0	0/0	INTRON(MODIFIER|||TTC29||rna18479|11)
11'353'911	C	G	0/1	0/0	0/0	0/0	0/0	0/0	0/0	0/0	0/0	0/0	0/0	INTRON(MODIFIER|||TTC29||rna18479|11)
11'354'548	A	G	0/1	0/0	0/0	0/0	0/0	0/0	0/0	0/0	0/0	0/0	0/0	INTRON(MODIFIER|||TTC29||rna18479|11)
11'358'606	T	C	0/1	0/0	0/0	0/0	0/0	0/0	0/0	0/0	0/0	0/0	0/0	INTRON(MODIFIER|||TTC29||rna18479|11)
11'359'662	A	G	0/1	0/0	0/0	0/0	0/0	0/0	0/0	0/0	0/0	0/0	0/0	INTRON(MODIFIER|||TTC29||rna18479|11)
11'361'208	A	C	0/1	0/0	0/0	0/0	0/0	0/0	0/0	0/0	0/0	0/0	0/0	INTRON(MODIFIER|||TTC29||rna18479|11)
11'362'770	G	A	0/1	0/0	0/0	0/0	0/0	0/0	0/0	0/0	0/0	0/0	0/0	INTRON(MODIFIER|||TTC29||rna18479|11)
11'363'245	C	T	0/1	0/0	0/0	0/0	0/0	0/0	0/0	0/0	0/0	0/0	0/0	INTRON(MODIFIER|||TTC29||rna18479|11)
11'364'373	C	T	0/1	0/0	0/0	0/0	0/0	0/0	0/0	0/0	0/0	0/0	0/0	INTRON(MODIFIER|||TTC29||rna18479|11)
11'372'466	C	T	0/1	0/0	0/0	0/0	0/0	0/0	0/0	0/0	0/0	0/0	0/0	INTRON(MODIFIER|||TTC29||rna18479|11)
11'374'602	T	C	0/1	0/0	0/0	0/0	0/0	0/0	0/0	0/0	0/0	0/0	0/0	INTRON(MODIFIER|||TTC29||rna18479|11)
11'374'920	G	A	0/1	0/0	0/0	0/0	0/0	0/0	0/0	0/0	0/0	0/0	0/0	INTRON(MODIFIER|||TTC29||rna18479|11)
11'375'738	C	G	0/1	0/0	0/0	0/0	0/0	0/0	0/0	0/0	0/0	0/0	0/0	INTRON(MODIFIER|||TTC29||rna18479|11)
11'376'408	C	A	0/1	0/0	0/0	0/0	0/0	0/0	0/0	0/0	0/0	0/0	0/0	INTRON(MODIFIER|||TTC29||rna18479|11)
11'376'886	C	T	0/1	0/0	0/0	0/0	0/0	0/0	0/0	0/0	0/0	0/0	0/0	INTRON(MODIFIER|||TTC29||rna18479|11)
11'378'945	C	T	0/1	0/0	0/0	0/0	0/0	0/0	0/0	0/0	0/0	0/0	0/0	INTRON(MODIFIER|||TTC29||rna18479|11)
11'381'000	C	T	0/1	0/0	0/0	0/0	0/0	0/0	0/0	0/0	0/0	0/0	0/0	INTRON(MODIFIER|||TTC29||rna18479|11)
11'386'477	G	A	0/1	0/0	0/0	0/0	0/0	0/0	0/0	0/0	0/0	0/0	0/0	DOWNSTREAM(MODIFIER||2682||TTC29||rna18479|),INTERGENIC(MODIFIER)
11'390'299	T	G	0/1	0/0	0/0	0/0	0/0	0/0	0/0	0/0	0/0	0/0	0/0	INTERGENIC(MODIFIER)
11'394'168	A	G	0/1	0/0	0/0	0/0	0/0	0/0	0/0	0/0	0/0	0/0	0/0	INTERGENIC(MODIFIER)
11'394'774	G	C	0/1	0/0	0/0	0/0	0/0	0/0	0/0	0/0	0/0	0/0	0/0	INTERGENIC(MODIFIER)
11'397'569	T	C	0/1	0/0	0/0	0/0	0/0	0/0	0/0	0/0	0/0	0/0	0/0	INTERGENIC(MODIFIER)
11'397'991	A	C	0/1	0/0	0/0	0/0	0/0	0/0	0/0	0/0	0/0	0/0	0/0	INTERGENIC(MODIFIER)
11'398'118	G	A	0/1	0/0	0/0	0/0	0/0	0/0	0/0	0/0	0/0	0/0	0/0	INTERGENIC(MODIFIER)
11'398'701	CTT	C	0/1	0/0	0/0	0/0	0/0	0/0	0/0	0/0	0/0	0/0	0/0	INTERGENIC(MODIFIER)
11'399'897	G	A	0/1	0/0	0/0	0/0	0/0	0/0	0/0	0/0	0/0	0/0	0/0	INTERGENIC(MODIFIER)
11'400'108	C	T	0/1	0/0	0/0	0/0	0/0	0/0	0/0	0/0	0/0	0/0	0/0	INTERGENIC(MODIFIER)
11'400'400	A	ATT	0/1	0/0	0/0	0/0	0/0	0/0	0/0	0/0	0/0	0/0	0/0	INTERGENIC(MODIFIER)
11'400'614	A	G	0/1	0/0	0/0	0/0	0/0	0/0	0/0	0/0	0/0	0/0	0/0	INTERGENIC(MODIFIER)
11'401'138	A	G	0/1	0/0	0/0	0/0	0/0	0/0	0/0	0/0	0/0	0/0	0/0	INTERGENIC(MODIFIER)
11'401'815	T	C	0/1	0/0	0/0	0/0	0/0	0/0	0/0	0/0	0/0	0/0	0/0	INTERGENIC(MODIFIER)
11'403'874	A	G	0/1	0/0	0/0	0/0	0/0	0/0	0/0	0/0	0/0	0/0	0/0	INTERGENIC(MODIFIER)
11'403'918	A	T	0/1	0/0	0/0	0/0	0/0	0/0	0/0	0/0	0/0	0/0	0/0	INTERGENIC(MODIFIER)
11'404'314	G	A	0/1	0/0	0/0	0/0	0/0	0/0	0/0	0/0	0/0	0/0	0/0	INTERGENIC(MODIFIER)
11'406'656	G	A	0/1	0/0	0/0	0/0	0/0	0/0	0/0	0/0	0/0	0/0	0/0	INTERGENIC(MODIFIER)
11'411'734	G	A	0/1	0/0	0/0	0/0	0/0	0/0	0/0	0/0	0/0	0/0	0/0	INTERGENIC(MODIFIER)
11'412'398	G	C	0/1	0/0	0/0	0/0	0/0	0/0	0/0	0/0	0/0	0/0	0/0	INTERGENIC(MODIFIER)
11'413'924	C	T	0/1	0/0	0/0	0/0	0/0	0/0	0/0	0/0	0/0	0/0	0/0	INTERGENIC(MODIFIER)
11'418'533	C	T	0/1	0/0	0/0	0/0	0/0	0/0	0/0	0/0	0/0	0/0	0/0	INTERGENIC(MODIFIER)
11'459'268	A	G	0/1	0/0	0/0	0/0	0/0	0/0	0/0	0/0	0/0	0/0	0/0	INTERGENIC(MODIFIER)
11'465'433	C	T	0/1	0/0	0/0	0/0	0/0	0/0	0/0	0/0	0/0	0/0	0/0	INTERGENIC(MODIFIER)
11'466'004	T	C	0/1	0/0	0/0	0/0	0/0	0/0	0/0	0/0	0/0	0/0	0/0	INTERGENIC(MODIFIER)
11'467'857	T	C	0/1	0/0	0/0	0/0	0/0	0/0	0/0	0/0	0/0	0/0	0/0	INTERGENIC(MODIFIER)
11'469'980	T	C	0/1	0/0	0/0	0/0	0/0	0/0	0/0	0/0	0/0	0/0	0/0	INTERGENIC(MODIFIER)
11'476'085	T	C	0/1	0/0	0/0	0/0	0/0	0/0	0/0	0/0	0/0	0/0	0/0	INTERGENIC(MODIFIER)
11'476'418	G	A	0/1	0/0	0/0	0/0	0/0	0/0	0/0	0/0	0/0	0/0	0/0	INTERGENIC(MODIFIER)
11'480'142	A	C	0/1	0/0	0/0	0/0	0/0	0/0	0/0	0/0	0/0	0/0	0/0	INTERGENIC(MODIFIER)
11'497'082	C	T	0/1	0/0	0/0	0/0	0/0	0/0	0/0	0/0	0/0	0/0	0/0	INTERGENIC(MODIFIER)
11'497'982	C	T	0/1	0/0	0/0	0/0	0/0	0/0	0/0	0/0	0/0	0/0	0/0	INTERGENIC(MODIFIER)
11'502'181	C	T	0/1	0/0	0/0	0/0	0/0	0/0	0/0	0/0	0/0	0/0	0/0	INTERGENIC(MODIFIER)
11'502'563	G	A	0/1	0/0	0/0	0/0	0/0	0/0	0/0	0/0	0/0	0/0	0/0	INTERGENIC(MODIFIER)
11'505'574	T	C	0/1	0/0	0/0	0/0	0/0	0/0	0/0	0/0	0/0	0/0	0/0	INTERGENIC(MODIFIER)
11'507'485	T	C	0/1	0/0	0/0	0/0	0/0	0/0	0/0	0/0	0/0	0/0	0/0	INTERGENIC(MODIFIER)
11'508'577	G	T	0/1	0/0	0/0	0/0	0/0	0/0	0/0	0/0	0/0	0/0	0/0	INTERGENIC(MODIFIER)
11'510'631	G	T	0/1	0/0	0/0	0/0	0/0	0/0	0/0	0/0	0/0	0/0	0/0	INTERGENIC(MODIFIER)
11'511'574	C	T	0/1	0/0	0/0	0/0	0/0	0/0	0/0	0/0	0/0	0/0	0/0	INTERGENIC(MODIFIER)
11'511'655	C	T	0/1	0/0	0/0	0/0	0/0	0/0	0/0	0/0	0/0	0/0	0/0	INTERGENIC(MODIFIER)
11'513'943	T	C	0/1	0/0	0/0	0/0	0/0	0/0	0/0	0/0	0/0	0/0	0/0	INTERGENIC(MODIFIER)
11'514'855	G	T	0/1	0/0	0/0	0/0	0/0	0/0	0/0	0/0	0/0	0/0	0/0	INTERGENIC(MODIFIER)
11'514'919	C	T	0/1	0/0	0/0	0/0	0/0	0/0	0/0	0/0	0/0	0/0	0/0	INTERGENIC(MODIFIER)
11'528'237	T	G	0/1	0/0	0/0	0/0	0/0	0/0	0/0	0/0	0/0	0/0	0/0	INTERGENIC(MODIFIER)
11'533'718	C	T	0/1	0/0	0/0	0/0	0/0	0/0	0/0	0/0	0/0	0/0	0/0	INTERGENIC(MODIFIER),UPSTREAM(MODIFIER||4988||SLC10A7||rna18482|)
11'535'012	AAAT	A	0/1	0/0	0/0	0/0	0/0	0/0	0/0	0/0	0/0	0/0	0/0	INTERGENIC(MODIFIER),UPSTREAM(MODIFIER||3693||SLC10A7||rna18482|)
11'615'430	G	A	0/1	0/0	0/0	0/0	0/0	0/0	0/0	0/0	0/0	0/0	0/0	INTRON(MODIFIER|||SLC10A7||rna18482|4)
11'616'703	G	C	0/1	0/0	0/0	0/0	0/0	0/0	0/0	0/0	0/0	0/0	0/0	INTRON(MODIFIER|||SLC10A7||rna18482|4)
11'616'990	G	A	0/1	0/0	0/0	0/0	0/0	0/0	0/0	0/0	0/0	0/0	0/0	INTRON(MODIFIER|||SLC10A7||rna18482|4)
11'620'465	C	T	0/1	0/0	0/0	0/0	0/0	0/0	0/0	0/0	0/0	0/0	0/0	INTRON(MODIFIER|||SLC10A7||rna18482|4)
11'622'217	A	G	0/1	0/0	0/0	0/0	0/0	0/0	0/0	0/0	0/0	0/0	0/0	INTRON(MODIFIER|||SLC10A7||rna18482|4)
11'622'916	A	G	0/1	0/0	0/0	0/0	0/0	0/0	0/0	0/0	0/0	0/0	0/0	INTRON(MODIFIER|||SLC10A7||rna18482|4)
11'623'914	T	G	0/1	0/0	0/0	0/0	0/0	0/0	0/0	0/0	0/0	0/0	0/0	INTRON(MODIFIER|||SLC10A7||rna18482|4)
11'628'899	T	G	0/1	0/0	0/0	0/0	0/0	0/0	0/0	0/0	0/0	0/0	0/0	INTRON(MODIFIER|||SLC10A7||rna18482|4)
11'631'081	C	T	0/1	0/0	0/0	0/0	0/0	0/0	0/0	0/0	0/0	0/0	0/0	INTRON(MODIFIER|||SLC10A7||rna18482|4)
11'632'210	A	C	0/1	0/0	0/0	0/0	0/0	0/0	0/0	0/0	0/0	0/0	0/0	INTRON(MODIFIER|||SLC10A7||rna18482|4)
11'639'738	A	G	0/1	0/0	0/0	0/0	0/0	0/0	0/0	0/0	0/0	0/0	0/0	INTRON(MODIFIER|||SLC10A7||rna18482|5)
11'639'822	A	C	0/1	0/0	0/0	0/0	0/0	0/0	0/0	0/0	0/0	0/0	0/0	INTRON(MODIFIER|||SLC10A7||rna18482|5)
11'654'781	C	T	0/1	0/0	0/0	0/0	0/0	0/0	0/0	0/0	0/0	0/0	0/0	INTRON(MODIFIER|||SLC10A7||rna18482|5)
11'660'947	G	A	0/1	0/0	0/0	0/0	0/0	0/0	0/0	0/0	0/0	0/0	0/0	INTRON(MODIFIER|||SLC10A7||rna18482|5)
11'672'179	T	C	0/1	0/0	0/0	0/0	0/0	0/0	0/0	0/0	0/0	0/0	0/0	INTRON(MODIFIER|||SLC10A7||rna18482|5)
11'672'439	A	G	0/1	0/0	0/0	0/0	0/0	0/0	0/0	0/0	0/0	0/0	0/0	INTRON(MODIFIER|||SLC10A7||rna18482|5)
11'672'608	G	C	0/1	0/0	0/0	0/0	0/0	0/0	0/0	0/0	0/0	0/0	0/0	INTRON(MODIFIER|||SLC10A7||rna18482|5)
11'673'926	C	T	0/1	0/0	0/0	0/0	0/0	0/0	0/0	0/0	0/0	0/0	0/0	INTRON(MODIFIER|||SLC10A7||rna18482|5)
11'675'853	G	C	0/1	0/0	0/0	0/0	0/0	0/0	0/0	0/0	0/0	0/0	0/0	INTRON(MODIFIER|||SLC10A7||rna18482|5)
11'677'265	G	A	0/1	0/0	0/0	0/0	0/0	0/0	0/0	0/0	0/0	0/0	0/0	INTRON(MODIFIER|||SLC10A7||rna18482|5)
11'685'407	C	T	0/1	0/0	0/0	0/0	0/0	0/0	0/0	0/0	0/0	0/0	0/0	INTRON(MODIFIER|||SLC10A7||rna18482|5)
11'697'020	G	A	0/1	0/0	0/0	0/0	0/0	0/0	0/0	0/0	0/0	0/0	0/0	INTRON(MODIFIER|||SLC10A7||rna18482|5)
11'698'764	G	A	0/1	0/0	0/0	0/0	0/0	0/0	0/0	0/0	0/0	0/0	0/0	INTRON(MODIFIER|||SLC10A7||rna18482|5)
11'701'563	T	C	1/1	0/0	0/0	0/0	0/0	0/0	0/0	0/0	0/0	0/0	0/0	INTRON(MODIFIER|||SLC10A7||rna18482|5)

<table>
<tr><th></th><th></th><th></th><th></th><th></th><th></th><th></th><th></th><th></th><th></th><th></th><th></th><th></th><th></th><th></th></tr>
<tr><td>11'703'468</td><td>A</td><td>G</td><td>0/1</td><td>0/0</td><td>0/0</td><td>0/0</td><td>0/0</td><td>0/0</td><td>0/0</td><td>0/0</td><td>0/0</td><td>0/0</td><td>0/0</td><td>INTRON(MODIFIER|||SLC10A7||rna18482|5)</td></tr>
<tr><td>11'705'778</td><td>A</td><td>C</td><td>0/1</td><td>0/0</td><td>0/0</td><td>0/0</td><td>0/0</td><td>0/0</td><td>0/0</td><td>0/0</td><td>0/0</td><td>0/0</td><td>0/0</td><td>INTRON(MODIFIER|||SLC10A7||rna18482|5)</td></tr>
<tr><td>11'737'453</td><td>G</td><td>T</td><td>0/1</td><td>0/0</td><td>0/0</td><td>0/0</td><td>0/0</td><td>0/0</td><td>0/0</td><td>0/0</td><td>0/0</td><td>0/0</td><td>0/0</td><td>INTRON(MODIFIER|||SLC10A7||rna18482|5)</td></tr>
<tr><td>11'740'330</td><td>C</td><td>T</td><td>0/1</td><td>0/0</td><td>0/0</td><td>0/0</td><td>0/0</td><td>0/0</td><td>0/0</td><td>0/0</td><td>0/0</td><td>0/0</td><td>0/0</td><td>INTRON(MODIFIER|||SLC10A7||rna18482|5)</td></tr>
<tr><td>11'750'289</td><td>T</td><td>C</td><td>0/1</td><td>0/0</td><td>0/0</td><td>0/0</td><td>0/0</td><td>0/0</td><td>0/0</td><td>0/0</td><td>0/0</td><td>0/0</td><td>0/0</td><td>INTRON(MODIFIER|||SLC10A7||rna18482|5)</td></tr>
<tr><td>11'752'419</td><td>T</td><td>C</td><td>0/1</td><td>0/0</td><td>0/0</td><td>0/0</td><td>0/0</td><td>0/0</td><td>0/0</td><td>0/0</td><td>0/0</td><td>0/0</td><td>0/0</td><td>INTRON(MODIFIER|||SLC10A7||rna18482|5)</td></tr>
<tr><td>11'753'235</td><td>C</td><td>T</td><td>0/1</td><td>0/0</td><td>0/0</td><td>0/0</td><td>0/0</td><td>0/0</td><td>0/0</td><td>0/0</td><td>0/0</td><td>0/0</td><td>0/0</td><td>INTRON(MODIFIER|||SLC10A7||rna18482|5)</td></tr>
<tr><td>11'754'248</td><td>A</td><td>C</td><td>1/1</td><td>0/0</td><td>0/0</td><td>0/0</td><td>0/0</td><td>0/0</td><td>0/0</td><td>0/0</td><td>0/0</td><td>0/0</td><td>0/0</td><td>INTRON(MODIFIER|||SLC10A7||rna18482|5)</td></tr>
<tr><td>11'754'362</td><td>C</td><td>G</td><td>0/1</td><td>0/0</td><td>0/0</td><td>0/0</td><td>0/0</td><td>0/0</td><td>0/0</td><td>0/0</td><td>0/0</td><td>0/0</td><td>0/0</td><td>INTRON(MODIFIER|||SLC10A7||rna18482|5)</td></tr>
<tr><td>11'754'742</td><td>A</td><td>G</td><td>0/1</td><td>0/0</td><td>0/0</td><td>0/0</td><td>0/0</td><td>0/0</td><td>0/0</td><td>0/0</td><td>0/0</td><td>0/0</td><td>0/0</td><td>INTRON(MODIFIER|||SLC10A7||rna18482|5)</td></tr>
<tr><td>11'754'898</td><td>AG</td><td>A</td><td>0/1</td><td>0/0</td><td>0/0</td><td>0/0</td><td>0/0</td><td>0/0</td><td>0/0</td><td>0/0</td><td>0/0</td><td>0/0</td><td>0/0</td><td>INTRON(MODIFIER|||SLC10A7||rna18482|5)</td></tr>
<tr><td>11'755'195</td><td>G</td><td>GT</td><td>0/1</td><td>0/0</td><td>0/0</td><td>0/0</td><td>0/0</td><td>0/0</td><td>0/0</td><td>0/0</td><td>0/0</td><td>0/0</td><td>0/0</td><td>INTRON(MODIFIER|||SLC10A7||rna18482|5)</td></tr>
<tr><td>11'755'834</td><td>T</td><td>C</td><td>0/1</td><td>0/0</td><td>0/0</td><td>0/0</td><td>0/0</td><td>0/0</td><td>0/0</td><td>0/0</td><td>0/0</td><td>0/0</td><td>0/0</td><td>INTRON(MODIFIER|||SLC10A7||rna18482|5)</td></tr>
<tr><td>11'756'276</td><td>A</td><td>G</td><td>0/1</td><td>0/0</td><td>0/0</td><td>0/0</td><td>0/0</td><td>0/0</td><td>0/0</td><td>0/0</td><td>0/0</td><td>0/0</td><td>0/0</td><td>INTRON(MODIFIER|||SLC10A7||rna18482|5)</td></tr>
<tr><td>11'757'498</td><td>C</td><td>A</td><td>0/1</td><td>0/0</td><td>0/0</td><td>0/0</td><td>0/0</td><td>0/0</td><td>0/0</td><td>0/0</td><td>0/0</td><td>0/0</td><td>0/0</td><td>INTRON(MODIFIER|||SLC10A7||rna18482|5)</td></tr>
<tr><td>11'802'165</td><td>G</td><td>GT</td><td>0/1</td><td>0/0</td><td>0/0</td><td>0/0</td><td>0/0</td><td>0/0</td><td>0/0</td><td>0/0</td><td>0/0</td><td>0/0</td><td>0/0</td><td>INTRON(MODIFIER|||SLC10A7||rna18482|9)</td></tr>
<tr><td>11'802'888</td><td>T</td><td>C</td><td>0/1</td><td>0/0</td><td>0/0</td><td>0/0</td><td>0/0</td><td>0/0</td><td>0/0</td><td>0/0</td><td>0/0</td><td>0/0</td><td>0/0</td><td>INTRON(MODIFIER|||SLC10A7||rna18482|9)</td></tr>
<tr><td>11'804'205</td><td>A</td><td>G</td><td>0/1</td><td>0/0</td><td>0/0</td><td>0/0</td><td>0/0</td><td>0/0</td><td>0/0</td><td>0/0</td><td>0/0</td><td>0/0</td><td>0/0</td><td>INTRON(MODIFIER|||SLC10A7||rna18482|9)</td></tr>
<tr><td>11'808'752</td><td>A</td><td>G</td><td>0/1</td><td>0/0</td><td>0/0</td><td>0/0</td><td>0/0</td><td>0/0</td><td>0/0</td><td>0/0</td><td>0/0</td><td>0/0</td><td>0/0</td><td>INTRON(MODIFIER|||SLC10A7||rna18482|10)</td></tr>
<tr><td>11'809'466</td><td>A</td><td>T</td><td>0/1</td><td>0/0</td><td>0/0</td><td>0/0</td><td>0/0</td><td>0/0</td><td>0/0</td><td>0/0</td><td>0/0</td><td>0/0</td><td>0/0</td><td>INTRON(MODIFIER|||SLC10A7||rna18482|10)</td></tr>
<tr><td>11'811'384</td><td>G</td><td>A</td><td>0/1</td><td>0/0</td><td>0/0</td><td>0/0</td><td>0/0</td><td>0/0</td><td>0/0</td><td>0/0</td><td>0/0</td><td>0/0</td><td>0/0</td><td>INTRON(MODIFIER|||SLC10A7||rna18482|10)</td></tr>
<tr><td>11'813'654</td><td>C</td><td>T</td><td>0/1</td><td>0/0</td><td>0/0</td><td>0/0</td><td>0/0</td><td>0/0</td><td>0/0</td><td>0/0</td><td>0/0</td><td>0/0</td><td>0/0</td><td>INTRON(MODIFIER|||SLC10A7||rna18482|10)</td></tr>
<tr><td>11'816'924</td><td>C</td><td>T</td><td>0/1</td><td>0/0</td><td>0/0</td><td>0/0</td><td>0/0</td><td>0/0</td><td>0/0</td><td>0/0</td><td>0/0</td><td>0/0</td><td>0/0</td><td>INTRON(MODIFIER|||SLC10A7||rna18482|10)</td></tr>
<tr><td>11'830'690</td><td>C</td><td>A</td><td>0/1</td><td>0/0</td><td>0/0</td><td>0/0</td><td>0/0</td><td>0/0</td><td>0/0</td><td>0/0</td><td>0/0</td><td>0/0</td><td>0/0</td><td>INTRON(MODIFIER|||SLC10A7||rna18482|10)</td></tr>
<tr><td>11'861'143</td><td>A</td><td>G</td><td>0/1</td><td>0/0</td><td>0/0</td><td>0/0</td><td>0/0</td><td>0/0</td><td>0/0</td><td>0/0</td><td>0/0</td><td>0/0</td><td>0/0</td><td>INTERGENIC(MODIFIER)</td></tr>
<tr><td>11'862'285</td><td>GAA</td><td>G</td><td>0/1</td><td>0/0</td><td>0/0</td><td>0/0</td><td>0/0</td><td>0/0</td><td>0/0</td><td>0/0</td><td>0/0</td><td>0/0</td><td>0/0</td><td>INTERGENIC(MODIFIER)</td></tr>
<tr><td>11'896'605</td><td>G</td><td>C</td><td>0/1</td><td>0/0</td><td>0/0</td><td>0/0</td><td>0/0</td><td>0/0</td><td>0/0</td><td>0/0</td><td>0/0</td><td>0/0</td><td>0/0</td><td>INTRON(MODIFIER|||LOC102175607||rna18487|1)</td></tr>
<tr><td>11'905'804</td><td>G</td><td>C</td><td>0/1</td><td>0/0</td><td>0/0</td><td>0/0</td><td>0/0</td><td>0/0</td><td>0/0</td><td>0/0</td><td>0/0</td><td>0/0</td><td>0/0</td><td>INTERGENIC(MODIFIER)</td></tr>
<tr><td>11'972'462</td><td>G</td><td>A</td><td>0/1</td><td>0/0</td><td>0/0</td><td>0/0</td><td>0/0</td><td>0/0</td><td>0/0</td><td>0/0</td><td>0/0</td><td>0/0</td><td>0/0</td><td>INTERGENIC(MODIFIER)</td></tr>
<tr><td>11'974'523</td><td>T</td><td>C</td><td>0/1</td><td>0/0</td><td>0/0</td><td>0/0</td><td>0/0</td><td>0/0</td><td>0/0</td><td>0/0</td><td>0/0</td><td>0/0</td><td>0/0</td><td>INTERGENIC(MODIFIER)</td></tr>
<tr><td>11'986'641</td><td>C</td><td>A</td><td>0/1</td><td>0/0</td><td>0/0</td><td>0/0</td><td>0/0</td><td>0/0</td><td>0/0</td><td>0/0</td><td>0/0</td><td>0/0</td><td>0/0</td><td>INTERGENIC(MODIFIER)</td></tr>
</table>
